# Systematic Review: Anaesthetic Protocols and Management as Confounders in Rodent Blood Oxygen Level Dependent Functional Magnetic Resonance Imaging (BOLD fMRI)–Part A: Effects of Changes in Physiological Parameters

**DOI:** 10.3389/fnins.2020.577119

**Published:** 2020-10-23

**Authors:** Aline R. Steiner, Frédérik Rousseau-Blass, Aileen Schroeter, Sonja Hartnack, Regula Bettschart-Wolfensberger

**Affiliations:** ^1^Section of Anaesthesiology, Department of Clinical and Diagnostic Services, Vetsuisse Faculty, University of Zurich, Zurich, Switzerland; ^2^Department of Clinical Sciences, Faculty of Veterinary Medicine, Université de Montréal, Saint-Hyacinthe, QC, Canada; ^3^Institute for Biomedical Engineering, University of Zurich and ETH Zurich, Zurich, Switzerland; ^4^Section of Epidemiology, Vetsuisse Faculty, University of Zurich, Zurich, Switzerland

**Keywords:** BOLD fMRI, anaesthetic management, anaesthetic monitoring, validity, rat, mouse

## Abstract

**Background:** To understand brain function in health and disease, functional magnetic resonance imaging (fMRI) is widely used in rodent models. Because animals need to be immobilised for image acquisition, fMRI is commonly performed under anaesthesia. The choice of anaesthetic protocols and may affect fMRI readouts, either directly or via changing physiological balance, and thereby threaten the scientific validity of fMRI in rodents.

**Methods:** The present study systematically reviewed the literature investigating the influence of different anaesthesia regimes and changes in physiological parameters as confounders of blood oxygen level dependent (BOLD) fMRI in rats and mice. Four databases were searched, studies selected according to pre-defined criteria, and risk of bias assessed for each study. Results are reported in two separate articles; this part of the review focuses on effects of changes in physiological parameters.

**Results:** A total of 121 publications was included, of which 49 addressed effects of changes in physiological parameters. Risk of bias was high in all included studies. Blood oxygenation [arterial partial pressure of oxygen (p_a_O_2_)], ventilation [arterial partial pressure of carbon dioxide (p_a_CO_2_)] and arterial blood pressure affected BOLD fMRI readouts across various experimental paradigms.

**Conclusions:** Blood oxygenation, ventilation and arterial blood pressure should be monitored and maintained at stable physiological levels throughout experiments. Appropriate anaesthetic management and monitoring are crucial to obtain scientifically valid, reproducible results from fMRI studies in rodent models.

## Introduction

Rats and mice commonly undergo functional magnetic resonance imaging (fMRI) when models of human brain function (physiological as well as pathological) are studied (Martin, 2014; Jonckers et al., 2015; Pan et al., 2015).

As all animal experiments, rodent fMRI studies are subject to harm-benefit analysis. Studies can only be justified when the anticipated benefits outweigh the harms. For studies to generate benefits, the results have to be scientifically valid (Würbel, 2017). However, the “reproducibility crisis” has shown that scientific validity of studies in animal models cannot be taken for granted. Würbel (2017) proposed to assess the scientific validity of studies by the three aspects of construct, internal and external validity. Construct validity is defined as “The degree to which inferences are warranted from the sampling properties of an experiment (e.g., units, settings, treatments and outcomes) to the entities these samples are intended to represent” (Würbel et al., 2014). Internal validity refers to “The extent to which the design, conduct, and analysis of the experiment eliminate the possibility of bias so that the inference of a causal relationship between an experimental treatment and variation in an outcome measure is warranted,” and external validity is defined as the extent to which findings can be generalised (Würbel et al., 2014).

Blood oxygen level dependent (BOLD) fMRI interprets changes in blood oxygenation levels as a surrogate for neuronal activation, based on the mechanism of neurovascular coupling: upon neuronal activation, a feedforward mechanism dilates arterioles and potentially also capillaries to allow more fully oxygenated blood to flow in (Logothetis and Pfeuffer, 2004; Attwell et al., 2010). As the increased supply of oxygen (O_2_) exceeds the increase of O_2_ consumption in activated areas, venous oxygenation locally increases and accordingly, the amount of deoxygenated haemoglobin relative to oxygenated haemoglobin is reduced. The BOLD signal arises from changes in the ratio of deoxy- to oxyhaemoglobin content per unit of brain volume. Deoxyhaemoglobin content is a function of cerebral metabolic rate of oxygen (CMRO_2_), cerebral blood flow (CBF) and cerebral blood volume (CBV) (Kim and Ogawa, 2012). BOLD fMRI is therefore an indirect measure of neural activity, and changes in CMRO_2_, CBF, or CBV may affect signal intensity without an underlying change in neural activity.

As fMRI is susceptible to movement artefacts, animals need to be immobilised for image acquisition. Traditionally, this has been achieved by general anaesthesia and while imaging of conscious animals is gaining popularity (Gao et al., 2017), it is still common practice to image rats and mice under general anaesthesia or sedation (in the following summarised as “anaesthesia”).

Anaesthesia however not only modulates brain function but also affects several aspects of neurovascular coupling: first, neuronal baseline metabolism and thus CMRO_2_ is markedly reduced compared to the awake state (Gao et al., 2017). Second, anaesthetics may modulate the signal cascades responsible for neurovascular coupling on the molecular level (Nakao et al., 2001; Petzold and Murthy, 2011). Third, haemodynamic baseline conditions and vascular reactivity are typically altered under anaesthesia, either as a result of direct drug effects on cerebral vasculature or as a result of systemic cardiovascular and respiratory depression. For example, hypotension below autoregulatory limits reduces cerebral perfusion pressure and may thus reduce the CBF response. Similarly, hypoventilation in spontaneously breathing animals typically results in elevated partial pressure of carbon dioxide (pCO_2_), which induces vasodilation and thus limits maximal vasodilation in response to stimuli. CBV and CBF responses to stimulation are typically slower and have a lower amplitude in anaesthetised animals compared to conscious animals (Gao et al., 2017). The exact “profile” of cardiovascular and respiratory side effects is however drug- and dose-specific and may also vary between individuals.

Given the multitude of mechanisms by which anaesthesia, or more precisely specific anaesthetic protocols (drugs, dosages, and timing of administration) in combination with anaesthetic management (e.g., mechanical ventilation; with the potential to mitigate some side effects of anaesthetics), can influence BOLD fMRI readouts, the question is whether and how scientifically valid results can be obtained from anaesthetised imaging. While it is beyond the scope of this review to discuss construct validity of specific rodent models in which BOLD fMRI is used, anaesthetic protocols and management certainly have the potential to act as confounding factors and thereby impair internal validity of rodent BOLD fMRI studies. The term “confounder” or “confounding factor” is not used in its statistical sense here, but to discern specifically anaesthesia associated sources of bias from general aspects of study design and conduct.

A systematic review of anaesthetic protocols used for pharmacological fMRI (phMRI) found a wide variety of agents, combinations, dosages and respiratory gases used (73 different protocols in 126 studies) (Haensel et al., 2015). As long as effects of anaesthetic protocols and management on BOLD fMRI outcomes are not known well-enough that they could be “subtracted” from measured results (which would be an ambitious endeavour, given the multitude of possible interactions), such a lack of standardisation means that results cannot readily be compared and synthesised in meta-analyses, especially if conflicting.

The aim of this systematic review was to characterise confounding effects of systemic physiological parameters which are often altered under anaesthesia, as well as to characterise the extent of anaesthetic protocol-related differences in BOLD fMRI outcomes. To obtain this information, we systematically searched for studies which have (a) investigated how changes in systemic physiological parameters affected various BOLD outcome measures, and/or (b) directly compared BOLD fMRI results obtained under different anaesthetic protocols (protocols in the meaning of drugs, doses and timepoints of administration) or with awake imaging in adult rats and mice.

Results are presented in two articles, “part a” for effects of physiological parameters and “part b” for anaesthetic protocol comparison. To our knowledge, this is the first systematic review about the impact of anaesthetic protocols and management on BOLD fMRI validity in laboratory rodents.

## Materials and Methods

### Protocol

This systematic review was conducted in accordance with the “Systematic review protocol for animal intervention studies” (de Vries et al., 2015). The protocol is available in Supplementary Material S1.

### Search Strategy

A systematic search strategy was developed with support from the university's library service. Embase, Medline, Scopus and Web of Science were searched in august 2017 for references containing at least one term relating to rodents, fMRI and anaesthesia or physiological parameters (see Table 1) in the title, abstract or keywords. Language was restricted to English, German and French, and a filter for publication year 1990 or later was used.

**Table 1:** Structure of systematic literature search.

**Rodents** rat OR rats OR mouse OR mice OR rodent OR rodents
**MRI** [(MRI OR MRT OR NMR OR “magnetic resonance imaging”) *proximity operator*5 functional] OR fMRI OR BOLD OR “Blood oxygen level dependent”
**Anaesthesia OR physiology** anesthe* OR anaesthe* OR hypercapnia OR hyperoxia OR hypoxia OR apnoea OR “blood pressure” OR hypotension OR hypertension OR autoregulation OR thermoregulation OR “physiological noise” OR “functional connectivity” OR somatosensory OR stimulation OR isoflurane OR sevoflurane OR halothane OR medetomidine OR dexmedetomidine OR alpha-chloralose OR chloralose OR α-chloralose OR urethane OR propofol OR ketamine OR xylazine

*Search terms within one building block were linked with “OR,” building blocks with “AND.” Database-specific syntax, truncation options and proximity operators (“NEAR” for Embase and Web of Science, “adj” for Medline, “W” for Scopus) were used*.

Additional articles found during personal literature search or recommended by colleagues were also included if they fulfilled eligibility criteria.

### Eligibility Criteria and Study Selection

Studies were eligible if they (a) investigated effects of systemic physiological parameters on brain BOLD fMRI results in adult rats or mice, or (b) reported brain BOLD fMRI results of adult rats or mice under different anaesthetic conditions or in anaesthetised vs. awake animals.

Adult was defined as at least 8 weeks of age or 18 g for mice and at least 8 weeks or 200 g for rats and up to 12 months of age for both species. Studies which did not report age or weight of the animals were included if they not explicitly stated that younger (e.g., pups, neonatal, juvenile, adolescent) or older (geriatric, aged) animals were investigated. No restrictions regarding sex, strain or health status of the animals were imposed.

Stimulation studies (peripheral or central stimulation, including phMRI) as well as resting state studies (rsfMRI) were eligible. No restrictions were made regarding outcome measures except that they had to be directly derived from the BOLD signal. Correlations of the BOLD signal with signals from other functional neuroimaging methods, or with measurements of neural activity or cerebral haemodynamics, were however excluded. Studies were not eligible if they applied BOLD fMRI specifically to brain tumors.

The publication had to describe original research. Book chapters, reviews and opinion pieces were excluded. Studies analysing an existing dataset with a novel approach were eligible, as this strategy allows to reduce the number of animals in experiments. Full articles were included as well as short forms (conference papers/abstracts/posters) for which no corresponding full article could be identified.

Study selection was performed by a single reviewer and consisted of two stages. In the first stage, title and abstract were screened for obvious exclusion reasons (title and abstract screening). For the second stage, full text versions of all references were acquired (full text screening). The complete list of inclusion and exclusion criteria is available in Table 2. References for which the decision was not straightforward were discussed with the supervisor (RBW) and all decisions documented.

**Table 2:** Definitive list of inclusion and exclusion criteria.

	**Inclusion criteria**	**Exclusion criteria**
Type of study	•Original research •Short form (conference abstract, poster or paper) or full article •Re-analysis of previously acquired data	•Review •Opinion piece •Book chapters •Lecture/talk •Complete congress proceedings/abstract collections •Study protocol •Short form with corresponding full article •Multiple reporting •Unrecognised duplicate
Type of animals (all limits refer to the timepoint of image acquisition)	•Rats >200 g or 8 weeks and up to 12 months •Mice >18 g or 8 weeks and up to 12 months •If age/weight not described: assumed that animals were adult unless stated otherwise •Both sexes •Any strain •Any health status	•Species other than rat or mouse •Rats <200 g or 8 weeks or >12 months •Mice <18 g or 8 weeks or >12 months •If age/weight not described: animals described as pups/neonatal/juvenile/adolescent/geriatric/aged/old…
Type of interventions	BOLD Fmri Of The Brain With •Comparison of different drugs, doses or timepoints of imaging relative to induction, or anaesthetised vs. awake for same imaging protocol •Alteration of physiological parameters: either deliberately caused by an intervention or closely monitored over time with the explicit intention (mention in abstract) of analyzing the correlation with fMRI signals. •Accepted experimental paradigms •Resting state •Central stimulation paradigms ° Pharmacological ° Electrical ° Optogenetic •Peripheral stimulation ° Electrical ° Mechanical ° Chemical ° Thermic ° Visual ° Auditory ° Olfactory ° Gustatory ° Visceral	•No MRI •Other (f)MRI modalities •Other body regions •BOLD fMRI of brain tumors •BOLD fMRI studies of the brain, but neither comparing anaesthetic protocols nor investigating alterations of physiological parameters
Definition of anaesthetics	•All inhalant anaesthetics (e.g., isoflurane, sevoflurane, halothane) •Barbiturates (e.g., thiopental) •Propofol, alfaxalone •Ketamine, S-ketamine •α-chloralose •Urethane •Xylazine, medetomidine, dexmedetomidine •Acepromazine •Benzodiazepines •Opioids if part of a balanced anaesthesia protocol	•Opioids as sole sedative or as intervention in pain studies
Physiological parameters under investigation	•Arterial blood pressure •Heart rate •Respiratory rate •pCO_2_ •pO_2_ •SpO_2_ •Pulse distension •Body temperature •Hematocrit	•All other parameters, e.g., blood glucose levels •Local temperature of the brain •BOLD response to hyperoxia/hypercapnia as mere application to compare two groups
Interventions to alter physiological parameters	•Blood withdrawal •Fluid supplementation •Pharmacologic manipulation of cardiovascular parameters	•Hyperbaric inspiratory gas
	•Normobaric changes of inspiratory gas composition •Apnoea •Changes in body temperature	
Outcome measures	•Any measure derived from BOLD signal alone	•Correlations of BOLD signal with other modalities (e.g., EEG signal)
Language restrictions	•English •German •French	•All other languages

*The eligibility criteria formulated in the study protocol did not cover all constellations encountered and had to be refined during study selection. The original version is provided in Supplementary Material S1 and adaptations that were made during study selection in Supplementary Material S3*.

Systematic review software, DistillerSR (Evidence Partners, Ottawa, Canada^1^), was used for study selection, data extraction and risk of bias assessment.

### Data Extraction, Risk of Bias Assessment and Synthesis

For each included study, animal characteristics, the exact anaesthetic protocol and physiological parameters monitored were extracted. Study design, eventual surgical steps during preparation, duration of image acquisition, type and timing of stimulations in fMRI measuring response to stimulation (stimulation fMRI), the general data analysis approach, regions of interest and magnetic field strength were also extracted. Studies investigating influences of physiological parameters were furthermore classified as interventional or observational: interventional studies deliberately manipulated physiological parameter values in addition to interventions which are part of the fMRI experiment *per se* (e.g., administration of a vasoconstrictor during electrical forepaw stimulation), whereas observational studies analysed effects of changes naturally occurring during the course of the experiment (e.g., analysing whether activations measured in a phMRI study correlate with eventual blood pressure changes induced by the drug under investigation). In a quarter of included studies, data extraction and risk of bias assessment were performed by two authors independently (ARS, FR) to ensure consistency of data extraction.

To account for the variety of outcome measures used in individual studies, the outcome to be extracted from each study was defined as whether a difference in the respective outcome measures—be it qualitative or quantitative—was observed (a) depending on the value of the physiological parameter under investigation, and/or (b) under different states of anaesthesia. In a free-text format it was then specified for which outcomes a difference was and wasn't observed. If a difference was found only in some of the investigated outcomes (summarised per dataset), or if absolute values were presented without any statement about or discussion of the significance of those results, the effect was classified as “partial.”

Risk of bias was assessed for individual studies using an adapted version of the SYRCLE risk of bias tool (Hooijmans et al., 2014), to our knowledge the only standardised tool for the assessment of risk of bias in animal intervention studies. The adapted version of the tool can be found in Supplementary Material S2. To ensure consistent assessment of studies, rules derived from specific examples were defined and continuously updated. Individual studies were assessed as having a low, unclear or high risk of bias according to the Cochrane Collaboration's tool for assessing risk of bias in randomised trials (Higgins et al., 2011).

Due to the heterogeneity of included outcome measures and the diversity of comparisons in individual studies, a meta-analysis was not feasible. Instead, data was analysed in a structured (narrative) synthesis. Data from rats and mice was initially analysed separately but following the same structure. References were first grouped by type of fMRI (e.g., rsfMRI, fMRI measuring response to a certain type of stimulation). If a reference reported results for more than one type of fMRI, it was allocated to all types of fMRI for which inclusion criteria were fulfilled. Within each type of fMRI, it was then analysed whether studies investigating the same physiological parameter consistently reported effects, and whether the observed effects on BOLD signals were consistent, complementary, or inconsistent. If multiple references analysed the same data set, findings were pooled and summarised per dataset. For the conclusion, findings from all types of fMRI were integrated per parameter, and this is how they are presented in this article.

## Results

### Search Results and Study Characteristics

**Figure 1:**
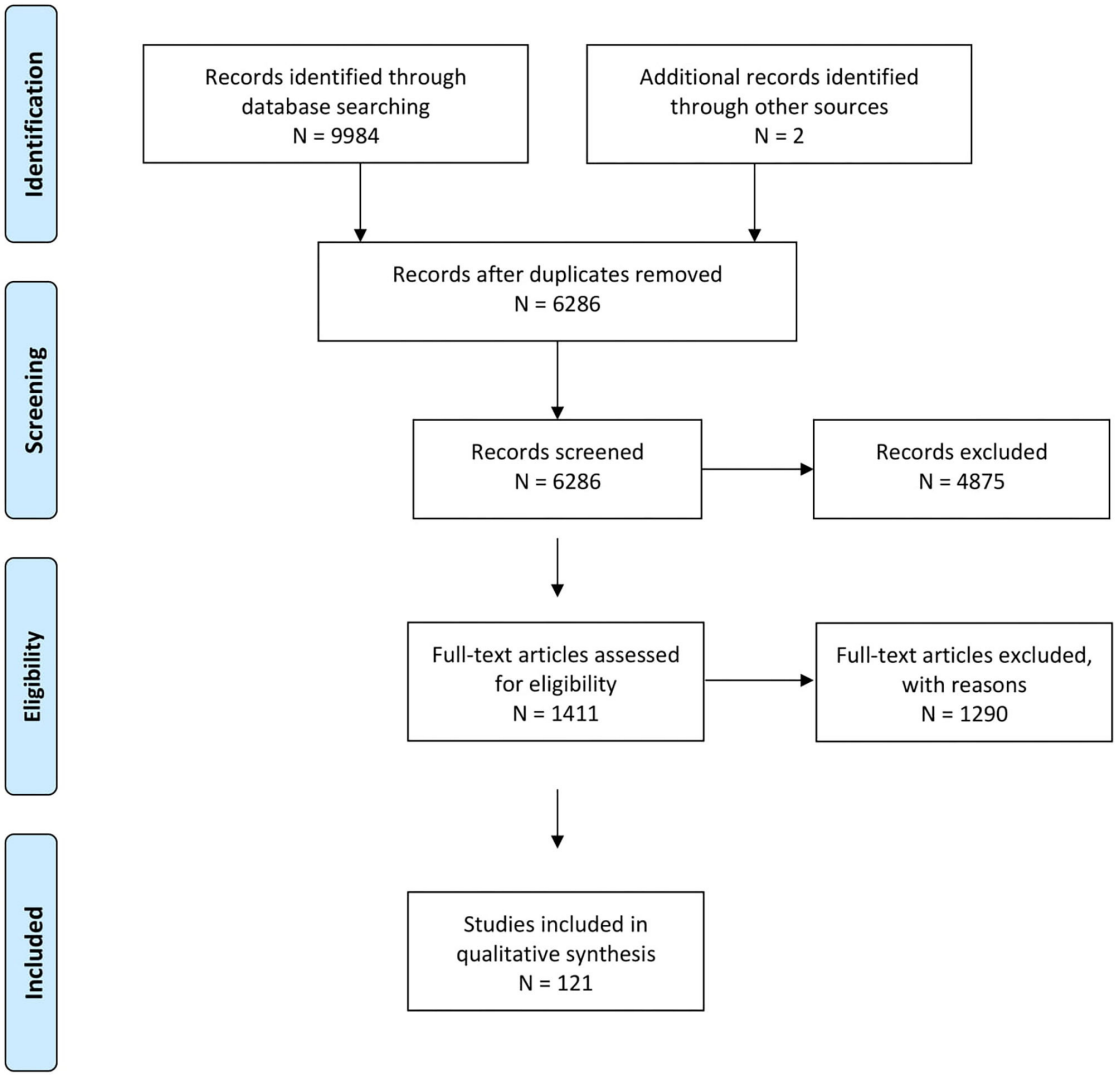
Prisma flow chart representing workflow from reference identification to definitive inclusion.

In total, 6,286 references were identified, of which 121 were finally included (flow chart see Figure 1). The predefined inclusion and exclusion criteria did not cover all constellations encountered. During study selection, additional exclusion criteria were defined: Interventions tested on one animal only were excluded and the results section had to contain at least one sentence about the comparison of interest. Other criteria which were added during study selection are described in Supplementary Material S3. In selected cases, references were excluded for insufficient detail of the reported results, e.g., if descriptive reporting of qualitative aspects was generalised to the point that almost no information could be extracted, such as one sentence about “widespread activation” under one condition without further characterisation of the location, extent or reproducibility of that activation compared to the second experimental condition. Those references were, together with other references for which the decision was not straightforward, documented in Supplementary Material S4 and a brief justification given for each.

Of the 121 included references, 116 were full articles and 5 short forms of publications such as conference abstracts or posters. Those 121 publications were based on 111 datasets. Unless explicitly stated, the number of references corresponds to an equal number of datasets in all following sections.

Rats were studied in 107 references based on 99 datasets and mice in 14 references based on 12 datasets; no publication reported results for both species. Strain and sex distribution are detailed in Supplementary Material S3.

**Figure 2:**
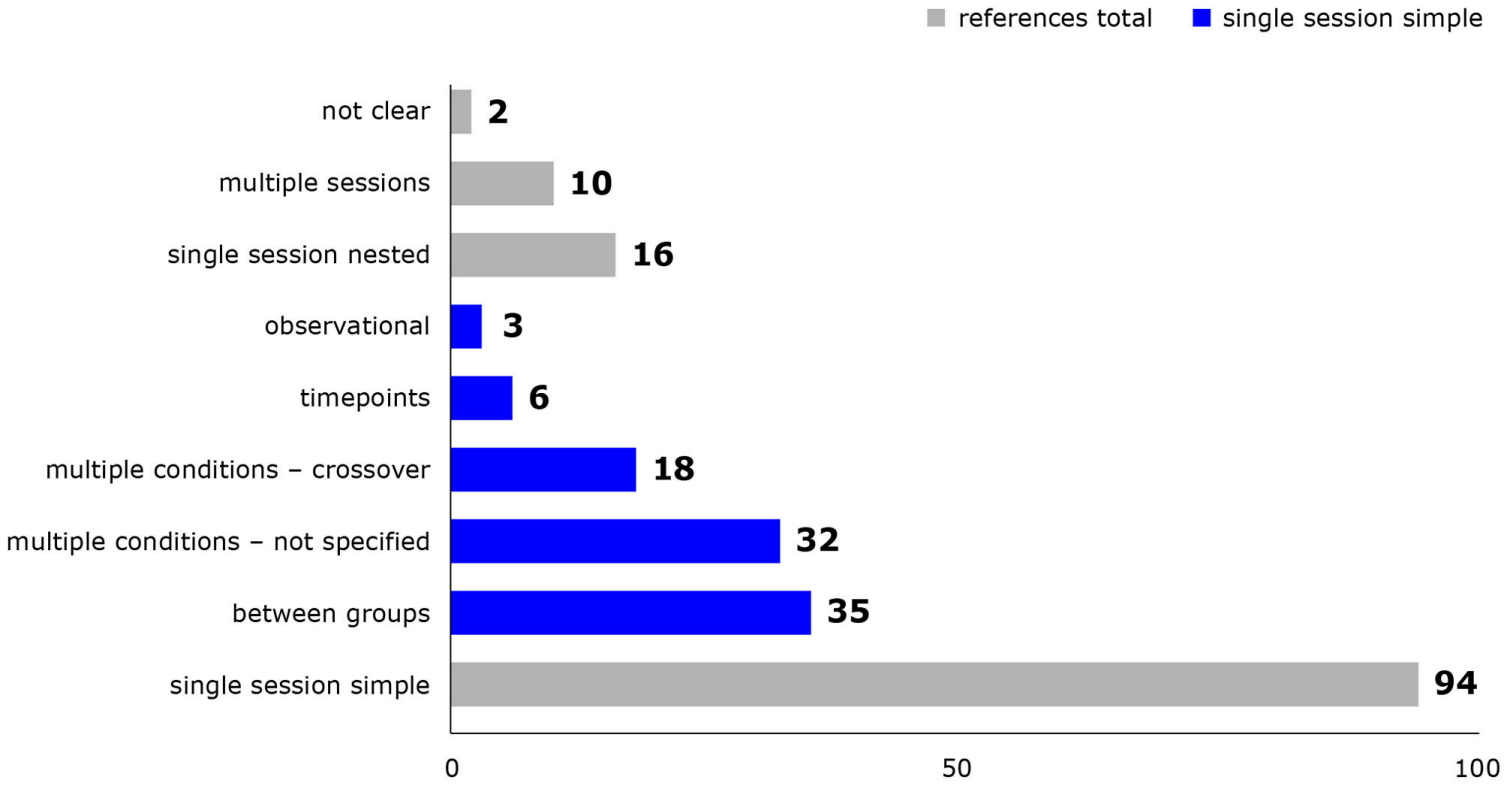
Study designs used in included references. Reference counts are presented, because some references re-analyzed only parts of the original dataset. Single session simple = study design can be described by one of the following categories: between group = one group of animals per condition, one condition per animal; multiple conditions - not specified = multiple conditions per animal in not specified or fixed order; multiple conditions - crossover = multiple conditions per animal in a crossover design; timepoints = one condition per animal, multiple measurements at different timepoints; observational = studies measuring and analyzing the effect of naturally occurring signal fluctuations on BOLD signal. Single session nested = elements of simple study design are combined; multiple sessions = animals underwent several experimental sessions on different days; not clear = the study design was not clear from the provided information.

**Figure 3:**
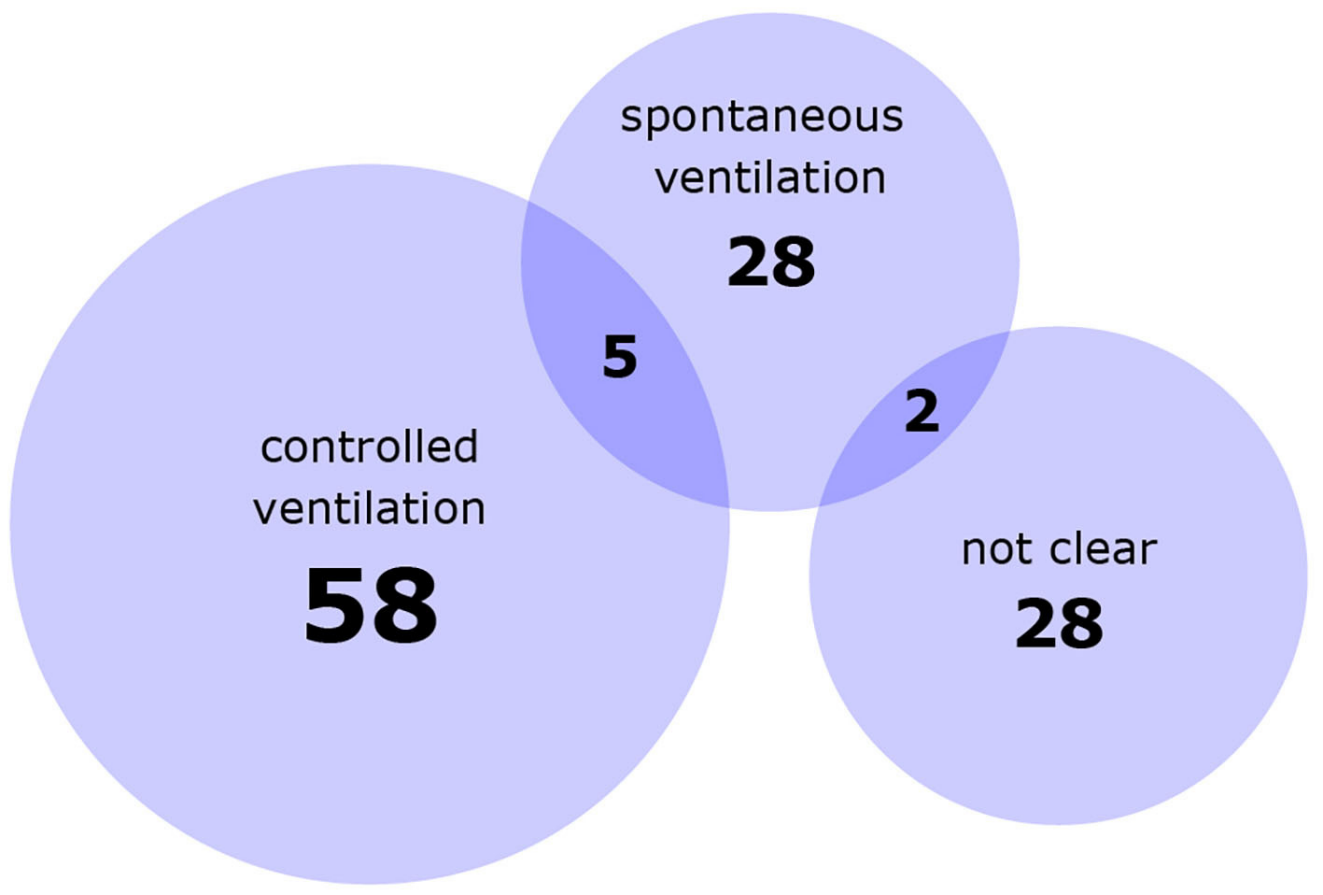
Numbers of references using controlled or spontaneous ventilation in at least one experimental group. Numbers in overlapping areas indicate studies which used both types in different experimental groups. Not clear = the ventilatory management was not explicitly described.

Study designs and details of experimental procedures varied considerably between studies. Most studies either exposed different groups of animals to different conditions or successively exposed the same animals to multiple conditions in a single experimental session, but combinations thereof and multiple experimental sessions were also represented (Figure 2). Animal numbers for fMRI ranged from 2 to 55 rats and from 8 to 63 mice per publication. Experimental procedures, for example whether animals were mechanically ventilated (Figure 3), or which surgical steps were performed prior to fMRI, varied between studies. Tracheotomy was reported in roughly a quarter of included references (32) and a few studies performed surgical procedures on the head directly before fMRI acquisition, such as electrode placement (9), skull exposure (2), middle cerebral artery occlusion (1) or implantation of a head bar (1).

**Figure 4:**
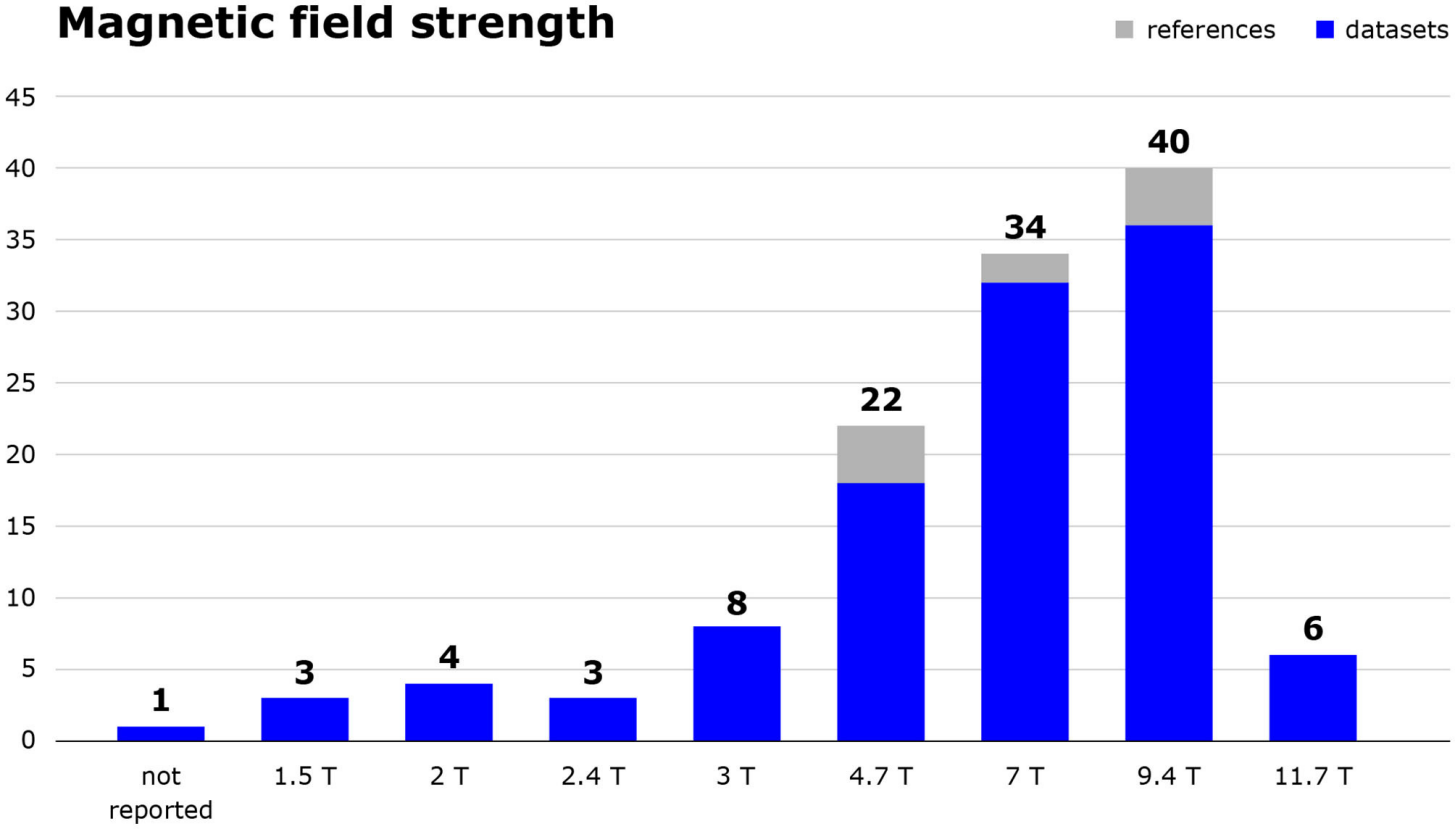
Magnetic field strength in tesla of MRI scanners used in the included studies. The number of included references as well as the number of datasets those publications are based on are shown. Studies using 2.35 T or 7.1 T scanners were categorised as 2.4 and 7.0 T, respectively.

Technical specifications of image acquisition also varied, as is shown for magnetic field strength in Figure 4, and for the pulse sequences and spatial resolutions used in Table 3.

**Table 3:** Overview of type of pulse sequence and spatial resolution used in included studies.

**Refid**	**Authors**	**Year**	**Pulse sequence**		**In-plane spatial resolution**
			**Gradient echo**	**Spin echo**	
13122	Schroeter, A.,Grandjean, J.,Schlegel, F.,Saab, B. J.,Rudin, M.	2017	x		200 × 200
13199	Tsurugizawa, T.,Takahashi, Y.,Kato, F.	2016	x		100 × 100
13362	Nasrallah, F. A.,Yeow, L. Y.,Biswal, B.,Chuang, K. H.	2015		x	400 × 400
13364	Schlegel, F.,Schroeter, A.,Rudin, M.	2015	x		260 × 230
15189	Sedlacik, J.,Reitz, M.,Bolar, D. S.,Adalsteinsson, E.,Schmidt, N. O.,Fiehler, J.	2015	x		250 × 250
13493	Schroeter, A.,Schlegel, F.,Seuwen, A.,Grandjean, J.,Rudin, M.	2014	x		263 × 233
13729	Huang, S.,Du, F.,Shih, Y. Y. I.,Shen, Q.,Gonzalez-Lima, F.,Duong, T. Q.	2013	x		266 × 266
13891	Sumiyoshi, A.,Suzuki, H.,Ogawa, T.,Riera, J. J.,Shimokawa, H.,Kawashima, R.	2012	x		200 × 200
13976	Min, D. K.,Tuor, U. I.,Chelikani, P. K.	2011		x	470 × 470
13996	Kalthoff, D.,Seehafer, J. U.,Po, C.,Wiedermann, D.,Hoehn, M.	2011	x		300 × 300
20115	Baskerville, T. A.,Deuchar, G. A.,McCabe, C.,Robertson, C. A.,Holmes, W. M.,Santosh, C.,Macrae, I. M.	2011	x		260 × 260
15923	Lowry, J. P.,Griffin, K.,McHugh, S. B.,Lowe, A. S.,Tricklebank, M.,Sibson, N. R.	2010	x		470 × 470
14144	Lu, J.,Dai, G.,Egi, Y.,Huang, S.,Kwon, S. J.,Lo, E. H.,Kim, Y. R.	2009	x	(x)	360 × 360
14219	Kannurpatti, S. S.,Biswal, B. B.,Kim, Y. R.,Rosen, B. R.	2008	x		240 × 240
14258	Herman, P.,Sanganahalli, B.,Hyder, F.,Eke, A.	2007		x	Not reported
14268	Tuor, U. I.,Wang, R.,Zhao, Z.,Foniok, T.,Rushforth, D.,Wamsteeker, J. I.,Qiao, M.	2007	x		234 × 234
14304	Qiao, M.,Rushforth, D.,Wang, R.,Shaw, R. A.,Tomanek, B.,Dunn, J. F.,Tuor, U. I.	2007	x		Not reported
14321	Duong, T. Q.	2007	x		310 × 310
14377	Wang, R.,Foniok, T.,Wamsteeker, J. I.,Qiao, M.,Tomanek, B.,Vivanco, R. A.,Tuor, U. I.	2006	x		Not reported
14378	Schmidt, K. F.,Febo, M.,Shen, Q.,Luo, F.,Sicard, K. M.,Ferris, C. F.,Stein, E. A.,Duong, T. Q.	2006	x		360 × 360
14381	Vanhoutte, G.,Verhoye, M.,Van Der Linden, A.	2006	x		150 × 310
14409	Ramos-Cabrer, P.,Weber, R.,Wiedermann, D.,Hoehn, M.	2005		x	400 × 400
14423	Kalisch, R.,Delfino, M.,Murer, M. G.,Auer, D. P.	2005		x	365 × 365
14437	Sicard, K. M.,Duong, T. Q.	2005	x		400 × 400
14502	Kannurpatti, S. S.,Biswal, B. B.	2004	x		540 × 540 (if same FOV as for anatomical images)
14533	Brevard, M. E.,Duong, T. Q.,King, J. A.,Ferris, C. F.	2003	x		200 ×200
14544	Kannurpatti, S. S.,Biswal, B. B.,Hudetz, A. G.	2003	x		540 ×540 (if same FOV as for anatomical images)
14548	Kannurpatti, S. S.,Biswal, B. B.,Hudetz, A. G.	2003	x		540 ×540 (if same FOV as for anatomical images)
14558	Sicard, K.,Shen, Q.,Brevard, M. E.,Sullivan, R.,Ferris, C. F.,King, J. A.,Duong, T. Q.	2003	x		390 × 390
14562	Luo, F.,Wu, G.,Li, Z.,Li, S. J.	2003	x		540 × 540
14569	Tuor, U. I.,McKenzie, E.,Tomanek, B.	2002	x		230 × 230
14584	Dutka, M. V.,Scanley, B. E.,Does, M. D.,Gore, J. C.	2002	x		780 × 780
14589	Kannurpatti,	2002	x		540 × 540 (if same FOV as for anatomical images)
14615	Kalisch, R.,Elbel, G. K.,Gössl, C.,Czisch, M.,Auer, D. P.	2001		x	250 × 250
14654	Xu, H.,Li, S. J.,Bodurka, J.,Zhao, X.,Xi, Z. X.,Stein, E. A.	2000	x		540 ×540
16784	Houston, G. C.,Papadakis, N. G.,Carpenter, T. A.,Hall, L. D.,Mukherjee, B.,James, M. F.,Huang, C. L. H.	2000		x	470 ×470
14670	Zaharchuk, G.,Mandeville, J. B.,Bogdanov Jr, A. A.,Weissleder, R.,Rosen, B. R.,Marota, J. J. A.	1999	x	(x)	780 ×780
14678	Dunn, J. F.,Zaim Wadghiri, Y.,Meyerand, M. E.	1999	x		310 ×310
14685	Hempel, E.,Reith, W.,Elste, V.,Heiland, S.,Sartor, K.	1999	x		310 ×310
14696	Lin, W.,Paczynski, R. P.,Celik, A.,Hsu, C. Y.,Powers, W. J.	1998	x		750 ×480
14702	Hsu, E. W.,Hedlund, L. W.,MacFall, J. R.	1998	x		390 ×390
14703	Lin, W.,Paczynski, R. P.,Celik, A.,Hsu, C. Y.,Powers, W. J.	1998	x		750 ×480
14715	Bock, C.,Schmitz, B.,Kerskens, C. M.,Gyngell, M. L.,Hossmann, K. A.,Hoehn-Berlage, M.	1998	x		375 ×375
14716	Lin, W.,Paczynski, R. P.,Celik, A.,Kuppusamy, K.,Hsu, C. Y.,Powers, W. J.	1998	x		750 ×480
14721	Dunn, J. F.,Swartz, H. M.	1997	x		234 ×234
14734	Kida, I.,Yamamoto, T.,Tamura, M.	1996	x		156 ×156
14746	Graham, G. D.,Zhong, J.,Petroff, O. A. C.,Constable, R. T.,Prichard, J. W.,Gore, J. C.	1994	x		Not reported
22637	Prielmeier, F.,Nagatomo, Y.,Frahm, J.	1994	x		200 ×200
22791	Prielmeier, F.,Merboldt, K. D.,Hanicke, W.,Frahm, J.	1993	x		390 ×200 when 6s acquisition time, 200 ×200 when 12s acquisition time

*The references are sorted in reverse chronological order. (x) means that a study used spin echo sequences in addition to gradient echo*.

The effect of changes in physiological parameters on the BOLD signal was addressed in 45 rat references (45 datasets) and 4 mouse references (4 datasets). Of those, 8 and 3 references, respectively, additionally compared different anaesthetic protocols. Differences in BOLD fMRI measurements between anaesthetic protocols and/or awake and anaesthetised animals were addressed in 70 rat references (62 datasets) and 13 mouse references (11 datasets); findings for anaesthetic protocol comparisons are reported in the second part of this review (part b, under submission). The complete list of included references is provided in Supplementary Material S5.

### Risk of Bias

Risk of bias was assessed as high in all included references. Lack of blinding, both during the experiment (120/121 references clearly not blinded, one reference unclear) and during data analysis (117/121 studies clearly not blinded, 4 studies unclear), was the primary reason for this classification. Not a single publication mentioned randomised, blinded data analysis. Many stated instead that the pipeline of analysis was “fixed” or required minimal input from the operator and was thus free from bias. Apart from blinding, in a substantial percentage of publications concerns associated with study design were present, such as fixed order of conditions, inadequate crossover, differences in fluid administration between experimental groups or insufficient detail of reporting of relevant aspects of study design (28/121 references, 21/111 datasets). Reporting of measures against internal bias was generally low: aspects of sequence generation, allocation concealment and whether the animals underwent the experiment in a randomised order were rarely reported.

### Synthesised Findings

A complete list of included studies investigating effects of changes in physiological parameters on the BOLD signal and BOLD fMRI outcomes is given in Table 4. Notably, only four studies were conducted in mice. References are grouped by type of fMRI; studies which have examined effects on several types of fMRI are highlighted on their second appearance. For each study it is reported which physiological parameters were investigated, whether they deliberately manipulated physiological parameters (i.e., interventional study) or observed “naturally” occuring changes/fluctuations in physiological parameter values during the course of the experiment (i.e., observational study), and whether an effect on the study's outcome measures was observed. “Partial” in this context indicates that a study found effects on some, but not all aspects of the respective outcome, or that the statistical significance of quantitative results was not reported or discussed (e.g., just absolute increase of signal intensity reported).

**Table 4:** Overview of all included studies investigating effects of changes in physiological parameters.

**Type of experiment**	**Sub-type of Experiment**	**References**		**Intervention**										**Observation**							**Comments observational studies**
			**Interventional/observational** ** study**	**hypoxic gas** ** Mixture**	**Hyperoxic gas** ** mixture**	**hypercapnic gas** Mixture ^*^measured ** 20 min after exposure**	**Hypocapnia (induced** ** by hyperventilation)**	**Hyperoxic and hypercapnic** gas mixture ^*^measured 20 min ** after exposure**	Apnoea results for room air, ^*^also for hyperoxic/hyper-capnic/combinded hyperoxic ** and hypercapnic inspiratory gas mixes**	**Arterial blood** ** pressure decrease**	**Arterial blood** ** pressure increase**	**Body temperature**	**Normovolemic** ** haemodilution**	p_**a**_O_**2**_/^*^SpO_**2**_	**p_**a**_ CO_**2**_**	**pH**	Respiratory rate ^*^plus ** waveform and** ** waveform derivative**	**Arterial blood** ** pressure**	Heart rate^*^plus waveform ** and waveform derivative**	**Body temperature**	
Baseline BOLD signal		Baskerville et al. (2011)	i		y																
Baseline BOLD signal		Brevard et al. (2003)	i			y															
Baseline BOLD signal		Dunn and Swartz (1997)	i	p																	
Baseline BOLD signal		Dunn et al. (1999)	i	p																	
Baseline BOLD signal		Duong (2007)	i	p																	
Baseline BOLD signal		Graham et al. (1994)	i			y															
Baseline BOLD signal		Houston et al. (2000)	i	p																	
Baseline BOLD signal		Kalisch et al. (2001)	i							p											
Baseline BOLD signal		Kannurpatti and Biswal (2004)	i						y/y/y/not investigated												
Baseline BOLD signal		Kannurpatti et al. (2002)	i						y/y/not investigated/not investigated												
Baseline BOLD signal		Kannurpatti et al. (2003a)	i		n	p		y	y/y/y/n											
Baseline BOLD signal		Kannurpatti et al. (2003b)	i						y												
Baseline BOLD signal		Kida et al. (1996)	i	p																	
Baseline BOLD signal		Lin et al. (1998a)	i										y								
Baseline BOLD signal		Lin et al. (1998b)	i	p																	
Baseline BOLD signal		Lin et al. (1998c)	i	p																	
Baseline BOLD signal		Lowry et al. (2010)	i	p	p																
Baseline BOLD signal		Lu et al. (2009)	i		y	p															
Baseline BOLD signal		Luo et al. (2003)	i								p										
Baseline BOLD signal		Prielmeier et al. (1993)	i	p																	
Baseline BOLD signal		Prielmeier et al. (1994)	i	p																	
Baseline BOLD signal		Qiao et al. (2007)	i								p										
Baseline BOLD signal		Sicard and Duong (2005)	i	y	y	y															
Baseline BOLD signal		Sicard et al. (2003)	i			p															
Baseline BOLD signal		Tuor et al. (2002)	i								y										
Baseline BOLD signal		Tuor et al. (2007)	i								p										
Baseline BOLD signal		Vanhoutte et al. (2006)	i									y									
Baseline BOLD signal		Wang et al. (2006)	i							y	y										
Baseline BOLD signal		Zaharchuk et al. (1999)	i							n											
Baseline BOLD signal		Tsurugizawa et al. (2016)	o															n			
Baseline BOLD signal		Xu et al. (2000)	o											y	y					
Baseline BOLD signal		Sedlacik et al. (2015)	i		y	y															
periph. stim.	Electrical	Bock et al. (1998)	i			^*^no persistent effect															
periph. stim.	Electrical	Dutka et al. (2002)	i					^*^no persistent effect													
periph. stim.	Electrical	Hempel et al. (1999)	i							p											
periph. stim.	Electrical	Herman et al. (2007)	i							y											
periph. stim.	Electrical	Hsu et al. (1998)	i				y														
periph. stim.	Electrical	Huang et al. (2013)	i	y																	
periph. stim.	Electrical	Nasrallah et al. (2015)	i		y	y		y													
periph. stim.	Electrical	Qiao et al. (2007)	i							p											
periph. stim.	Electrical	Sicard and Duong (2005)	i	p	n	p															
periph. stim.	Electrical	Tuor et al. (2007)	i							p											
periph. stim.	Electrical	Wang et al. (2006)	i							n	y										
periph. stim.	Mechanical visceral	Min et al. (2011)	o															y			
periph. stim.	Electrical	Ramos-Cabrer et al. (2005)	o												y						
periph. stim.	Electrical	Sumiyoshi et al. (2012)	o											n	n	n		n	n		
periph. stim.	Chemical somato-sensory	Tuor et al. (2002)	o															p			
periph. stim.	Electrical	Schroeter et al. (2014)	o											y					y		Relation not quantitatively analysed
periph. stim.	Electrical	Schlegel et al. (2015)	o											x					x		Randomised single pulse stimuli instead of block stimulation no changes in physiological parameters and more specific activation maps
periph. stim.	Electrical	Schroeter et al. (2017)	o											x					x		Bilateral responses to unilateral hindpaw stimulation in acallosal mice
Central stim.	phMRI	Kalisch et al. (2005)	i							y											
Central stim.	phMRI	Schmidt et al. (2006)	o														n	n	n		
Central stim.	phMRI	Luo et al. (2003)	o															n			
Resting state		Nasrallah et al. (2015)	i		y	p		p													
Resting state		Kannurpatti et al. (2008)	i							y											
Resting state		Kalthoff et al. (2011)	o											n^*^			p^*^		p^*^	n	

References are grouped by type of fMRI; studies which have examined effects on several types of fMRI are highlighted in dark gray on their second appearance. Black = rat studies, blue = mouse studies. y = yes, i.e., a clear effect was observed; p = partial, i.e., effects on some, but not all aspects of the respective outcome were found, or the statistical significance of quantitative results was not indicated or discussed; n = no, i.e., no effect was observed; x = see comment for explanation.

* *In the results field: consult top row of the table for interpretation*.

Reference ranges of physiological parameters in awake animals are provided in Table 5, and physiological parameter values measured in the included studies are documented in Table 6.

**Table 5:** Reference ranges of physiological parameters in awake animals (Brun-Pascaud et al., 1982; Irvine et al., 1997; Mattson, 1998; McDougall et al., 2000; Mills et al., 2000; Lee et al., 2009; Bondarenko et al., 2014; Liu and Fan, 2017).

	**p_**a**_O_**2**_ (mmHg)**	**p_**a**_CO_**2**_ (mmHg)**	**Respiratory rate (/min)**	**Heart rate (/min)**	**Arterial blood pressure (mmHg)**
Rats	90 ± 5.5	34.5 ± 3.0	70–110; 85 ± 6	300–500; 336 ± 18 (291-425 over 24h)	Systolic 132 ± 6 (121–141) Diastolic 115 ± 6 (107–124); Mean 103 ± 1
Mice	88 ± 3	39 ± 3	100–200	300–800; 627 ± 21	Mean 90–140

*Values separated by a semicolon are from two different sources*.

**Table 6:** Values of physiological parameters under investigation in the included studies.

**Types of fMRI**	**References**	**Parameter values at baseline**			**Parameter values during intervention**			**Parameter values during observation**					**Effect(s) and comments**
Baseline BOLD signal	Baskerville et al. (2011)	**p**_***a***_**O**_**2**_ 5% O_2_ plus air: 101.8 ± 9.0 5% O_2_ plus air: 101.65 ± 14.81			**p**_**a**_**O**_**2**_ 40% O_2_ plus air: 177.6 ± 30.5 100% O_2_: 445.93 ± 83.98								Yes
Peripheral stimulation, electrical	Bock et al. (1998)	**p**_**a**_**CO**_**2**_ 30% O_2_ plus 70% N_2_ pre exposure: 40.0 ± 2.2 post exposure: 39.3 ± 4.1			**p**_**a**_**CO**_**2**_ 6% CO_2_: 61.5 ± 6								No effect persisting 25 min post exposure
Baseline BOLD signal	Brevard et al. (2003)	**p**_**a**_**CO**_**2**_ Room air Awake: 38 ± 6 Anaesthetised: 49 ± 13			**p**_**a**_**CO**_**2**_ 5% CO_2_ Awake: 47 ± 9 Anaesthetised: 62 ± 18 10% CO_2_ Awake: 88 ± 12 Anaesthetised: 87 ± 8.7								Yes Awake and under Isoflurane
Baseline BOLD signal	(Dunn and Swartz, 1997)	**Not reported**			Not reported Hypoxic gas mixtures, % not reported								Partial
Baseline BOLD signal	Dunn and Swartz (1997)	**Not reported** 30% O_2_			**Not reported** 10, 15 and 20% O_2_								Partial
Baseline BOLD signal	(Duong, 2007)	**Not reported** Room air			**Not reported** 9, 12 and 17% O_2_								Partial Awake and under isoflurane
Peripheral stimulation, electrical	Dutka et al. (2002)	**p**_**a**_**O**_**2**_ 40% O_2_ plus 60% N_2_ pre exposure: 117.3 ±11.7 post exposure (3 runs): 127.8 ± 17.5; 121.5 ± 15.6; 109.0 ± 8.2		**p**_**a**_**CO**_**2**_ 40% O_2_ plus 60% N_2_ pre exposure 31.3 ± 7.7 post exposure (3 runs) 28.5 ± 6.0; 29.0 ± 4.0; 35.3 ± 8.7		**p**_**a**_**O**_**2**_ 90% O_2_ plus 10% CO_2_ 192.5 ± 20.4; 179.8 ±19.9; 175.5 ±19.0	**p**_**a**_**CO**_**2**_ 90% O_2_ plus 10% CO_2_ 60.8 ± 9.6; 60.3 ± 9.8; 59.5 ±7.0						No effect persisting 25 min post exposure
Baseline BOLD signal	Graham et al. (1994)	**Not reported**			**Not reported** 20% CO_2_								Yes
Peripheral stimulation, electrical	Hempel et al. (1999)	**Blood pressure** Systolic: 163 Diastolic: 109			**Blood pressure** blood withdrawal systolic: 81 diastolic: 54								Partial
Peripheral stimulation, electrical	Herman et al. (2007)	**Blood pressure** Not reported			**Blood pressure** negative lower body pressure mean (?): 100, 80, 60, 40								Yes Values most likely mean arterial pressure (MAP), but not specified
Baseline BOLD signal	Houston et al. (2000)	**p**_**a**_**O**_**2**_ Room air 107 ± 12.5			**p**_**a**_**O**_**2**_ 100% N_2_ Not reported								Partial
Peripheral stimulation, electrical	Hsu et al. (1998)	**End-tidal pCO**_**2**_ Mechanical ventilation, baseline settings 36.5 ± 0.4			**End-tidal pCO**_**2**_ Mechanical ventilation, hyperventilation 25.9 ± 0.3								Yes
Peripheral stimulation, electrical	Huang et al. (2013)	**SpO**_**2**_ Room air 94.4 ± 0.8, 94.6 ± 0.7			**SpO**_**2**_ 15% O_2_ 87.6 ± 1.6, 87.5 ± 1.9								Yes
Central stimulation, phMRI	Kalisch et al. (2005)	**Blood pressure** Phosphate-buffered saline mean (?): 113.19 ± 3.38 Phenylephrine mean (?):115.4 ± 1.54			**Blood pressure** Phosphate-buffered saline “BP dropped by – 35% within the first 5 min after APO (apomorphine, the drug under investigation) and then remained between – 15 and – 30% of baseline level until the end of the scan” Phenylephrine “BP changes were limited to a minor (– 10%) and transient (<5 min) BP decrease directly after APO administration”								yes Expected activation from apomorphine only detected when blood pressure stabilised with phenylephrine
Baseline BOLD signal	Kalisch et al. (2001)	**Blood pressure** Isoflurane: mean (?)103.2 ± 4.9 Halothane: mean (?) 111.6 ± 8.1 Propofol: mean (?)133.3 ± 12.3			**Blood pressure** Blood withdrawal Isoflurane:−2.8 to−61.7 (from baseline), average approx.−20 Halothane:−8.1 to−46.1), average approx.−20 Propofol:−4 to−82.6), average approx.−50								Partial
Resting state	Kalthoff et al. (2011)							SpO_2_ respiratory rate (RR) heart rate (HR) temperature (T) respiratory waveforms cardiac waveforms values not reported					SpO_2_, RR and HR and T: no. Excluded from regression because correlated to 1st to 4th order drift terms. Respiratory and cardiac waveforms: partial. Respiratory regressors strong correlations, cardiac regressors correlation “less pronounced”.
Baseline BOLD signal	Kannurpatti et al. (2002)	**SpO**_**2**_ Room air: 96.0 ± 1.0 100% O_2_: 99.8 ± 0.1	**p**_**a**_**O**_**2**_ Room air: 96.3 ± 10.2 100% O_2:_ 330 ± 67.0	**p**_**a**_**CO**_**2**_ Room air: 33.6 ± 7.4 100% O_2_: 34.6 ± 6.5	**SpO**_**2**_ Apnoea Room air: 28.9 ± 5.1 100% O_2_: 99.8 ± 0.	**p**_**a**_**O**_**2**_ Apnoea Room air: 20.4 ± 4.0 100% O_2_: 343 ± 73.2	**p**_**a**_**CO**_**2**_ Apnoea Room air: 47.6 ± 3.9 100% O_2_: 54.2 ± 7.3						Yes (both conditions)
Baseline BOLD signal	Kannurpatti et al. (2003a)	**SpO**_**2**_ Room air: 97.6 ± 0.7 100% O_2_: 99.8 ± 0.1 2% CO_2_: 91.3 ± 2.9 5% CO_2_: 83.9 ± 2 95% O_2_ plus 5 % CO_2_: 99.6 ± 0.1	**p**_**a**_**O**_**2**_ Room air: 96.3 ± 10.2 100% O_2_: 329.8 ± 67.0 2% CO_2_: 70.5 ± 4.6 5% CO_2_: 64.4 ± 5.9 95% O_2_ plus 5 % CO_2_: 305.6 ± 62.5	**p**_**a**_**CO**_**2**_ Room air: 33.6 ± 6.5 100% O_2_: 34.5 ± 6.4 2% CO_2_: 59.6 ± 5.3 5% CO_2_: 95.6 ± 6.5 95% O_2_ plus 5 % CO_2_: 85.9 ± 8.9	**SpO**_**2**_ Apnoea Room air: 28.9 ± 5.1 100% O_2_: 99.8 ± 0.1 2% CO_2_: 24.8 ± 5.9 5% CO_2_: 13.7 ± 5.5 95% O_2_ plus 5% CO_2_: 99.7 ± 0.1	**p**_**a**_**O**_**2**_ Apnoea Room air: 20.4 ± 3.9 100% O_2_: 342.7 ± 73.2 2% CO_2_: 25.5 ± 5.78 5% CO_2_: 19.1 ± 3.8 95% O_2_ plus 5% CO2: 311.3 ± 25.9	**p**_**a**_**CO**_**2**_ Apnoea Room air: 49.6 ± 3.8 100% O_2_: 56.2 ± 7.3 2% CO_2_: 78.3 ± 7.4 5% CO_2_: 108.6 ± 8.8 95% O_2_ plus 5% CO_2_: 121.3 ± 6.0						Hyperoxia: no Hypercapnia: partial Combined hyperoxia and hypercapnia: yes Apnoea: yes/yes/yes/no
Baseline BOLD signal	Kannurpatti et al. (2003b)	**SpO**_**2**_ Room air 97.1 ± 0.9	**p**_**a**_**O**_**2**_ Room air 96.5 ± 8.3	**p**_**a**_**CO**_**2**_ Room air 32.2 ± 6.1	**SpO**_**2**_ Apnoea 20 s: 28.9 ± 5.1 30 s: 17.3 ± 5.7	**p**_**a**_**O**_**2**_ Apnoea 20 s: 24.4 ± 3.9 30 s: 16.2 ± 4.5	**p**_**a**_**CO**_**2**_ Apnoea 20 s: 47.6 ± 3.5 30 s: 53.2 ± 4.1						Yes
Baseline BOLD signal	(Kannurpatti and Biswal, 2004)	**SpO**_**2**_ Room air: 97.6 ± 0.7; 6.0 ± 0.4 100% O_2_: 99.8 ± 0.1; 99.8 ± 0.1 2% CO_2_: 91.3 ± 2.9; 92.5 ± 3.1 5% O_2_: 83.9 ± 2; 85.6 ± 2.6	**p**_**a**_**O**_**2**_ Room air: 96.3 ± 10.2; 80.1 ± 3.2 100% O_2_: 329.8 ± 67.0; 323.0 ± 85.0 2% CO_2_: 70.5 ± 4.6; 71.2 ± 2.5 5% O_2_: 64.4 ± 5.9 66.2 ± 2.0	**p**_**a**_**CO**_**2**_ Room air: 33.5 ± 7.39; 31.7 ± 4.2 100% O_2_: 34.5 ± 6.4; 34.± 5.0 2% CO_2_: 59.6 ± 5.3; 60.1 ± 5.2 5% O_2_: 95.6 ± 6.5; 92.5 ± 6.6	**SpO**_**2**_ Apnoea Room air: 28.9 ± 5.1; 80.1 ± 3.2 100% O_2_: 99.8 ± 0.1; 99.0 ± 0.2 2% CO_2_: 24.8 ± 5.9; 24.8 ± 5.9 5% CO_2_: 13.7 ± 5.5; 13.7 ± 5.5	**p**_**a**_**O**_**2**_ Apnoea Room air: 20.4 ± 3.9; 38.0 ± 6.8 100% O_2_: 42.7 ± 73.2; 76.5 ± 35.4 2% CO_2_: 25.5 ± 5.78; 25.5 ± 5.78 5% CO_2_: 19.1 ± 3.8; 19.1 ± 3.8	**p**_**a**_**CO**_**2**_ Apnoea Room air: 47.6 ± 3.8; 41.6 ± 3.8 100% O_2_: 54.2 ± 7.3; 55.6 ± 3.5 2% CO_2_: 78.3 ± 7.4; 78.3 ± 7.4 5% CO_2_: 108.6 ± 8.8; 108.6 ± 8.8						Apnoea: yes/yes/yes Each condition: first value under pentobarbital, second value under urethane
Resting state	Kannurpatti et al. (2008)	**Blood pressure** Mean 110 ± 10			**Blood pressure** Blood withdrawal Mean 68 ± 7								Yes
Baseline BOLD signal	Kida et al. (1996)	**Not reported** 100% O_2_			**Not reported** 20, 15, 10 and 5% O_2_								Partial
Baseline BOLD signal	Lin et al. (1998a)	**Haematocrit (hct)** 44.6 ± 2.7			**Haematocrit** Mild haemodilution: 33.4 ± 2.1 Moderate haemodilution: 26.2 ± 1.7 %								Yes
Baseline BOLD signal	Lin et al. (1998b)	**SpO**_**2**_ Normal haematocrit (42.83 ±2.33): 90.05 ± 4.05 Mild anaemia (hct 33.4 ± 1.88): 97.72 ± 0.067 Moderate anaemia (hct 27.14 ± 2.7): 98.5 ± 0.44	**p**_**a**_**O**_**2**_ Normal haematocrit (42.83 ±2.33): 94.1 ± 22.15 Mild anaemia (hct 33.4 ± 1.88): 179.9 ± 14.03 Moderate anaemia (hct 27.14 ± 2.7): 179.91 ± 15.69		**SpO**_**2**_ Hypoxic gas mixture; % not reported Normal haematocrit (42.83 ±2.33): 48.89 ± 14.41 Mild anaemia (hct 33.4 ± 1.88): 35.89 ± 10.14 Moderate anaemia (hct 27.14 ± 2.7): 51.95 ± 22.34	**p**_**a**_**O**_**2**_ Hypoxic gas mixture; % not reported Normal haematocrit (42.83 ±2.33%): 38.74 ± 13.93 Mild anaemia (hct 33.4 ± 1.88): 35.08 ± 6.24 Moderate anaemia (hct 27.14 ± 2.7): 56.27 ± 49.9							Partial
Baseline BOLD signal	Lin et al. (1998c)	**SpO**_**2**_ Room air 90.5 ± 4.05	**p**_**a**_**O**_**2**_ Room air 94.1 ± 22.15		**SpO**_**2**_ 10-11% O_2_ 56.4 ± 13.50 4-5% O_2_ 38.1 ± 6.98	**p**_**a**_**O**_**2**_ 10-11% O_2_ 45.0 ± 15.10 4-5% O_2_ 29.7 ± 3.71							Partial
Baseline BOLD signal	Lowry et al. (2010)	**Not reported** 30% O_2_ plus 70% N_2_			**Not reported** 0% O_2_ (100% N_2_); 50, 70, 100% O_2_								Hypoxia: partial Hyperoxia: partial
Baseline BOLD signal	Lu et al. (2009)	**p**_**a**_**O**_**2**_ Room air 95.3 ± 15.1		**p**_**a**_**CO**_**2**_ Room air 34.4 ± 4.5	**p**_**a**_**O**_**2**_ 100% O_2_ 113.2 ± 131.2		**p**_**a**_**CO**_**2**_ 5% CO_2_ 69.8 ± 7.1 partial						Hyperoxia: yes Hypercapnia: partial
Central stimulation, phMRI	Luo et al. (2003)	**Blood pressure** mean 108 ± 8						**Blood pressure** After drug administration 30–80 % increase from baseline					No Relevant activations after cocaine, but not after exclusively peripherally acting cocaine methiodide.
Peripheral stimulation, mechanical visceral	Min et al. (2011)	**Blood pressure** Not reported						**Blood pressure** “peaked at (approx.) 30 mm Hg above control conditions”					Yes “Nonlinear regres- sion analysis revealed that a quadratic model best explained the relationship between changes in BOLD fMRI signal intensity and BP.”
Peripheral stimulation, electrical; resting state	Nasrallah et al. (2015)	**p**_**a**_**O**_**2**_ 47% O2 plus 53% air: 216.8 ± 3.6	**p**_**a**_**CO**_**2**_ 47% O2 plus 53% air: 27.2 ± 1.2	**End-tidal pCO**_**2**_ 47% O2 plus 53% air: 18.3 ± 0.3	**p**_**a**_**O**_**2**_ 100% O_2_: 344.7 ± 6.8 1% CO_2_: 227.6 ± 6.0 2% CO_2_: 229.5 ± 4.9 5% CO_2_: 238 ± 5.5 95% O_2_: plus 5% CO_2_: 344.7 ± 6.8	**p**_**a**_**CO**_**2**_ 100% O_2_: 28.7 ± 3.0 1% CO_2_: 32.2 ± 1.1 2% CO_2_: 35.5 ± 1.6 5% CO_2_: 53.5 ± 2.2 95% O_2_: plus 5% CO_2_: 28.7 ± 3.0	**End-tidal pCO**_**2**_ 100% O_2_: 18.1 ± 0.6 1% CO_2_: 28.8 ± 0.7 2% CO_2_: 35.8 ± 1.3 5% CO_2_: 54.2 ± 1.3 95% O_2_: plus 5% CO_2_: 58 ± 2.2						Stimulation: Hyperoxia: yes Hypercapnia: yes Hyperoxia plus hypercapnia: yes Resting state: Hyperoxia: yes Hypercapnia: partial Hyperoxia plus hypercapnia: partial
Baseline BOLD signal	Prielmeier et al. (1993)	**Not reported** 30% O_2_ plus 70% N_2_O			**Not reported** 0% O_2_ (100% N_2_O)								Partial
Baseline BOLD signal	Prielmeier et al. (1994)	**Not reported** 30% O_2_ plus 70% N_2_			**Not reported** Not reported								Partial
Baseline BOLD signal; peripheral stimulation, electrical	Qiao et al. (2007)	**Blood pressure** Not reported			**Blood pressure** Noradrenaline 4–75 mmHg increase								Baseline: partial Stimulation: partial
Peripheral stimulation, electrical	Ramos-Cabrer et al. (2005)							**Transcutaneous pCO**_**2**_ Results shown in graphs as % changes relative to baseline transcutaneous pCO_2_ measurements; qualitatively 3 patterns identified					Yes
Peripheral stimulation, electrical	Schlegel et al. (2015)							**SpO**_**2**_**, heart rate, pulse distension**, Values shown only in graphs					Yes No changes in physiological parameters = more specific activation maps when randomised single pulse stimuli instead of block stimulation used. Under isoflurane as well as under medetomidine.
Central stimulation, phMRI	Schmidt et al. (2006)	**Respiratory rate** 81 ± 7	**Heart rate** 405 ± 49	**Blood pressure** 113 ± 14				**Respiratory rate** 103 ± 16 (sign. increase)		**Heart rate** 394 ± 39 (no sign. change)	**Blood pressure** 119 ± 15 (sign. increase)		No (all) Activations persist after return of RR, HR and BP to baseline.
Peripheral stimulation, electrical	Schroeter et al. (2014)	**SpO**_**2**_ Isoflurane: 95.8 ± 1.0–96.6 ± 0.5 Medetomidine: 94.1 ±1.4–96.7 ± 0.6 Propofol: 91.2 ± 2.2–97.2 ± 1.1 Urethane: 94.5 ± 0.5–95.9 ± 0.9	**Heart rate** Isoflurane: 502.1 ± 25.2–546.0 ± 17.5 Medetomidine: 331.3 ± 20.7–390.9 ± 27.4 Propofol: 517.1 ± 27.5 to 582.8 ± 111.6 Urethane: 626.3 ± 7.7–648.5 ± 16.0	**Pulse distension (μm)** Isoflurane: 12.7 ± 0.2–13.5 ± 0.1 Medetomidine: 16.2 ± 3.5–22.0 ± 9.7 Propofol: 23.7 ± 1.8–44.2 ± 10.1 Urethane: 10.1 ± 0.5–12.2 ± 0.5 to 10.1				**SpO**_**2**_ Isoflurane: 93.4 ± 1.1–96.6 ± 0.5 Medetomidine: 95.8 ± 1.0–96.8 ± 0.9 Propofol: 97.2 ± 0.5–97.8 ± 0.3 Urethane: 89.8 ± 0.2–95.9 ± 0.9		**Heart rate** Isoflurane: 502.1 ± 25.2–520.7 ± 12.2 Medetomidine: 331.3 ± 20.7–388.8 ± 80.8 Propofol: 517.1 v 27.5–609.8 ± 27.8 Urethane: 644.1 ± 11.6–658.3 ± 8.8	**Pulse distension (μ** **m)** Isoflurane: 12.7 ± 0.2–13.8 ± 2.1 Medetomidine: 13.7 ± 2.6–21.6 ± 7.1 Propofol: 37.5 ± 12.5–43.2 ± 10.4 Urethane: 8.7 ± 0.7–12.2 ± 0.5		Yes Values represent range of values measured in different time periods.
Peripheral stimulation, electrical	Schroeter et al. (2017)	**SpO**_**2**_ range at 1.1 to 1.5% isoflurane in different strains 94.73 ± 0.41–97.50 ± 0.36	**Heart rate** range at 1.1 to 1.5% isoflurane in different strains 517.73 ± 7.99–610.67 ± 9.67	**Pulse distension (μm)** range at 1.1 to 1.5% isoflurane in different strains 11.39 ± 1.42–19.22 ± 1.14				**SpO**_**2**_ Not reported		**Heart rate** Not reported	**Pulse distension (μ** **m)** Not reported		Yes Bilateral responses to unilateral hindpaw stimulation in acallosal mice
Baseline BOLD signal	Sedlacik et al. (2015)	**Not reported**			**Not reported** 100% O_2_ 10% CO_2_								Hyperoxia: yes Hypercapnia: yes
Baseline BOLD signal	Sicard et al. (2003)	**p**_**a**_**CO**_**2**_ Room air Awake: 36 ± 3 Isoflurane: 36 ± 2			**p**_**a**_**CO**_**2**_ 5% CO_2_ (21% O_2_) Awake: 47 ± 4 Isoflurane 50 ± 5 10% CO_2_ (21% O_2_) Awake: 65 ± 5 Isoflurane 69 ± 2								Partial
Baseline BOLD signal	(Sicard and Duong, 2005)	**SpO**_**2**_ 21% O_2_ plus 79% N_2_: 97 ± 0.4	**p**_**a**_**O**_**2**_ 21% O_2_ plus 79% N_2_: 88 ± 2	**p**_**a**_**CO**_**2**_ 21% O_2_ plus 79% N_2_: 37 ± 1	**SpO**_**2**_ 12% O_2_ (88% N_2_): 83 ± 3 9% O_2_ (91% N_2_): 75 ± 3 100% O_2_: 99 ± 0.0 5% CO_2_ (21% O_2_): 97 ± 0.4 10% CO_2_ (21% O_2_): 96 ± 0.4	**p**_**a**_**O**_**2**_ 12% O_2_ (88% N_2_): 40 ± 1 9% O_2_ (91% N_2_): 33 ± 2 100% O_2_: 324 ± 7 5% CO_2_ (21% O_2_): 102 ± 3 10% CO_2_ (21% O_2_): 112 ± 2	**p**_**a**_**CO**_**2**_ 12% O_2_ (88% N_2_): 32 ± 1 9% O_2_ (91% N_2_): 26 ± 1 100% O_2_: 40 ± 1 5% CO_2_ (21% O_2_): 49 ± 2 10% CO_2_ (21% O_2_): 70 ± 2						Hypoxia: yes Hyperoxia: yes Hypercapnia: yes
Peripheral stimulation, electrical	Sumiyoshi et al. (2012)							**p**_**a**_**O**_**2**_ 114 ± 16 (86–161)	**p**_**a**_**CO**_**2**_ 38.8 ± 7.3 (23.5–56.3)	**pH** 7.34 ± 0.04 (7.24–7.45)	**Heart rate** 467 ± 44 beats/min (376–571)	**Blood pressure** 105 ± 17 (66–141)	No (all parameters)
Baseline BOLD signal	Tsurugizawa et al. (2016)	**Blood pressure** 1.5% isoflurane: mean 79 ± 5						**Blood pressure** 2.0% isoflurane: mean 68 ± 7 2.5% isoflurane: mean 64 ± 3.0% isoflurane: mean 62 ± 6					No (monotonic blood pressure decrease with higher concentrations vs inverse U shape of BOLD signal)
Baseline BOLD signal; peripheral stimulation, chemical somatosensory	Tuor et al. (2002)	**Blood pressure** Mean 104 ± 10 (pre-treatment with morphine: 77 ± 20)			**Blood pressure** noradrenaline increase in mean from baseline 58 ± 23 (15–90)			**Blood pressure** Formalin injection in paw No pre-treatment: increase in mean from baseline 29 ± 14 (15–55) pre-treatment with morphine: increase in mean from baseline 46 ± 13					Baseline: yes Stimulation (i.e., effect of BP changes on response to formalin): partial
Baseline BOLD signal; peripheral stimulation, electrical	Tuor et al. (2007)	**Blood pressure** Stroke group: 98 ± 21 Control: 99 ± 18			**Blood pressure** Noradrenaline Not reported								Baseline: partial Stimulation: partial
Baseline BOLD signal	Vanhoutte et al. (2006)	**Body temperature** 37.0			**Body temperature** Warming and cooling 39.0–39.2 38.0								Yes
Baseline BOLD signal; peripheral stimulation, electrical	Wang et al. (2006)	**Blood pressure** Mean (?) 109 ± 19			**Blood pressure** Trimetaphan camsilate (vasodilator) decreases relative to baseline 1–10, 11–30, 31–45, 46–60, >60 Noradrenaline Increases relative to baseline baseline: 1–10, 11–30, 31–45, 46–60 an > 60								Baseline: Decrease: yes Increase: yes Stimulation Decrease: no Increase: yes
Baseline BOLD signal	Xu et al. (2000)	**p**_**a**_**O**_**2**_ spontaneous ventilation: 98.50 ± 2.86 controlled ventilation: 113.94 ± 5.92		**p**_**a**_**CO**_**2**_ spontaneous ventilation: 50.25 ± 1.86 controlled ventilation: 39.04 ± 3.21				**p**_**a**_**O**_**2**_ After heroine administration spontaneous ventilation: 70.93 ± 1.57 controlled ventilation: 106.68 ± 10.02		**p**_**a**_**CO**_**2**_ After heroine administration spontaneous ventilation: 67.58 ± 1.83 controlled ventilation: 39.44 ± 3.07			Yes (difference between modes of ventilation)
Baseline BOLD signal	Zaharchuk et al. (1999)	**Blood pressure** mean 130 ± 12			**Blood pressure** Blood withdrawal “mild hypotension”: 53 ± 3 “severe hypotension”: 26 ± 8								No

*References are sorted in alphabetical order. The parameters under investigation and the interventions to achieve those changes (interventional studies) or performed as part of the “normal” experiment during the observation period (observational studies) are indicated. The units used are: % for SpO_2_ and haematocrit; mmHg for p_a_O_2_, p_a_CO_2_, end-tidal pCO_2_ and arterial blood pressure; beats or breaths per minute for heart rate and respiratory rate; °C for temperature. Not reported = physiological parameter values and/or details of the intervention were not reported*.

Effects of changes in arterial partial pressure of O_2_ (p_a_O_2_) and/or p_a_CO_2_ were most commonly studied, followed by effects of arterial blood pressure variations. Interventional studies dominated and data was primarily available about effects on baseline BOLD signal and responses to peripheral stimulation.

**Figure 5:**
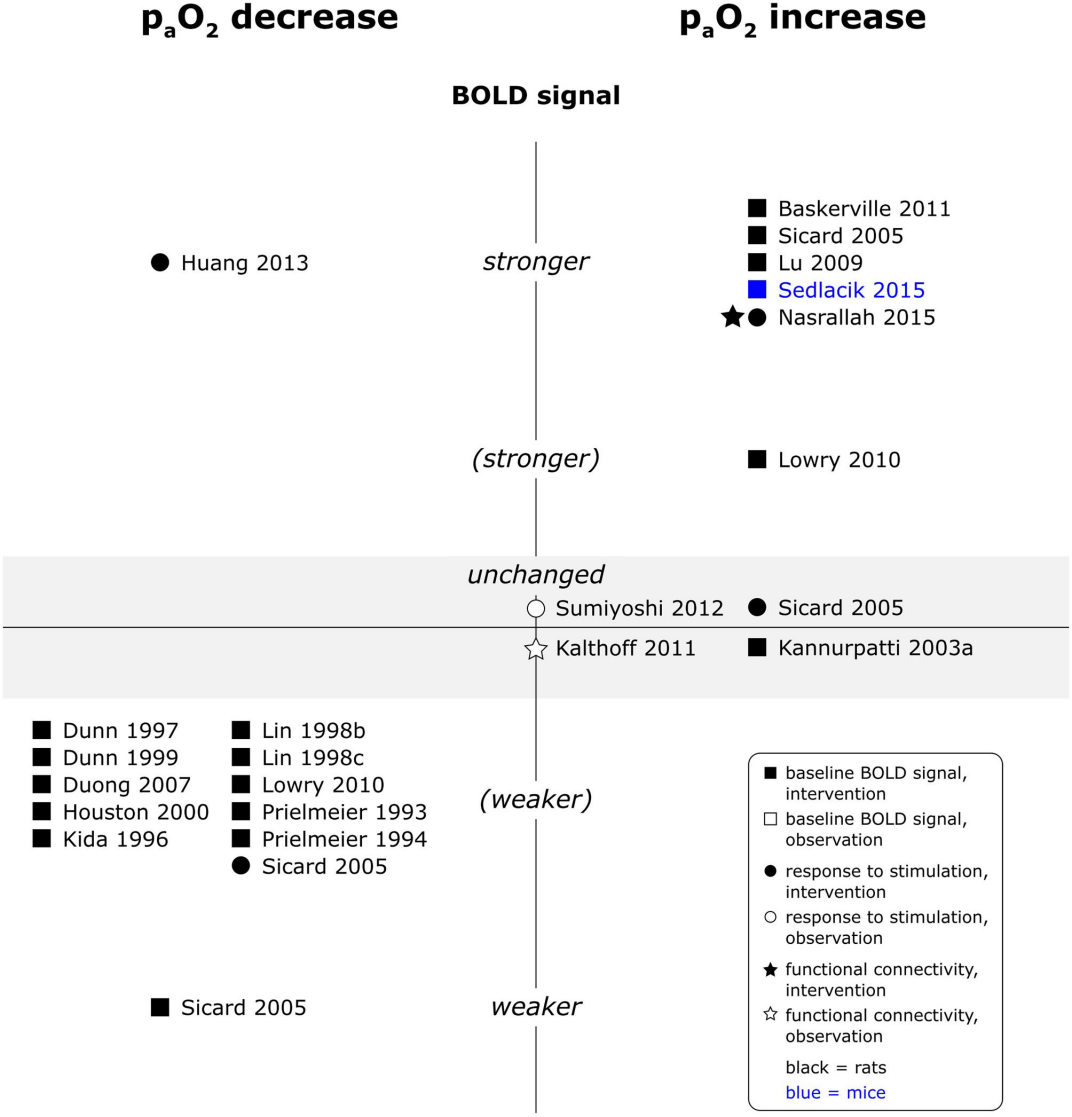
Effects of decreases and increases in p_a_O_2_ compared to respective baseline condition, on baseline BOLD signal, responses to stimulation and fc in rats and mice. Datapoints displayed on the BOLD signal axis represent studies which have investigated associations between p_a_O_2_ values and the respective BOLD outcome (in the absence of specific changes in p_a_O_2_). stronger = higher BOLD signal or lower R2*, higher signal intensity and/or spatial extent of activated area upon stimulation, or higher fc strength and/or spatial extent of connectivities; (stronger) = BOLD signal, response to stimulation or fc stronger in some, but not all aspects; unchanged = no significant difference to respective baseline condition or no association with p_a_O_2_ fluctuations found. “weaker” and “(weaker)” analogous to “stronger” and “(stronger).” One data point per experimental paradigm and per dataset. If no statement on the significance of reported changes was available, “(stronger)” or “(weaker)” were selected.

A reduction in p_a_O_2_ levels unequivocally reduced baseline BOLD signal intensity, whereas an increase in p_a_O_2_ generally increased baseline BOLD signal intensity (see Figure 5). Reported effects on responses to electrical paw stimulation were however less consistent and varied from no significant effect of p_a_O_2_ on responses in a multiple linear regression model (Sumiyoshi et al., 2012), over weaker responses under hypoxia, but unchanged responses under hyperoxia (Sicard and Duong, 2005), to one report each of stronger responses under hypoxia (Huang et al., 2013) and stronger responses under hyperoxia (Nasrallah et al., 2015). An observational study in mice additionally found a linear association between O_2_ delivery, calculated as arterial O_2_ saturation (SpO_2_) times CBV, and BOLD signal change during somatosensory stimulation (Schroeter et al., 2014). Functional connectivity (fc) maps, interhemispheric connectivity strength and fluctuation of the signal amplitude were larger or higher under experimentally induced hyperoxia in one study (Nasrallah et al., 2015), but another study did not find a significant number of voxels of which the signal time courses were correlated to naturally occurring fluctuations in SpO_2_ (Kalthoff et al., 2011). No data was available for central stimulation paradigms.

**Figure 6:**
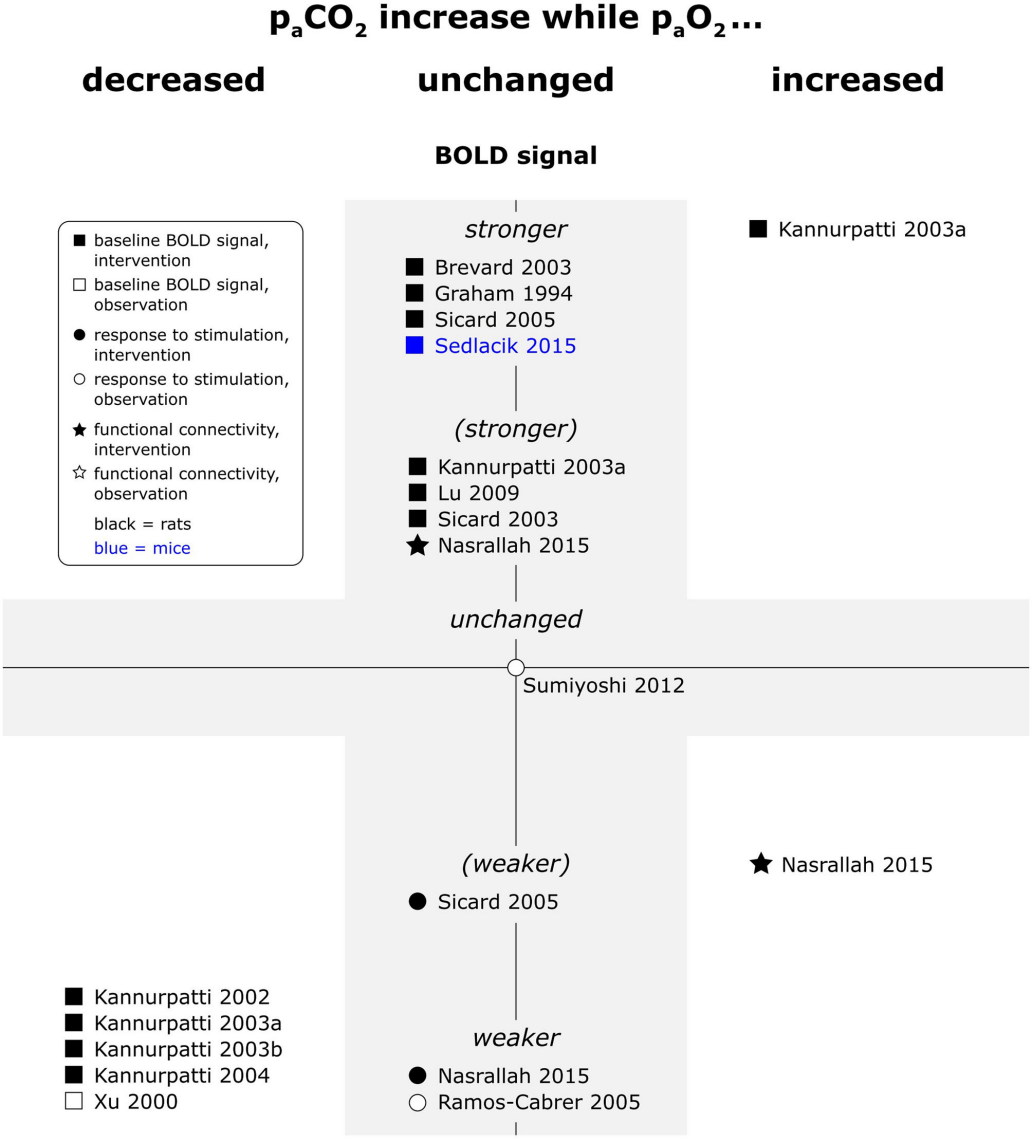
Effects of increases in p_a_CO_2_ compared to respective baseline condition, while p_a_O_2_ was decreased, unchanged or increased, on baseline BOLD signal, responses to stimulation and fc in rats and mice. Datapoints displayed on the BOLD signal axis represent studies which have investigated just associations between p_a_CO_2_ values and the respective BOLD outcome (in the absence of specific changes in p_a_CO_2_). stronger = higher BOLD signal or lower R2*, higher signal intensity and/or spatial extent of activated area upon stimulation, or higher fc strength and/or spatial extent of connectivities; (stronger) = BOLD signal, response to stimulation or fc stronger in some, but not all aspects; unchanged = no significant difference to respective baseline condition or no association with p_a_CO_2_ fluctuations found. “weaker” and “(weaker)” analogous to “stronger” and “(stronger).” One data point per experimental paradigm and per dataset. If no statement on the significance of reported changes was available, “(stronger)” or “(weaker)” were selected.

Hypercapnia consistently increased baseline BOLD signal and decreased response to peripheral stimulation (see Figure 6). In a single resting state study, fc maps were not affected by hypercapnia, but interhemispheric connectivity strength increased, and the frequency distribution of the interhemispheric correlation shifted (Nasrallah et al., 2015). At the opposite end of the spectrum, one study reported increased response to electrical paw stimulation in hypocapnic animals (Hsu et al., 1998). Another study did however not find a significant effect of naturally occurring fluctuations in p_a_CO_2_ on responses to electrical paw stimulation in a multiple linear regression model (Sumiyoshi et al., 2012).

In line with observations for hyperoxia and hypercapnia alone, combined hyperoxia and hypercapnia increased baseline BOLD signal in one study (Kannurpatti et al., 2003a). In contrast to both hyperoxia and hypercapnia alone, the spatial extent of fc maps was decreased, but the shift in frequency distribution of interhemispheric correlations occurred in the same frequency bands as with hypercapnia alone (Nasrallah et al., 2015). No data was available for effects of combined hyperoxia and hypercapnia on peripheral and central stimulation paradigms.

When hypercapnia was in contrast accompanied by reduced p_a_O_2_ levels, as with apnoea under room air, the effect of hypoxia dominated, and baseline BOLD signal intensity was reduced. A single study also reported reduced responses to central stimulation (phMRI) in hypoventilating animals (Xu et al., 2000).

Effects of hypercapnia as well as combined hypercapnia/hyperoxia were transient; 25 and 5 min after exposure, respectively, no persistent effects on peripheral electrical stimulation were detected (Bock et al., 1998; Dutka et al., 2002).

**Figure 7:**
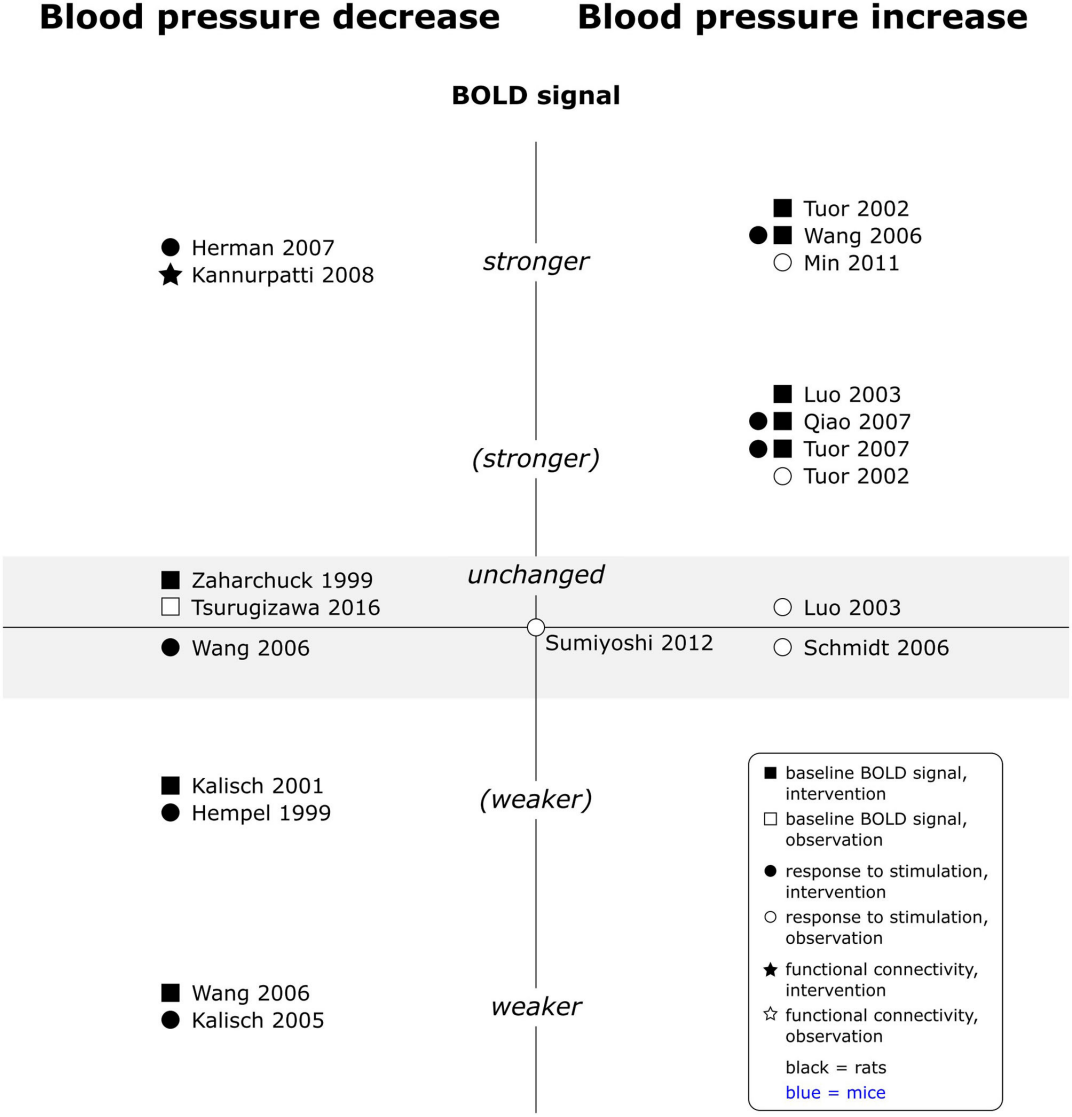
Effects of decreases and increases in arterial blood pressure compared to respective baseline, on baseline BOLD signal, responses to stimulation and fc in rats (no studies available in mice). Datapoints displayed on the BOLD signal axis represent studies which have investigated associations between arterial blood pressure values and the respective BOLD outcome (in the absence of specific changes in arterial blood pressure). stronger = higher BOLD signal or lower R2*, higher signal intensity and/or spatial extent of activated area upon stimulation, or higher fc strength and/or spatial extent of connectivities; (stronger) = BOLD signal, response to stimulation or fc stronger in some, but not all aspects; unchanged = no significant difference to respective baseline condition or no association with p_a_O_2_ fluctuations found. “weaker” and “(weaker)” analogous to “stronger” and “(stronger).” One data point per experimental paradigm and per dataset. If no statement on the significance of reported changes was available, “(stronger)” or “(weaker)” were selected.

Interestingly, findings for increases in blood pressure were overall consistent across experimental paradigms, whereas those for decreases were controversial within each paradigm (see Figure 7). Increases in blood pressure consistently increased the baseline BOLD signal and all responses to peripheral stimulation, including electrical somatosensory (Wang et al., 2006; Qiao et al., 2007; Tuor et al., 2007), chemical somatosensory (Tuor et al., 2002), and visceral stimulation (Min et al., 2011). Only two observational studies interpreted contributions of arterial blood pressure increases to phMRI responses as negligible (Luo et al., 2003; Schmidt et al., 2006). No data was available for effects on rsfMRI. Reported effects of blood pressure decreases on the other hand varied from no effect to decreased baseline BOLD signal and from reduced over unchanged to enhanced responses to peripheral stimulation. One phMRI study, where the test substance induced hypotension, found no specific response to the test substance but instead a positive correlation of arterial blood pressure time course and BOLD signal time course, unless arterial blood pressure values were stabilized with a vasopressor (Kalisch et al., 2005). Finally, a single rsfMRI study observed increased signal amplitude fluctuation and spatially more extended fc maps under lower arterial blood pressure. Several studies which experimentally modulated arterial blood pressure report dose dependent effects, and effects generally became statistically significant when arterial blood pressure changes exceeded 30 mmHg (Tuor et al., 2002, 2007; Wang et al., 2006; Qiao et al., 2007). A single study investigating naturally occurring blood pressure fluctuations (in the absence of interventions other than electrical paw stimulation) did however not find a significant effect of arterial blood pressure values on responses to stimulation in a multiple linear regression model (Sumiyoshi et al., 2012).

In mice, none of the included studies measured arterial blood pressure. Pulse oximetry indicates however that a cardiovascular response, evident as changes in heart rate, amplitude of the displayed pulse curve (called “pulse distension” by the authors) and SpO_2_ values, is elicited by somatosensory stimulation (Schroeter et al., 2014). Consecutive studies found that reduction of this cardiovascular response resulted in less activation in areas ipsilateral to the stimulated side (Schlegel et al., 2015), and that bilateral responses to unilateral stimulation were even present in acallosal animals (Schroeter et al., 2017), suggesting that cardiovascular responses contributed to the measured BOLD signal changes.

In rats, one observational study did not find a a significant effect of heart rate on responses to forepaw stimulation in a multiple linear regression model (Sumiyoshi et al., 2012), and another study observed that respiratory and cardiac waveforms and their derivates, rather than heart rate and respiratory rate, could reduce variance when used as regressors in rsfMRI (Kalthoff et al., 2011).

Finally, normovolaemic haemodilution as model for anaemia increased baseline BOLD signal intensity (Lin et al., 1998), whereas baseline BOLD signal intensity decreased when animals were experimentally warmed over 38°C (Vanhoutte et al., 2006).

Those references which studied the influence of physiological parameters under different anaesthetic protocols in rats suggest that responses to hypoxia, hypercapnia, apnoea, and decreases of blood pressure are qualitatively similar under different anaesthetics as well as under anaesthesia and in awake animals. Presence and direction of effects were consistent across groups. Significant quantitative differences were found in responses to apnoea under room air (Kannurpatti and Biswal, 2004), and alterations in inspiratory gas concentrations under urethane vs. pentobarbital and isoflurane vs. awake, respectively (Brevard et al., 2003; Sicard et al., 2003; Kannurpatti and Biswal, 2004; Duong, 2007). In mice, some differences in cardiovascular responses to stimulation were seen between different anaesthetics (Schroeter et al., 2014).

Interventional studies tended to more often find at least a partial effect than observational studies: while 4 out of 10 observational studies did not find an effect of one or more of the investigated parameters on the BOLD outcome measure, only 3 out of 33 interventional reported no effect of one or more of the investigated parameters, not counting the absence of effects beyond exposure to altered inspiratory gas mixtures. In two studies which combined interventional and observational aspects, reported effects were stronger in the interventional part (Tuor et al., 2002; Luo et al., 2003).

In summary, the majority of included studies reported at least partial effects of changes in physiological parameters on BOLD outcomes, although the direction of effects was sometimes controversial. Effects were observed across different types of fMRI, with a clear trend that interventional studies more commonly identified an effect than observational studies.

## Discussion

Here, we have systematically reviewed the effects of changes in physiological parameters during anaesthesia on BOLD fMRI readouts in rodents, based on studies directly manipulating specific parameters or analysing associations between changes occurring during the BOLD fMRI experiment and the measured outcomes.

The incidence of unphysiological and/or unstable physiological parameter values in rodents undergoing anaesthetised BOLD fMRI is unknown, as continuous monitoring of those parameters can be challenging and values are commonly reported as averaged values at specific timepoints. That said, general anaesthesia is well-known to affect physiological parameters, and the experimental protocol of BOLD fMRI studies often includes interventions which may on their own cause changes in physiological parameters. As the majority of included studies did report effects of changes in physiological parameters on BOLD outcomes, underlying mechanisms and possibilities to account for those potentially confounding factors will be discussed in the following sections.

### Changes in p_a_O_2_

Even when anaesthetic equipment is set up correctly and properly functioning, hypoxaemia is a common complication of anaesthesia. The combination of reduced lung volume (due to reduced respiratory muscle activity), atelectasis, ventilation-perfusion mismatch and hypercapnia results in reduced p_a_O_2_ (Lumb, 2019). With lower baseline arterial oxygenation, venous oxygenation decreases and so does BOLD signal intensity (Kim and Ogawa, 2012). Responses to stimulation are additionally modulated by effects of hypoxaemia on vessel tone and vascular reactivity. Reported responses to stimulation under hypoxaemia diverged, indicating that the net effect may depend on interactions between several factors. In those two studies which investigated responses to stimulation under hypoxaemia, animals were breathing spontaneously in one study (Sicard and Duong, 2005) and were ventilated in the other (Huang et al., 2013). Furthermore, SpO_2_ was reduced to lower levels in the first study (75% and 81%; FiO_2_ of 0.09 and 0.12) than in the second (87%; FiO_2_ 0.15).

Generic use of high inspiratory oxygen fractions (FiO_2_) to prevent hypoxaemia could however also result in confounding effects: elevated p_a_O_2_ reduces the amount of O_2_ dissociating from haemoglobin, because O_2_ dissolved in the plasma readily diffuses into surrounding tissues (Liu et al., 2019), resulting in increased baseline signal intensity. Furthermore, fc may be increased with hyperoxaemia, and potentially also response to stimulation (this was however not consistently found). In the included studies investigating effects of increased FiO_2_, baseline gas mixtures ranged from room air to 47% inspiratory oxygen. Most interventional studies used 100% O_2_ as hyperoxic condition, but one study found significantly increased baseline signal already at 40% O_2_ (Baskerville et al., 2011). Based on those findings, FiO_2_ in the range of 0.25 to 0.40 appears reasonable for BOLD fMRI, but further research is needed to identify optimal concentrations.

To reduce confounding effects, p_a_O_2_ should be monitored and controlled throughout image acquisition in all BOLD fMRI experiments.

### Changes in p_a_CO_2_

Direct depression of respiratory centres and peripheral muscle relaxation reduce minute alveolar ventilation in spontaneously breathing anaesthetised animals and lead to hypercapnia (Duke-Novakovski et al., 2016). Additionally, substances administered in an fMRI experiment may provoke hypo- or hyperventilation.

Locally increased pCO_2_ triggers vasodilation to increase perfusion of areas with increased metabolism (Shockley and LaManna, 1988). Accordingly, increases in systemic p_a_CO_2_ increase global CBF. The relation between p_a_CO_2_ and CBF is almost linear between 20 and 80 mmHg (Reivich, 1964). When cerebral perfusion is increased without concomitant increase of CMRO_2_, venous oxygenation increases and so does the baseline BOLD signal (Kim and Ogawa, 2012). Contrariwise, CBF response to stimulation is reduced when baseline CBF is already high (Cohen et al., 2002), explaining the reduced responses to stimulation. A recent study (published after the date the systematic search was performed) in mechanically ventilated vs. spontaneously breathing mice measured vessel diameter with optic intrinsic signal imaging as well as BOLD response to electrical paw stimulation. At the average p_a_CO_2_ of 83 mmHg in spontaneously breathing animals, baseline vessel diameter was significantly larger, and increase in vessel diameter as well as the peak BOLD response to paw stimulation were significantly reduced and delayed compared to values measured at an average p_a_CO_2_ of 41 in mechanically ventilated animals (Shim et al., 2020). One of the included interventional studies however observed a significant decrease of response to stimulation already with 1% CO_2_ admixture, corresponding to an increase in p_a_CO_2_ of 5 mmHg (Nasrallah et al., 2015). In humans, fluctuations in EtCO_2_ as small as 1.1 mmHg were reported to correlate with fluctuations in global BOLD signal intensity (Wise et al., 2004).

By which mechanisms and to which extent hypercapnia affects fc is less clear. A single study found increased interhemispheric S1FL connectivity and increased amplitude of signal fluctuations under one of several tested CO_2_ concentrations, however, spatial extent of fc maps was unchanged and there was no correlation between p_a_CO_2_ and amplitude of signal fluctuations or between responses to stimulation and fc, so that the authors question the cardiovascular origin of the observed changes (Nasrallah et al., 2015). In humans, hypercapnia was shown to affect interhemispheric connectivity between homotopic regions (Marshall et al., 2015). Until proven otherwise, effects of increases of p_a_CO_2_ on rsfMRI measures should be expected.

At the other end of the spectrum, hypocapnia provokes strong vasoconstriction and reduction of baseline CBF (Severinghaus and Lassen, 1967). Although so far only effects on response to forepaw stimulation have been investigated in rodents (Hsu et al., 1998), effects on other types of fMRI may be expected as well.

Data included in this review about the effects of p_a_CO_2_ and p_a_O_2_ alterations on BOLD result are almost exclusively from rats, as only one study investigated different gas compositions in mice. Results of that publication are however in line with rat data, and supported by more recent findings in mice, suggesting similar effects in both species. Consequently, p_a_O_2_ and p_a_CO_2_ need to be monitored and controlled throughout image acquisition in all BOLD fMRI experiments.

### Changes in Arterial Blood Pressure, and Cardiovascular Responses in General

Two factors can cause blood pressure changes during BOLD fMRI: First, hypotension is a common complication of anaesthesia, defined as mean arterial blood pressure (MAP) below 60 mmHg or systolic arterial blood pressure below 80 mmHg (Duke-Novakovski et al., 2016). Second, stimuli applied during fMRI can provoke concomitant arterial blood pressure increase or, less commonly, decrease. Many substances tested in phMRI change arterial blood pressure for variable durations. Additionally, noxious or intense peripheral stimuli, such as application of irritating substances, can increase arterial blood pressure. For example, arterial blood pressure increases and BOLD signal increases are significantly linearly correlated after formalin injection into the paw (Tuor et al., 2002). Especially abrupt blood pressure changes could override cerebral autoregulation for a short duration and introduce artefactual—positive or negative—changes in fMRI signal.

While arterial blood pressure increases are consistently reported to increase baseline BOLD signal and response to peripheral stimulation, decreases are inconsistently reported to decrease baseline BOLD signal and their effect on response to stimulation is controversial (from decreased over unchanged to enhanced responses).

Blood pressure increases were in four out of five interventional studies produced by noradrenaline boli applied over maximally 1 min. Noradrenaline is generally assumed not to affect cerebral vasculature due to the blood brain barrier (Hardebo and Owman, 1980). Accordingly, MAP and thereby cerebral perfusion pressure should increase without affecting autoregulation. In principle, cerebral autoregulation should maintain CBF at constant levels for MAP of 50 to 140 mmHg by adjusting cerebral vascular resistance (Zaharchuk et al., 1999). To which degree autoregulation is preserved under anaesthesia depends however on the agent used (Wang et al., 2010). For the included studies it is often difficult to reconstruct whether the induced blood pressure increases were within or beyond the autoregulatory limits reported by Zaharchuk et al. (1999); for example, two studies did not report baseline blood pressure (Tuor et al., 2002; Qiao et al., 2007). It appears that while some BP increases exceeded 140 mmHg MAP (Wang et al., 2006), others were mainly within autoregulatory limits (Tuor et al., 2007). Irrespective of the blood pressure increases produced, the included studies generally ascribe the increase in baseline BOLD signal to hyperperfusion of the brain. In such a state of high perfusion pressure, increased responses to stimulation are plausible, as stimulation-induced dilation of arterioles will lead to a strong CBF response and a clear “surplus” of O_2_ delivery.

Blood pressure decreases, on the other hand, were induced by more diverse methods. Several studies used blood withdrawal, at 1 to 10 ml/kg per step and at various rates. One study performed several steps of blood withdrawal and subsequent re-infusion (Kalisch et al., 2001). Although studies took care to avoid haemorrhagic shock, the higher volumes of 8–10 ml/kg may have activated compensatory mechanisms including release of vasoactive substances (Kalisch et al., 2001). Other studies used negative lower body pressure (Herman et al., 2007) or trimetaphan camsilate (Wang et al., 2006) to decrease arterial blood pressure. Net effects on cerebral haemodynamics may therefore have varied.

Blood pressure decrease studies used also more diverse anaesthetics. However, one study found significant correlations between blood pressure and BOLD signal under isoflurane as well as under halothane and propofol; and three studies observing divergent effects on responses to stimulation were all performed under α-chloralose anaesthesia (Hempel et al., 1999; Wang et al., 2006; Herman et al., 2007). It appears therefore unlikely that solely the anaesthetics used explain inconsistent findings.

Decreases within and below autoregulatory limits were usually observed within the same study. Some studies report more pronounced effects on baseline signal with larger blood pressure decreases (Wang et al., 2006) or that response to stimulation was only reduced when blood pressure fell below the autoregulatory limit (Hempel et al., 1999), but results usually refer to the total range of observed blood pressure decreases.

Assuming that cerebral autoregulation was active, a decrease in cerebral perfusion pressure increases CBV, which may, under certain circumstances, account for a higher total amount of deoxyhaemoglobin and accordingly lower baseline signal intensity (Liu et al., 2019). When blood pressure was reduced below autoregulatory limits or in cases in which autoregulation may have been impaired, reduced cerebral perfusion readily explains the decrease in baseline BOLD signal: cerebral hypoperfusion increases O_2_ extraction fraction and thus decreases venous oxygenation, which translates into lower BOLD signal intensity.

In a situation of cerebral hypoperfusion, one would expect that the CBF response to stimulation is reduced, first because arterioles are at baseline already rather dilated and second because cerebral perfusion pressure is low enough that an additional decrease in vascular resistance will only return a small increase in CBF. A smaller increase in additional O_2_ delivery and thus a smaller BOLD signal increase would consequently be expected. However, BOLD signal depends on CBF, CBV and CMRO_2_, and the changes in CBV during hypotension are as controversial as the findings of stimulation studies [discussed by Zaharchuk et al. (1999)]. Further studies are required to characterise how systemic hypotension modulates BOLD responses to stimulation. Interestingly, Kannurpatti et al. (2008) found that although mean CBF is maintained when MAP approaches the autoregulatory limit, CBF fluctuations increased and so did BOLD signal fluctuations.

In mice, effects of blood pressure changes have not been investigated, but stimulation-associated cardiovascular responses are suspected to account for bilateral responses to unilateral electrical paw stimulation (Schroeter et al., 2014, 2017; Schlegel et al., 2015). Cardiovascular responses were however characterised only by pulse oximetry (heart rate and pulse distension) and arterial blood pressure was not measured in those studies. Interestingly, cardiovascular changes were not detected when randomised single pulses were applied and activation of ipsilateral cortex was reduced, although not abolished. As bilateral responses to unilateral stimulation were even observed in acallosal mice, a cardiovascular or at least unspecific neural arousal response seem to best explain those unspecific activations.

A study in rats did not find a significant effect of heart rate or MAP values on the BOLD response to electrical paw stimulation, but this study analysed one value (per parameter) per experiment, i.e., short term fluctuations were not addressed (Sumiyoshi et al., 2012).

Overall, despite open questions about the underlying mechanisms and the direction of effects, arterial blood pressure changes have to be expected to modulate the BOLD signal and may introduce artefactual activations or correlations when not detected and corrected for. Blood pressure should therefore be measured during fMRI. Counteraction of test-substance-induced hypotension in phMRI with a phenylephrine-CRI has been reported, but it remains to be determined whether pharmacological blood pressure stabilisation or prevention of cardiovascular responses in general enhances specificity of responses.

### General Considerations

Although discussed separately so far, physiological parameters should not be considered as isolated entities: changes in one parameter will likely trigger changes in others. For example, spontaneously breathing animals exposed to hypercapnic or hypoxic inspiratory gas mixtures will increase minute ventilation in an attempt to normalise p_a_CO_2_ or p_a_O_2_, and consequently partial pressures of both gases change. Similarly, during apnoea or severe hypoventilation, p_a_CO_2_ increases while p_a_O_2_ decreases. But not only respiratory aspects are interrelated. Mild hypercapnia or hypoxaemia increases arterial blood pressure and heart rate via sympathetic stimulation (Kuznetsova and Kulikov, 2014; Prabhakar et al., 2015), whereas severe hypoxaemia decreases arterial blood pressure (Kannurpatti and Biswal, 2004; Sicard and Duong, 2005). Furthermore, body temperature modulates both cardiovascular parameters and metabolism, which in turn affects O_2_ consumption and CO_2_ production (Kurz et al., 1996). This interdependence of physiological parameters may explain some of the variability of the results.

Partially due to this interdependence of physiological parameters, partially due to the quality of the included data, pointing out a single parameter which exerts the largest influence on the BOLD signal is not possible with the data presented here. In a significant proportion of studies, physiological parameter measurements were not reported or not reported in a way that would allow meaningful analysis. For example, blood pressure measurements were in several studies presented as a range of baseline values and the range of changes, but it was not clear whether values of individual animals were within or beyond autoregulatory limits (see Table 6). Additionally, the temporal scale differs between cardiovascular parameters (heart rate, blood pressure), which may change rapidly (e.g., in response to stimulation) or fluctuate over longer periods, and blood gas levels, which typically change over several tenths of seconds to even minutes. Consequently, the temporal resolution of data collection may influence the likelihood that an existing effect is detected.

For further studies, it would be interesting to investigate cardiovascular parameters, blood gas levels and body temperature concurrently with a high temporal resolution, and determine the magnitude of effect of parameter changes both in relation to what is considered a physiological range and to which changes are expected to occur within an experiment. Importantly, such investigation should be performed under different commonly used anaesthetics, as α_2_-agonists for example are strong vasoconstrictors, whereas inhalant anaesthetics are strong vasodilators, which may mediate the influence a given change in physiological parameters has on the BOLD signal.

In the studies included in this review, a range of anaesthetics was used, which may explain some of the variability of the results. A few of the included studies compared effects of physiological parameter alterations on BOLD fMRI results under different anaesthetics and found qualitatively similar effects, but also noted some quantitative differences.

Finally, interventional studies more commonly reported an influence of physiological parameters on the BOLD signal than observational studies. A possible explanation is that induced changes of physiological parameters were larger than naturally occurring changes. Alternatively, other physiological parameters may have been better controlled for in interventional studies.

In summary, the available data indicate that BOLD fMRI measurements risk to be confounded by unstable physiology. It is questionable whether results from studies with unstable and/or unphysiological values of physiological parameters can be interpreted, as effects are not well-enough characterised to be simply “subtracted.” But without appropriate monitoring, such limitations cannot be detected, and accordingly, results can neither be reliably interpreted nor compared between studies. Appropriate monitoring and anaesthetic management are therefore crucial to the scientific validity of studies—which, in turn, is a prerequisite for studies to create any benefit, which justifies the use of animals.

### Practical Implications for Monitoring

Based on the findings of this review, fMRI studies should monitor blood oxygenation, ventilation and cardiovascular parameters. As part of good anaesthetic practice, reflexes and body temperature should also be monitored.

Only a minority of the included studies investigated mice (14 out of 121 references), which may be related to difficulties maintaining the animals in stable conditions throughout experiments. In the following sections, practical challenges of appropriate monitoring in mice as opposed to rats will be highlighted.

#### Blood Oxygenation and Ventilation

There are different options to monitor ventilation and blood oxygenation. Respiratory rate is commonly measured with MRI-compatible sensors. For anaesthetic safety, respiratory rate is an important parameter to monitor, because apnoea or severe respiratory depression can be fatal. In spontaneously breathing animals, respiratory rate additionally helps to estimate anaesthetic depth, with higher than expected rates typically indicating superficial levels of anaesthesia and lower than expected rates indicating (too) deep levels of anaesthesia, although other reasons for changes in both directions are possible and values as well as changes of respiratory rate need to be interpreted in context (a complete guide on how to interpret each physiological parameter in relation to all others is beyond the scope of this review). Although providing useful information, monitoring of respiratory rate alone is insufficient as it does not provide any information about blood oxygenation nor levels of CO_2_.

The most accurate way to assess ventilation and blood oxygenation is direct measurement of p_a_CO_2_ and p_a_O_2_ in arterial blood. However, this is invasive and the number of samples which can be taken before blood loss becomes substantial is limited. Approximately 0.1 ml of blood are required per analysis with standard blood gas analyzers [see instructions for a machine in use at the Institute for Physiology of the University of Zurich (Institute for Physiology UZH Zurich integrative rodent physiology (ZIRP), 2016)]^2^, and maximally 10% of total blood volume should be sampled, which corresponds to 2.56 and 0.14ml in a rat of 400 g and a mouse of 25 g, as outlined by the National Centre for the Replacement Refinement and Reduction of Animals in Research (n.d.-a, n.d.-b)^3^^,^^4^. In mice, multiple samplings are therefore basically excluded. Substantial blood loss, i.e., if more than 10% of the blood volume would be sampled, not only imposes physiological stress on the animal, but potentially affects the BOLD signal by inducing hypotension, or haemodilution if the withdrawn volume is replaced. But even in rats, where multiple sampling is feasible, the intermittent measurements may not provide a sufficient temporal resolution to detect changes occurring during scans. Thus, while arterial blood gas analysis provides the most accurate assessment of oxygenation and ventilation, additional continuous monitoring techniques would enhance detection of short-term fluctuations.

A continuous and non-invasive option for monitoring blood oxygenation is pulse oximetry. Pulse oximetres provide real-time monitoring of arterial haemoglobin O_2_ saturation and typically display heart rate along with SpO_2_. MRI-compatible devices are available and easy to use. Pulse oximetry is a powerful tool to ensure that animals are not hypoxaemic. Due to the sigmoid relation between p_a_O_2_ and SpO_2_, it is however not able to differentiate between normal p_a_O_2_ observed at room air (around 100 mmHg) and increased p_a_O_2_ under hyperoxic conditions (up to 500 mmHg) (Duke-Novakovski et al., 2016). Grading of moderate levels of hypoxaemia (p_a_O_2_ 60–90 mmHg) by pulse oximetry is also not very accurate, as those values correspond to SpO_2_ of ~88 to 95% (Cartheuser, 1993).

As discussed earlier, optimal FiO_2_ for BOLD fMRI in anaesthetised animals is likely in the range of 0.25 to 0.40. The inspired oxygen concentration provided should be continuously measured with a relevant gas analyser, that is calibrated at regular intervals predetermined by manufacturers.

Instead of monitoring blood oxygenation and ventilation in animals undergoing fMRI, some studies attempt to control those parameters by mechanically ventilating all animals with settings determined in pilot bench-top experiments. While this approach may on average keep p_a_CO_2_ and p_a_O_2_ within reasonable limits, it first requires that the ideal parameters were determined under identical conditions as in the experiment, and second has the major limitation that eventual outliers are not detected. Notably when low animal numbers are used, individual outliers may have a relevant impact on the results if undetected (which is a general problem of measuring physiological parameters in a bench-top group only). If the pre-set ventilatory parameters do not exactly match individual requirements, hyper- or hypoventilation can develop over time. Monitoring of CO_2_ in particular allows ventilatory settings to be adjusted before significant changes in p_a_CO_2_ develop, and thus helps to keep p_a_CO_2_ within a narrow range during the experiment.

As an estimate for p_a_CO_2_, end-tidal CO_2_ can be measured by capnometry or -graphy. A sensor for transcutaneous blood pCO_2_ measurement was tested in one of the included studies, but considerable inter-individual differences in correlation with p_a_CO_2_, dependence of measurements on skin perfusion, and skin lesions caused by the high temperature of the sensor were reported (Ramos-Cabrer et al., 2005). Therefore, we currently recommend measuring end-tidal rather than transcutaneous pCO_2_.

With capnometry, it is crucial that devices are calibrated against p_a_CO_2_, as significant discrepancies between arterial and measured end-tidal pCO_2_ can result from the rodents' small tidal volume, documented for example in Nasrallah et al. (2015). Due to the low tidal volumes and high respiratory rates, capnometers specifically designed for rodents should be used (Beck et al., 2014). Capnometers can be attached to face masks or nose cones (Silva et al., 2011), but more accurate measurements are obtained when animals are intubated. Intubation also enables mechanical ventilation, which is generally recommended for BOLD fMRI to keep p_a_CO_2_ within a narrow physiological range (The high resistance of the tubes probably even makes mechanical ventilation mandatory in intubated rodents).

Intubation can be performed via tracheostomy or the oropharyngeal route. While both routes may be used in terminal studies, the less invasive oropharyngeal route should be chosen for recovery studies. While rats appear to recover with few complications from oropharyngeal intubation (Rivard et al., 2006), a recent publication described cessation of weight gain in the first week after extubation in mice (Shim et al., 2020). One of the authors (AS) has however successfully recovered mice after intubation and mechanical ventilation, suggesting that longitudinal studies with appropriate ventilatory management are feasible in mice, although the technical challenges may require an experienced operator.

Apart from potential complications with intubation, mechanical ventilation requires sufficient depth of anaesthesia and adequate ventilator settings to prevent that animals are “fighting” the ventilator [with potentially fatal consequences, see Ramos-Cabrer et al. (2005)]. If more superficial anaesthesia, for example a medetomidine-CRI, is desired for an experiment, paralysing animals may be required to enable mechanical ventilation and also to prevent reflex responses to stimulation, which could create massive motion artefacts. The use of neuromuscular blocking agents poses practical challenges: in a clinical setting the degree of neuromuscular blockade is monitored to avoid residual paralysis in recovery, for example by train of four electrical stimulation (Duke-Novakovski et al., 2016). In rodents however, such clinically used equipment is not applicable, because animals are too small. To the best of our knowledge, only invasive techniques of neuromuscular blockade assessment are described (Itoh et al., 2000), which are not feasible for routine use. Consequently, animals in recovery studies are at risk for respiratory complications from residual neuromuscular block during recovery when paralysed for the experiment.

#### Cardiovascular Monitoring

As shown by the effects of blood pressure changes on the BOLD signal, blood pressure needs to be measured in anaesthetised animals during fMRI to obtain valid results. To date, blood pressure is in both species typically measured invasively; none of the included studies used non-invasive devices. This means that appropriate monitoring during the experiment is more invasive than then actual experiment, which somewhat offsets the advantage of fMRI being a non-invasive technique.

While non-invasive blood pressure measurement is feasible in rats, and MRI-compatible devices are marketed, it is challenging in mice due to their small size. Once non-invasive devices are validated and accessible, non-invasive blood pressure monitoring during fMRI should be attainable in rats and broadly implemented. For mice however, invasive blood pressure measurement may remain the standard, a standard which is technically demanding and not practicable for longitudinal studies, so that the current trade-off between quality of anaesthetic monitoring and invasiveness of the experiment likely persists.

Although heart rate was not shown to directly correlate with BOLD signal, it should be monitored during fMRI, as changes in heart rate can indicate responses to stimulation, changes in anaesthetic depth or also changes in p_a_CO_2_ and p_a_O_2_. Heart rate, similar to respiratory rate, is a sensitive, but not specific parameter and therefore needs to be interpreted in the context of other physiological parameters, experimental stimulation and the anaesthetic protocol used. A convenient method to measure heart rate is by pulse oximetry. Alternatively, MRI-compatible ECG electrodes can be used (Choquet et al., 2011).

Some pulse oximeters display a pulse curve. Schroeter et al. (2014) used changes in the waveform amplitude as an indicator for changes of blood pressure. However, this specific measure was not validated and should be interpreted with caution. Generally, variation in the waveform amplitude measured by the pulse oximeter results from an interplay between vascular resistance and stroke volume (Cannesson et al., 2005). Assuming constant vascular resistance over a short period of time (in the range of a few seconds), relative changes in the waveform amplitude indicate a change in stroke volume. This may be sufficient to detect cardiovascular arousal as intended in that study. Inferences on—absolute or relative–arterial blood pressure changes are however not warranted (Dorlas and Nijboer, 1985) and pulse oximetry can therefore not replace blood pressure monitoring.

#### Additional Considerations

In terms of good anaesthetic practice, it is furthermore advisable to monitor reflexes and temperature.

Reflexes give a rough clinical estimate of the depth of anaesthesia. Before an animal is fixed on the animal holder, righting and limb- or tail-withdrawal reflex should be tested, especially if agents were administered intraperitoneally, as this route is associated with high failure rates of around 20% (Miner et al., 1969; Zatroch et al., 2017), so that the same levels of anaesthesia will not be reached in all animals. The desired depth of anaesthesia may vary depending on the protocol used, but at least righting reflex should be lost under exposure to scanner noise. In dogs, 0.8 to 1.2 MAC of sevoflurane are required to immobilise and mechanically ventilate the animals (Beckmann et al., 2020). Reflexes should be checked repetitively and at the end of scan to detect lightening of anaesthesia which may have occurred during image acquisition, especially if injection anaesthesia is used.

Body temperatures typically ranged from 35.5 to 37.5°C in the included studies, and no data was available on temperature-dependent effect on the BOLD signal in this range. As cerebral metabolism is reduced (Busto et al., 1987) and the O_2_-binding curve of haemoglobin shifted to the left (Armstrong et al., 2005) at lower body temperatures, higher blood oxygenation and accordingly baseline BOLD signal intensity would be expected. In addition, vasoconstriction at lower body temperatures (Armstrong et al., 2005) may increase the signal change upon activation, as more “capacity” for vasodilation exists, but the true effects remain to be investigated. Regardless of effects on the BOLD signal, hypothermia has detrimental physiological effects such as bradycardia and hypotension, prolongs recovery, and reduces anaesthetic requirements (Armstrong et al., 2005). Rats and mice are prone to hypothermia under anaesthesia due to their small body size. To prevent complications, the decrease in body temperature should be minimised by warming the animal, for example with feed-back controlled MRI-compatible warming mats, and body temperature monitored (Flecknell, 2015). Special care should be taken when stroke models are imaged, as spontaneous hyperthermia can occur in those animals (Zaremba, 2004), which both has consequences for the animal's physiology and has been shown to decrease BOLD signal intensity (Vanhoutte et al., 2006).

#### Summary

Taken together, appropriate monitoring for a BOLD fMRI experiment is continuous, invasive and not simple. To control p_a_CO_2_, probably the most important factor, either blood samples have to be drawn or the animals have to be intubated to allow accurate capnography. Mechanical ventilation is generally recommended to keep p_a_CO_2_ constant and should be used together with monitoring of end-tidal CO_2_. Mechanical ventilation of rodents via a nose-mask is described, but in 12% of cases the lung was not successfully ventilated with this option (Rindfield and McBrian, 2012). If refinement of this method proves feasible in further studies, mechanical ventilation via nose-mask could provide a less invasive and technically less demanding alternative to intubation, and enable appropriate ventilatory monitoring in longitudinal studies.

For blood pressure monitoring to be more accessible and generally applied, non-invasive devices should be validated and/or developed for rodent MRI. Reliable non-invasive blood pressure monitoring would allow BOLD fMRI studies to be non-invasive but at the same time sensitive enough to recognise any potentially relevant blood pressure changes during the experiment.

Overall, with the currently available techniques, appropriate anaesthetic monitoring and management is easier to implement in rats than in mice. Imaging rats rather than mice may therefore reduce the risk of unstable physiology confounding BOLD fMRI results.

### Limitations

This systematic review was limited by the fact that only in a quarter of the included references data was extracted in duplicate. Despite good agreement in those references, some mistakes or misinterpretations made during data extraction may have gone undetected in the rest of the references, which could have been avoided by consequent double extraction. An even more important limitation is however that only one reviewer was available for study selection. Subjectively unclear references were discussed with a supervisor and all borderline cases documented, but this approach does not provide the same degree of accuracy as study selection by two independent reviewers. Additionally, in- and exclusion criteria had to be further specified during study selection because the initial version did not cover all the situations encountered. Especially for clarification of the exact inclusion and exclusion criteria, discussion with a second reviewer would have supported objectivity and consistency of classifications.

On the side of included references, a major limitation of this review is that all included publications scored as having a high risk of bias. Accordingly, the strength of evidence is limited for any of the reported findings. The primary reason why studies were classified as having a high risk of bias was lack of blinding during the experiment and/or for data analysis. Lack of blinding during the experiment is common in basic research (van der Worp et al., 2005; Hooijmans et al., 2014; Macleod et al., 2015; Vogt et al., 2016). Often the same person is responsible for planning and performing the experiment and later analysing data. But even if it is not feasible for the responsible investigator to be blinded during the experiment due to lack of personnel or resources, and despite a standardised pipeline of analysis, fMRI data could be analysed in a randomised, blinded way (Hooijmans et al., 2014), so that no room for bias is left if the analysis pipeline requires some fine-tuning. Apart from blinding, in a substantial percentage of publications concerns associated with study design were present. Furthermore, reporting of measures against internal bias was—in line with published findings (Macleod et al., 2015; Rufiange et al., 2019)–generally low, despite introduction of the ARRIVE guidelines in 2010 (Kilkenny et al., 2010), leaving the question whether measures were not taken or “just” not reported.

Risk of bias being high in all studies means that strength of evidence is overall weak, and it is possible that future research will complement or correct our current understanding of how physiological parameters and states of anaesthesia (see article b) influence BOLD fMRI in rodents (Higgins et al., 2011; de Vries et al., 2015). A uniform level of risk of bias means further that consistency of findings for a certain factor is the only means to grade the strength of evidence between findings within this review.

Besides concerns about general methodologic quality of the included studies, examples for potentially confounding factors addressed by this review were observed in some of the included studies. For example, Nasrallah et al. (2015) compared responses to paw stimulation and fc under different inspiratory gas compositions, including several concentrations of CO_2_, to a “normal” condition in which animals were ventilated to a p_a_CO_2_ of 27 mmHg, which is clearly below reference ranges (Brun-Pascaud et al., 1982).

Furthermore, it is well-known that stimulus-evoked BOLD responses depend on the pulse sequence and spatial resolution used for image acquisition. Given the hemodynamic nature of the BOLD response, it is obvious that data acquisition procedures also affect the sensitivity towards changes in systemic physiological parameter. Most of the references included in this part of the review (42 out of 49) used gradient echo (GE) pulse sequences. In general, GE is considered more sensitive than spin echo (SE) in detecting activation-induced BOLD signal changes (Han et al., 2019). Although this increased sensitivity also translates into enhanced susceptibility towards changes in general physiological parameters, all SE studies included in this review reported at least partial effects of changes in p_a_O_2_, p_a_CO_2_ or blood pressure on the BOLD signal. Hence, the data included in this review suggests that effects of physiological parameter changes on the BOLD signal are present both in studies using GE and SE.

Low spatial resolution means enhanced likelihood that a large vessel might influence the signal (in adjacent voxels). Only for 6 out of 49 studies included in the analysis voxel dimensions were 200 ×200 μm or better. Among those 6 high resolution studies however, all four interventional studies reported effects of ventilatory or cardiovascular parameters on the BOLD signal, while only the two observational studies did not find clear effects, similar to the overall pattern across all included references. This indicates that studies using high spatial resolution are not exempt from confounding effects of physiological parameter changes on the BOLD signal.

Despite the potential of pulse sequences, spatial resolution, methods of analysis, anaesthetic agents and other aspects of experimental design to confound the findings of individual studies, we decided against a “study quality” assessment that goes beyond the established risk of bias assessment. Such a “study quality” score would have been subjective and specifically for anaesthetic agents a circular argument. A multivariate analysis whether each of those factors had an influence on whether studies did find an effect of physiological parameter changes on the BOLD signal would have been hampered by the semi-quantitative nature of our results and was therefore not performed. Instead, we decided to see whether effects of physiological parameters on BOLD results could be identified despite all heterogeneity and potential confounders within the included studies.

The included studies exhibited considerable heterogeneity regarding study design, experimental procedures (e.g., whether surgery was performed prior to imaging, ventilatory management), and technical details. Additionally, as no limitations were defined for the outcome measures except that it had to be directly derived from the BOLD signal, a variety of outcome measures was encountered in the included studies. The diversity of types of fMRI (baseline signal vs. stimulation vs. resting state) and outcome measures, together with the diversity of physiological parameters investigated, resulted in generally few reports per comparison and many unique reports. On one hand, this diversity narrowed the number of comparisons for which enough data was found to arrive at a conclusive summary. Specific observations were often complementary rather than comparable between studies. On the other hand, the fact that despite heterogenous experimental conditions and designs effects of p_a_O_2_, p_a_CO_2_ and blood pressure were consistently reported, strengthens the evidence that physiological parameter effects on BOLD fMRI results are real and relatively robust across conditions.

### Implications for Validity of BOLD fMRI in Rodents

The effects of anaesthesia-related changes in physiologic parameters on BOLD fMRI results relate to several aspects of validity.

The fundamental question whether imaging anaesthetised animals allows to adequately model complex behavioural, cognitive or emotional functions of the human brain cannot be generally answered. It depends on the specific model, anaesthetic protocol and management whether construct validity is given, and a detailed assessment of construct validity of the variety of models in use is beyond the scope of this review. Importantly, if construct validity is not given, an experiment cannot be justified. In some cases, the difference between impaired internal and construct validity may be gradual: do unspecific BOLD signal changes due to cardiovascular arousal in mouse stimulation studies for example “merely” introduce bias or prevent any meaningful conclusions? In this specific example, performing the experiment in rats instead of mice may increase validity, because unspecific signal changes in response to peripheral stimulation appear to be less common in rats than in mice. Stable anaesthesia is generally easier to achieve, and appropriate monitoring of physiological parameters less invasive in rats due to their larger body size (compared to mice). As transgenic rats are becoming more accessible (Pradhan and Majumdar, 2016), implications of species selection on the expected data quality may become increasingly important for assessing the construct validity of a given experiment.

Assuming that construct validity is given under a certain anaesthetic protocol in a certain rodent model, suboptimal monitoring and management introduce bias and thus reduce internal validity: When physiological parameter values are instable and/or deviate from normal ranges, BOLD fMRI readouts represent the combined effects of factors under investigation and physiological parameters. If potentially confounding changes or deviations in physiological parameters are not detected, conclusions about causal relationships are not feasible (Würbel et al., 2014), reproducibility of the results may be low, and comparison of results and synthesis in meta-analyses challenging, especially if results are conflicting.

But even when potentially confounding effects are detected with careful monitoring, interpretation of findings and comparison of results across studies may be challenging.

To minimise the risk that anaesthesia-related physiological parameter changes compromise internal validity of BOLD fMRI studies, standards of monitoring and ranges of acceptable fluctuations in physiological parameters should be established and broadly applied.

Some may argue that an over-standardisation of experiments, e.g., in terms of anaesthetic management, reduces external validity, so that findings cannot be generalised beyond the conditions under which they were acquired. Given the variable levels of monitoring used in the studies included in this review–which all explicitly investigated effects of physiological parameter changes on BOLD fMRI outcomes–implementing standards of monitoring and management would rather enhance overall validity than threaten external validity. To warrant external validity, heterogeneity should be included in studies in a controlled, systematic way rather than by failure to control for potential confounders.

## Conclusion

In this systematic review it was shown that p_a_O_2_, p_a_CO_2_ and arterial blood pressure affect BOLD signal across different types of BOLD fMRI, and BOLD fMRI studies risk to be confounded if physiological parameters are not monitored and accounted for. Establishing standards of monitoring–as well as evidence-based optimal dose ranges and imaging timepoints for a selection of anaesthetic protocols, see article b–is therefore a priority for improving the scientific validity of rodent fMRI studies.

## Data Availability Statement

The raw data supporting the conclusions of this article will be made available by the authors, without undue reservation.

## Author Contributions

RB-W, ARS, SH, and AS contributed to conception and design of the study. ARS and FR-B performed data extraction. ARS wrote the first draft of the manuscript. All authors contributed to manuscript revision, read, and approved the submitted version.

## Conflict of Interest

The authors declare that the research was conducted in the absence of any commercial or financial relationships that could be construed as a potential conflict of interest.

## Footnotes

^1^Evidence Partners. “DistillerSR”. Ottawa, Canada.

^2^https://www.zirp.uzh.ch/en/labanalysis/bloodchemistry-1.html

^3^https://www.nc3rs.org.uk/rat-decision-tree-blood-sampling

^4^https://www.nc3rs.org.uk/mouse-decision-tree-blood-sampling
