# Supplementary material for: Systematic Review: Anaesthetic Protocols and Management as Confounders in Rodent Blood Oxygen Level Dependent Functional Magnetic Resonance Imaging (BOLD fMRI)–Part A: Effects of Changes in Physiological Parameters
Source: Front Neurosci. 2020 Oct 23;14:577119. doi: 10.3389/fnins.2020.577119 (PMC7646331; doi:10.3389/fnins.2020.577119)
Supplement: Supplementary file 1 [file Data_Sheet_1.PDF]

Supplementary Material S1

Systematic review protocol as on the day the systematic search was conducted (16.08.2017): The impact of anaesthesia protocols on BOLD fMRI validity in laboratory rodents

1 Stage of the review at time of registration

An initial systematic search was performed, and records were selected and partially analyzed by a single author, when it was decided to update the search with refined methodology.

2 Objectives

Blood oxygen level dependent (BOLD) fMRI is the most commonly used modality for functional neuroimaging in humans (several thousand studies published per year) and widely used in preclinical and basic research rodent models (Martin, 2014; Jonckers et al., 2015; Pan et al., 2015). Changes in blood oxygenation levels are interpreted as a surrogate for neuronal activation, based on the mechanism of neurovascular coupling (i.e. activation of neurons increases local blood flow) (Logothetis and Wandell, 2004). As even small movements of the head during image acquisition distort the images, animals are typically anaesthetized for image acquisition. In terms of animal welfare, scanning under anaesthesia is preferable to awake scanning under rigorous fixation (Low et al., 2016). However, anaesthesia interferes with interpretation of the BOLD signal on several levels: first, alterations in physiological parameters (e.g. hypotension, hypercapnia) can alter local hemodynamics in absence of changes in neuronal activity. Second, anaesthetics per definition alter neuronal activation and information processing, so that measurements may reflect activation under a distinct anaesthetic rather than universal patterns. Third, anaesthetics may modulate signal cascades responsible for neurovascular coupling. In practice, it is difficult to clearly separate the relative contribution of each mechanism. Therefore, one part of the review will focus on studies which directly compare different anaesthetic protocols with each other or with awake scanning. We want to analyze how different states of anaesthesia affect the BOLD outcome measures specified in the respective studies in adult rats and mice. States of anaesthesia are defined as distinct if they differ by the drug or dose administered or by the time that has elapsed since induction. The other part of the review will focus on the specific effects of alterations in physiological parameters on different BOLD outcome measures in adult rats and mice. Physiological parameters that can typically be altered under anaesthesia are p_aCO_2, p_aO_2, SpO_2, respiratory rate, respiratory pattern, heart rate, arterial blood pressure and body temperature.

Due to the vast diversity of post-processing options in BOLD fMRI, any outcome measure specified by the individual studies is eligible for analysis, as long as the study uses BOLD contrast on the brain parenchyma (whole brain or specific regions of interest) in order to draw a conclusion about functional aspects of the brain.

Analysis will include studies conducted in healthy animals as well as studies in disease models, as emphasis is placed on providing researchers with relevant information for deliberately choosing an appropriate anaesthetic protocol for BOLD fMRI experiments in laboratory rodents.

In short, our research question reads as follows: What is the effect of a) different states of anaesthesia or b) changes in physiological parameters that can be observed under anaesthesia on the BOLD fMRI outcome measure defined by the individual study in adult rats and mice?

Our aim is to demonstrate the extent of anaesthetic protocol-related differences in fMRI outcomes, to formulate evidence-based minimal standards for monitoring during BOLD fMRI experiments, and ultimately to elaborate recommendations on how to choose an appropriate anaesthetic protocol. To our knowledge, this is the first systematic review about the impact of anaesthetic protocols on BOLD fMRI validity in laboratory rodents.

3 Methods

3.1 Search strategy and study identification

A systematic search will be conducted in EMBASE, MEDLINE, Scopus and Web of Science. Search terms are listed in the table below. Search terms within one block are linked with “OR”, while the blocks are linked with “AND”. Terms will be searched in titles and abstracts. Correspondent Emtree vocabulary and MeSH, if available, will additionally be used for the search in EMBASE and Medline, respectively. Language will be restricted to English, German and French. As the first publication describing BOLD contrast in MRI appeared in 1990, a filter for publication year 1990 or later will be used.

**Rodents**<br><br>rat OR rats OR mouse OR mice OR rodent OR rodents
**MRI**<br><br>((MRI OR MRT OR NMR OR “magnetic resonance imaging”) *proximity operator*5 functional) OR fMRI OR BOLD OR “Blood oxygen level dependent”
**Anaesthesia OR physiology**<br><br>anesthe* OR anaesthe* OR hypercapnia OR hyperoxia OR hypoxia OR apnea OR “blood pressure” OR hypotension OR hypertension OR autoregulation OR thermoregulation OR “physiological noise” OR “functional connectivity” OR somatosensory OR stimulation OR isoflurane OR sevoflurane OR halothane OR medetomidine OR dexmedetomidine OR alpha-chloralose OR chloralose OR a-chloralose OR urethane OR propofol OR ketamine OR xylazine

Additionally, any publication fulfilling inclusion criteria which is cited in reviews or original articles, but was not found by the systematic search, will be included.

If a selected study is recorded in the format of a proceeding/abstract/poster, google scholar will be used to search for a corresponding full article (for more details, see study selection).

3.2 Study selection

All records will be imported to endnote and duplicates removed with the “find duplicates” function. In a pre-screening phase, titles and abstracts will be screened. Records deemed eligible for analysis in the preliminary search automatically pass this stage. Records who have made it through pre-screening will undergo full text screening for eligibility. Study selection will be performed by a single reviewer. As there is not enough evidence to claim that study selection is performed by two reviewers (Shamseer et al., 2015), having a single reviewer performing this step can be justified against the common recommendations of having two reviewers independently screening the search results.

Inclusion and exclusion criteria are defined as follows:

	Inclusion criteria	Exclusion criteria
Type of study	Describing original research, reported in an article, abstract, conference proceeding or poster	Review; data originating from different experiment which is not described in material and methods.
Type of animals	Adult rats and mice of any strain.<br><br>Adult defined as sexually mature, i.e. >=12 weeks or 200 g for rats; >= 8 weeks or at least 18 g for mice. For both species the lower limit of reproductive activity of 12 months is used as the upper age limit (Wolfensohn and Lloyd, 2013). If articles report to use adult animals, but do not specifiy the age or weight, studies are included. If they report to used adult	Other species; neonate/juvenile/geriatric animals, if<br><br>a) defined as such by the study or<br><br>b) not explicitely described as adults and not fulfilling the age and weight limits defined under inclusion criteria

	animals but age and/or weight of some animals (e.g. if a range is given) are within 10% of deviation from the limits defined here, the study is still eligible.<br><br>Disease models are eligible.	
Type of intervention	BOLD fMRI<br><br>a) Comparison of anaesthetic protocols or anaesthetized vs. awake for same imaging protocol<br>b) alteration of physiological parameters: either deliberately caused by an intervention or closely monitored over time with the explicit intention (mention in abstract) of analyzing the correlation with fMRI signals.<br><br>Acceptable paradigms: resting state; peripheral sensory stimulation (e.g. electrical, mechanical, chemical, visual, auditory or olfactory stimulation) including noxious stimuli; direct brain stimulation (e.g. electrical, optogenetic; chemical if specifc neurotransmitter systems are blocked/enhanced in order to elucidate the effects and mechanisms of action of anaesthetics)	fMRI applied to other regions of the body than brain (e.g. spine, heart, joints);<br><br>modalities of fMRI other than BOLD (e.g. arterial spin labeling, CBV measurements);<br><br>BOLD fMRI studies neither comparing states of anaesthesia nor investigating alterations of physiological parameters;<br><br>pharmacologic stimulation for other purposes than elucidating the effects of mechanisms of action of anaesthetics by blocking/enhancing specific neurotransmitter systems.
Outcome measures	As defined by the respective study as long as BOLD	Correlations of BOLD signal with other functional

	contrast is used on the brain parenchyma to make a statement about functional aspects of the brain.	neuroimaging methods, measurements of neural activity or cerebral hemodynamics
Language restrictions	English, German, French	All other languages
Publication date restrictions	1990 and later	Earlier than 1990
Other	-	Duplicate not recognized by endnote; strong suspicion of multiple reporting

In the title and abstract screening phase, priority exclusion criteria are non-rodent species, applications of fMRI to other regions of the body, non-BOLD fMRI modalities and reviews. In the full text screening phase, the focus is on excluding records which neither compare anaesthetic protocols nor investigate the effect of alterations in physiological parameters or which use pharmacologic stimulation for other purposes than elucidating the effects of mechanisms of action of anaesthetics by blocking/enhancing specific neurotransmitter systems, and data originating from a different experiment which is not described in the material and methods section of the present publication. If a study describes multiple interventions and outcome measures, only data from those experiments meeting the inclusion criteria will be analyzed.

Duplicates not recognized by endnote (e.g. due to different recording of author names in different databases) will be manually resolved. If a proceeding/abstract/poster and a full article by the same author reporting the same experimental protocol with the same primary outcome are found, so that the full article most probably describes the same study as the shorter form of publication, only the full article is kept. In order to get the best information available about individual studies, we will try to find a corresponding full article on google scholar for each short form record, using a combination of author names, keywords and restriction of the publication date to the first 3 years following the shorter publication. If a full article is found, the shorter publication is replaced, otherwise it is kept.

DistillerSR will be used to assist the screening and data extraction process.

3.3 Study characteristics to be extracted (for assessment of external validity, reporting quality)

For each study, animal characteristics (species, sex, strain, age, weight, number per experimental group), the exact anaesthetic protocol (drug, dose, route of administration, time point of administration, gas mixture, flow, concentration of inhalant anaesthetic), surgical preparations for the experiment, presence or absence of monitoring of specific parameters and details on the methods and frequency of monitoring (temperature, heart rate, oxygen saturation, respiratory rate/ventilator setting, end-tidal gas concentrations, blood gas parameters and timing, arterial blood pressure, reflexes) are extracted. For records investigating the effect of physiological parameters on BOLD

fMRI outcome, details of the interventions to alter the physiological parameter(s) are additionally extracted. For all studies, magnetic field strength, duration of image acquisition and total experiment, type and timing of stimulations during BOLD fMRI, primary and secondary fMRI outcome as well as a brief description of additional methods used in that study are recorded. A separate column allows to comment on specific issues of the study that are not covered by the risk of bias tool (e.g. inadequate methods, failure to discuss potential confounders).

3.4 Assessment of risk of bias (internal validity)

One reviewer will assess the risk of bias in each study.

An adapted version of SYRCLE's risk of bias tool (ROB tool) will be used. As only one reviewer assesses the risk of bias, a first version of the ROB tool will be sent to experts from several fields (welfare of laboratory animals, anaesthesia, fMRI), together with the original ROB tool and 2-3 representative publications. Their feedback will be integrated and, if major revisions are made, a revised version sent out for a second round of feedback, the primary question being "Do you think that this version of the adapted risk of bias tool is more adequate than the previous?".

3.5 Collection of outcome data

A peculiarity of our study is that the outcomes are heterogeneous; any outcome measure defined by an individual study is eligible as long as it results from analysis of BOLD fMRI and its purpose is to measure a functional characteristic of the brain.

For each study it will be extracted whether a) a qualitative and b) a quantitative difference was observed between different states of anaesthesia or different values of physiological parameters (values of physiological parameters are considered different if they are defined as different by the respective study). If a difference was observed, it will be specified. For physiological parameters, the absolute values of baseline, change and altered state will be extracted.

A second reviewer will control whether data was extracted correctly because mistakes are common at this step of the workflow (Shamseer et al., 2015).

3.6 Data analysis/synthesis

Data will be analyzed strictly separated for each species, but following the same structure of analysis.

3.6.1 State of anaesthesia

Per imaging paradigm, it will be analyzed whether studies comparing the same anaesthetics or dosages of the same anaesthetic first consistently report the presence or absence of qualitative differences between different states of anaesthesia, and second, if qualitative differences are

observed, whether they are consistent, complementary or conflicting. An analog analysis will be performed for quantitative differences. For each comparison, a synthesis will be formulated, and the quality of evidence indicated.

Comparisons for which only single reports exist will be described as such.

Depending on the number of records about comparisons of states of anaesthesia in disease models, those records will be analyzed in a separate subchapter or along with studies performed in healthy animals, but it will be analyzed whether the presence and/or nature of observed differences are correlated with the health status of animals under investigation.

3.6.2 Physiological parameters

Per physiological parameter, it will be analyzed whether in- and decreases, respectively, are consistently reported to affect BOLD outcome measures, which will be grouped in the same imaging paradigm categories as used in the state of anaesthesia section. For changes in $PaCO_2$ and PaO_2, the constitution of inspired gases will also be considered. If qualitative or quantitative differences are reported, it will be analyzed whether they are consistent, complementary or conflicting. It will be further analyzed whether the anaesthetic used or the source of change of the physiological parameter correlates with the presence and/or nature of observed effects. If enough data is available, it will additionally be analyzed whether the changes in BOLD signal are correlated with the magnitude of change of the physiological parameter's value.

For each parameter, it will be concluded whether it potentially affects BOLD measurements if unstable. The strength of evidence will be indicated.

Meta-analysis will be considered, but a definitive decision whether it will be suitable cannot be made yet.

3.6.3 Grading of evidence

For each subcategory, the strength of evidence will be graded according to the evidence-based practice center approach (EPC) (Owens et al., 2009). The following categories will be assessed across records: risk of bias (low/moderate/high), consistency (yes/no for direction of effect and effect size), directness (direct/indirect) and precision (precise/imprecise). Together, those assessments result in the classification of the body of evidence for that specific subcategory as high, moderate, low or insufficient.
