# Supplementary material for: Systematic Review: Anaesthetic Protocols and Management as Confounders in Rodent Blood Oxygen Level Dependent Functional Magnetic Resonance Imaging (BOLD fMRI)–Part A: Effects of Changes in Physiological Parameters
Source: Front Neurosci. 2020 Oct 23;14:577119. doi: 10.3389/fnins.2020.577119 (PMC7646331; doi:10.3389/fnins.2020.577119)
Supplement: Supplementary file 2 [file Data_Sheet_2.pdf]

Supplementary Material S2

The adapted SYRCLE risk of bias tool

Questions can be answered with “yes”, indicating a low risk of bias, “no”, indicating a high risk of bias, and “unclear”, meaning that the risk of bias is unclear based on the information provided in the respective publication. Guidelines of all journals in which the included references are published were checked for specific requirements regarding blinding and randomization (list see below). None of those journals claimed to publish only randomized or blinded studies.

To summarize the risk of bias per study as well as across studies, the approach proposed in the Cochrane risk of bias tool will be used (Higgins et al., 2011).

1 Items

1. Selection bias – sequence generation

Describe the methods used, if any, to generate the allocation sequence in sufficient detail to allow an assessment whether it should produce comparable groups.

Was the allocation sequence adequately generated and applied?

Did the investigators describe a random component in the sequence generation process such as:
Referring to a random number table;
Using a computer random number generator.

Additional information

Examples of a non-random approach:

- Allocation by judgment or by investigator’s preference;
- Allocation based on the results of a laboratory test or a series of tests;
- Allocation by availability of the intervention;
- Sequence generated by odd or even date of birth;
- Sequence generated by some rule based on animal number or cage number.

Practical implementation

- If method described → yes or no depending on method
- If method not described → unclear

2. Selection bias – baseline characteristics

Describe animal characteristics, if any, that are compared in order to judge whether or not intervention and control groups were similar at the start of the experiment.

Were the groups similar at baseline or were they adjusted for confounders in the analysis?

Was the distribution of relevant baseline characteristics balanced for the intervention and control groups?

If relevant, did the investigators adequately adjust for unequal distribution of some relevant baseline characteristics in the analysis?

Additional information

Relevant baseline characteristics are age, sex and weigth; timepoint of baseline image acquisition relative to induction and eventual change of anaesthetic; response to stimulation at baseline.

Practical implementation

- If clearly similar → yes
- If partially similar, but not all relevant information reported → unclear
- If clearly different or no information on any of the relevant characteristics reported → no

3. Selection bias – allocation concealment

Describe the method used to conceal the allocation sequence in sufficient detail to determine whether intervention allocations could have been foreseen before or during enrolment.

Was the allocation adequately concealed?

Could the investigator allocating the animals to intervention or control group not foresee assignment due to one of the following or equivalent methods?

- Third-party coding of experimental and control group allocation
- Central randomization by a third party
- Sequentially numbered opaque, sealed envelopes

Additional information

Examples of investigators allocating the animals being possibly able to foresee assignments:

- Open randomization schedule
- Envelopes without appropriate safeguard
- Alternation or rotation

- Allocation based on date of birth
- Allocation based on animal number
- Any other explicitly unconcealed procedure of a non-random approach

Practical implementation

- If method described → yes or no according to list of positive and negative examples
- If method not described → unclear

4. Performance bias – blinding

Describe all measures used, if any, to blind trial caregivers and researchers from knowing which intervention each animal received. Provide any information relating to whether the intended blinding was effective.

Were the caregivers and /or investigators blinded from knowledge which intervention each animal received during the experiment? Were animals selected at random for outcome assessment?

Was blinding of caregivers and investigators ensured, and was it unlikely that their blinding could have been broken?

- ID cards of individual animals, or cage/animal labels are coded and identical in appearance.
- Administration of anaesthetics is performed by a person not involved in image processing and outcome assessment; the investigators cannot see the procedure and the vaporizer setting.
- The circumstances during the intervention are specified and similar in both groups.

Additional information

Examples of inappropriate blinding:

- Colored cage labels (red for group A, yellow group B)
- Expected differences in visible effects between control and experimental groups
- The individual who prepares the experiment is the same as the one who conducts and analyses the experiment
- Circumstances during the intervention are not similar in both groups
- Examples where circumstances during the intervention were not similar:
- Timing of administration of the placebo and exp drug was different; timing of imaging different between different groups
- Instruments used to conduct experiment differ between experimental and control group (e.g. skinning of skull for optical imaging or electrode insertion into the brain in one group, but not the other)

Practical implementation
As blinding is associated with additional work for the investigators and increases the level of evidence ascribed to a study, we expected that any effort to blind a study is at least briefly mentioned (e.g. “a blinded investigator”).

- If blinding described and expected to have been effective → yes
- If blinding mentioned, but not described how/doubts whether effective → unclear
- If no mention of blinding at all → no
- If for one aspect of the experiment clear that not blinded → no, because very unlikely that rest blinded

5. Detection bias – random outcome assessment

Describe whether or not animals were selected at random for outcome assessment, and which methods to select the animals, if any, were used.

Were animals selected at random for outcome assessment?

Did the investigators randomly pick an animal during outcome assessment, or did they use a random component in the sequence generation for outcome assessment?

- Referring to a random number table;
- Using a computer random number generator;
- Etc

Practical implementation

- If method described → yes or no depending on method
- If method not described → unclear

6. Detection bias – blinding

Describe all measures used, if any, to blind outcome assessors from knowing which intervention each animal received. Provide any information relating to whether the intended blinding was effective.

Was the outcome assessor blinded?

Was blinding of the outcome assessor ensured, and was it unlikely that blinding could have been broken?

- Outcome assessment methods were the same in
- both groups.
- Animals were selected at random during outcome
- assessment (use signaling questions of entry 6).

Was the outcome assessor not blinded, but do review authors judge that the outcome is not likely to be influenced by lack of blinding? (e.g., mortality)

Additional information

This item needs to be assessed for each main outcome.

Practical implementation

As blinding is associated with additional work for the investigators and increases the level of evidence ascribed to a study, we expected that any effort to blind a study is at least briefly mentioned (e.g. “a blinded investigator”).

- If blinding described and expected to have been effective → yes
- If blinding mentioned, but not described how/doubts whether effective → unclear
- If no mention of blinding at all → no
- If for one aspect of the experiment clear that not blinded → no, because very unlikely that rest blinded

7. Attrition bias – incomplete outcome data

Describe the completeness of outcome data for each main outcome, including attrition and exclusions from the analysis. State whether attritions and exclusions were reported, the numbers in each intervention group (compared with total randomized animals), reasons for attrition or exclusions, and any re-inclusions in analyses for the review.

Were incomplete data adequately addressed?

Were all animals included in the analysis?

Were the reasons for missing outcome data unlikely to be related to true outcome (e.g., technical failure)?
Are missing outcome data balanced in numbers across intervention groups, with similar reasons for missing data across groups?

Practical implementation

- If clearly stated that all animals were included → yes
- If neither stated that all animals included nor exclusions reported or if missing outcome data not balanced across groups → unclear
- If missing suspicion that exclusions related to true outcome or results reported for fewer animals than originally included without justification → no

8. **Reporting bias – selective outcome reporting**

State how selective outcome reporting was examined and what was found.

Are reports of the study free of selective outcome reporting?

Was the study protocol available and were all of the study's pre-specified primary and secondary outcomes reported in the current manuscript?

Was the study protocol not available, but was it clear that the published report included all expected outcomes (i.e. comparing methods and results section)?

Additional information

Selective outcome reporting:
- Not all of the study' s pre-specified primary outcomes have been reported;
- One or more primary outcomes have been reported using measurements, analysis methods or data subsets (e.g., subscales) that were not pre-specified in the protocol;
- One or more reported primary outcomes were not pre-specified (unless clear justification for their reporting has been provided, such as an unexpected
- adverse effect);
- The study report fails to include results for a key outcome that would be expected to have been reported for such a study.

Practical implementation
If no study protocol was available, the second question was decisive for the overall assessment of item 8.
- If no suspicion → yes
- If not sure → unclear
- If expected results were not reported → no

9. **Other – other source of bias**

State any important concerns about bias not covered by other domains in the tool.

Was the study apparently free of other problems that could result in a high risk of bias?

Was the study free of pooling drugs?

Additional information

Experiments in which animals receive – besides the intervention drug – additional treatment or drugs which might influence or bias the result. Eg. administration of inhalant anaesthetic together with N2O, but injectable anaesthetic administered without adding N2O or

administration of injectable anaesthetic associated with fluid administration, but no fluids administered in inhalant anaesthetic group.

Were design-specific risks of bias absent?

Additional information

Design-specific risks of bias:

- Crossover design that was not suitable (intervention with no temporary effect, or the disease is not stable over time)
- Crossover design with risk of carry-over effect
- Crossover design with only first period data being available
- Crossover design with many animals not receiving 2nd or following treatment due to large number of drop-outs probably due to longer duration of study
- Crossover design in which all animals received same order of interventions
- Multi-arm study in which the same comparisons of groups are not reported for all outcomes (selective outcome reporting)
- Multi-arm study in which results of different arms are combined (all data should be presented per group)
- Cluster randomized trial not taking clustering into account during statistical analysis (unit of analysis error)
- Crossover design in which paired analysis of the results is not taken into account

Practical implementation

- If pooling of drugs explicitly avoided and no concern about design-specific risk of bias → yes
- If pooling of drugs or design-specific risk of bias possible → unclear
- If pooling of drugs (e.g. if one group inhalants and other group injectables and fluid management not described) or clear design-specific risk of bias → no

2 Check of author guidelines

Journals for which author guidelines were checked and no specific requirements regarding blinding/randomization standards were found:

- Australasian Physical and Engineering Sciences in Medicine
- Biomedical Engineering - Applications, Basis and Communications
- Brain Connectivity
- Brain Research
- Brain Stimulation
- Brain Structure and Function
- Brain Topography
- Cerebral Cortex

- Chinese Journal of Physiology
- Epilepsia
- European Neuropsychopharmacology
- Frontiers in Neural Circuits
- Japanese Journal of Veterinary Research
- Journal of Cerebral Blood Flow & Metabolism
- Journal of Magnetic Resonance Imaging
- Journal of Neurophysiology
- Journal of Neuroscience
- Journal of Neuroscience Methods
- Magnetic Resonance Imaging
- Magnetic Resonance in Medicine
- NeuroImage.
- NeuroImage: Clinical
- Neuropsychopharmacology
- NeuroReport
- Neuroscience
- Neuroscience Letters
- NMR in Biomedicine
- Pharmacology
- PLoS Biology
- PLOSone
- Proceedings of the National Academy of Sciences of the United States of America
- Psychopharmacology
- RoFo Fortschritte auf dem Gebiete der Rontgenstrahlen und der Neuen Bildgebenden Verfahren
- Scientific Reports
- Stroke
- Zeitschrift Fur Medizinische Physik

Journals for which author guidelines were not found or not in English

- Advances in Experimental Medicine and Biology
- Chirurgia Italiana
