# Supplementary material for: Systematic Review: Anaesthetic Protocols and Management as Confounders in Rodent Blood Oxygen Level Dependent Functional Magnetic Resonance Imaging (BOLD fMRI)–Part A: Effects of Changes in Physiological Parameters
Source: Front Neurosci. 2020 Oct 23;14:577119. doi: 10.3389/fnins.2020.577119 (PMC7646331; doi:10.3389/fnins.2020.577119)
Supplement: Supplementary file 3 [file Data_Sheet_3.pdf]

Supplementary Material S3

Additional Results

1 Additional exclusion criteria

During study selection, additional exclusion criteria were defined. In addition to those mentioned in the main text, the following criteria were added during screening and study selection:

While descriptive reporting of qualitative aspects was accepted unless generalised to the point that almost no information could be extracted, studies presenting solely quantitative findings, such as percent signal changes, without any statement on statistical significance of the results were excluded.

Some studies used known effects of hypercapnia or hyperoxia to study differences in response to hyperoxic or hypercapnic challenges between experimental groups, without further characterising those responses, and were excluded.

Two studies were excluded because animals were imaged in a vertical position. As orthostatic arterial blood pressure regulation is blunted during anaesthesia (Duke-Novakovski et al., 2016), the haemodynamic status of vertically positioned animals was not considered comparable to the haemodynamic status of animals in horizontal position.

Additional specific cases are described in supplementary material S4.

The search also retrieved a few abstract collections or conference proceedings, typically containing several hundred abstracts. Those were excluded with the reasoning that individual potentially relevant abstracts could have been identified by the systematic search and are also listed in supplementary material S4.

2 Strain and sex distribution of animals

Sprague-Dawley rats were the most commonly used rat strain (57 references/55 datasets, of which 1 also used Wistar rats), followed by Wistar (31 references/30 datasets, of which 1 also used Sprague-Dawley rats) and Long-Evans rats (17 references/12 datasets). In three studies the rat strain was not specified. All mouse studies used C57BL/6 mice, and two studies additionally investigated other strains (BALB/c and I/LnJ in one study, transgenic animals expressing channel rhodopsin 2 in the other study). While primarily male rats were studied, both sexes were represented in mouse studies (figure S1).

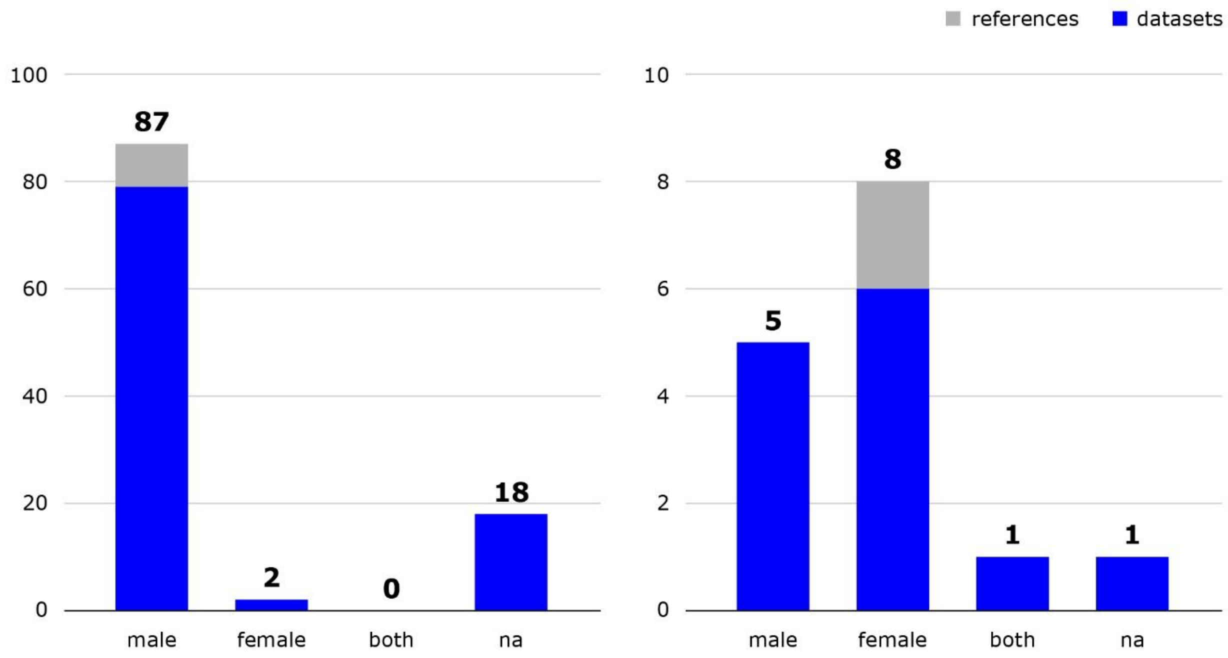

Figure S1. Sex distribution in studies investigating rats (left diagram) and mice (right diagram). Numbers of references and underlying datasets are shown. Male = study used only male animals, female = study used only female animals, both = study used animals of both sexes, na = sex of animals not reported
