# Supplementary material for: Systematic Review: Anaesthetic Protocols and Management as Confounders in Rodent Blood Oxygen Level Dependent Functional Magnetic Resonance Imaging (BOLD fMRI)–Part A: Effects of Changes in Physiological Parameters
Source: Front Neurosci. 2020 Oct 23;14:577119. doi: 10.3389/fnins.2020.577119 (PMC7646331; doi:10.3389/fnins.2020.577119)
Supplement: Supplementary file 4 [file Data_Sheet_4.pdf]

Supplementary Material S4

Inclusion and exclusion decisions

1 Inclusions

- Boonzaier et al. (2017): fMRI, repeated transcranial magnetic stimulation under different anaesthetic protocols, then check with fMRI for changes in fc – and there were differences. Decision that only baseline data is included.
- Schmidt et al. (2006): clearly declare in the abstract that they want to analyse the contribution of the change of physiological parameters on fMRI activation and they do so by investigating the time course. As the fMRI signal changes persisted after HR, BP and RR returned to baseline, the authors conclude that the influence of PP changes is negligible in this case. This conclusion is clearly written in the abstract (results and discussion don't really comment on it that explicitly). The study separately reports BOLD, CBF and $CMRO_2$. Therefore, there is no reason to exclude it. The study was not classified as interventional study because transient hypercapnia served just to derive maps of the calibration parameter M.
- Huang et al. (2013): Hypercapnia used as an application, therefore this part of the study excluded (and also because no comment on whether the observed change was significant, only the difference in response pre/post methylene blue investigated for significance (no significant difference found)). However, the comparison of responses to paw stimulation under hypoxia versus normoxia is included.
- Vanhoutte et al. (2006): included because explicitly described what happens with whole brain BOLD signal intensity when body core temperature in-/decreased.
- Baskerville et al. (2011): characterizes T2* response to 40 vs 100 % oxygen in penumbra of ischemic lesion. Not considered an application of hyperoxia because they explicitly test if 40% is an alternative to 100% which causes oxygen artefacts from oxygen in the sinuses.
- Bock et al. (1998): paw stimulation at baseline, then a 3 min hypercapnia scan without paw stimulation, 25 min later another paw stimulation scan; no lasting effect of hypercapnia on BOLD response detected. Included because it shows that effects of transient hypercapnia are limited to the duration of actual hypercapnia, which is relevant for monitoring guidelines.
- Sedlacik et al. (2015): "Correlation of oxygenation and perfusion sensitive MRI with invasive micro probe measurements in healthy mice brain" uses different inspiratory gas concentrations and reports among many other outcomes R2*.
- Pan et al. (2011): "Interestingly, while increasing the level of isoflurane from 1% to 1.8%, correlated BOLD fluctuations between hemispheres as well as coherent theta/delta LFP power fluctuations increased significantly" was at first sight the only sentence about rsfMRI results and appears only in discussion part. However, the same finding was also reported in results: the authors defined a neural suppression index, based on amount of suppression in burst-suppression EEG, as a proxy for anaesthetic level and report correlation of BOLD fluctuations with that neural suppression index.

- Chen et al. (2008): included because results from figure in text described (summary from images in fig. 3), although the word "weaker" may be not optimal – the images suggest that both less extended and lower signal change, but no quantitative analysis of % signal change between groups was provided.

2 Exclusions

2.1 Calibrated / quantitative fMRI without separate reporting of BOLD signal

- Wu et al. (2002): "Transient relationships among BOLD, CBV, and CBF changes in rat brain as detected by functional MRI". Aims of the study in its own words: 1. to experimentally determine how regional relationships between CBV and CBF fit the power law, 2. determine the transient relationships between CBF and CBV. The BOLD signal is separately described, but the article reports changes in BOLD signal during 7.5% and 10 % hypercapnia only in a graph, not in the text. It states that the displayed time courses were averages from those voxels that showed significant signal change during hypercapnia but does not report how many voxels did show a significant signal increase and how they were spatially distributed. Focus of the paper is on $CMRO_2$, parameter M, power index α, etc. Excluded as "insufficient detail of results" with the reason given here.
- Shu et al. (2016b): "Brain region and activity-dependent properties of M for calibrated fMRI". Compares medetomidine and α-chloralose only regarding parameter M, which is calculated as TE times R2' and thus not *sensu strictu* a BOLD measure (i.e. directly derived from signal intensity, R2* or T2*).
- Shu et al. (2016a): "Quantitative β mapping for calibrated fMRI"**.** Excluded because comparison of α-chloralose and medetomidine only regarding parameter β, which is part of a formula including $CMRO_2$. Signal intensity, T2* or R2* are not reported for the comparison of interest.

2.2 Application of hyperoxia or hypercapnia

- Kennan et al. (2004): application of hyperoxia in sickle cell model
- Elbel et al. (2000): abstract, considered an application of hypercapnia. Only the following sentence about hypercapnia: "By adding stepwise up to 10 percent CO_2 to the inspiratory gas mixture a generalized BOLD response was induced".
- Mahmoud et al. (2016): "AMP-activated Protein Kinase Deficiency Blocks the Hypoxic Ventilatory Response and Thus Precipitates Hypoventilation and Apnea" : Experimental hypoxia regarded as application (i.e. excluded) because of the following sentence: "Brainstem activity during hypoxia was therefore assessed by fMRI in anaesthetized mice in light of the fact that 9% or less O_2 induces cerebral vasodilation sufficient to eliminate the blood oxygen – level dependent (BOLD) signal driven, under normoxia, by increases in cerebral blood flow in response to sensory stimulation (27)".
- Ciobanu et al. (2015): uses hyperoxia as a "well known paradigm that mimics the BOLD response in absence of neuronal activation, by virtue of the diamagnetic character gained by haemoglobin upon activation" to compare three different pulse sequences. Excluded as application of hyperoxia.

- Kim et al. (2014): hypercapnia as an application to test difference between 2 strains; focus on (correlation with) CBF and CBV; just one graph showing increase of BOLD signal during hypercapnia
- Shih et al. (2011): hypercapnic challenge mentioned in methods, but no results reported; probably as an application
- Mitschelen et al. (2009): hypercapnia "as a stimulus to probe the responsiveness of the vasculature" in adult, healthy aged and cognitively impaired aged rats
- Henninger et al. (2007): application of hypercapnia to compare groups after traumatic brain injury. Control animals „robust“ BOLD response to CO_2 (whole brain slice coloured in figure), after TBI "severely" reduced and different recovery rates between regions. Nevertheless, it's an application, analogous to hyperoxia in sickle cell models.
- Paley et al. (2001): primarily application of hypercapnia, hyperoxia and hypoxia to test coherence of fMRI and optical imaging, additionally insufficient detail of results (no comment on significance of signal change). Focus of study on describing an MR device.
- Zhou et al. (2005): studies the interaction of magnetization transfer and BOLD effect : "The purpose of this study was to quantify the MTR changes in the brain as a function of arterial PCO_2 level and to use this dependence to study the interaction between the BOLD and MT effects in the parenchyma". As declared in that sentence, variation of inspired CO_2 concentrations served primarily to characterize magnetization transfer. Accordingly, there is only one sentence in the results that the unsaturated BOLD signal increased by 7% during hypercapnia and that this increase was stronger in the saturated condition. Or in their words: "It can be seen that there is a change of about 7% in the unsaturated signal intensities (standard SE BOLD effect) but an increased magnitude up to approximately 15% for the signal intensities under off-resonance RF irradiation (combined MT BOLD and standard SE BOLD effect) corresponding to a 100 mm Hg change in PCO_2." Excluded as application of hypercapnia and insufficient detail of results (no statement on significance).
- Wang et al. (2012): my understanding is that the "total" fMRI signal in this article is what is usually called the BOLD signal. It is known that other mechanisms contribute to the BOLD signal and what this study does is to investigate the contribution of perfusion or large vessel inflow to the total signal under hypercapnia, using hypercapnia as a method to increase CBF, i.e. an application. The focus is not on characterizing the response of the total signal to hypercapnia, but to elucidate the contributing mechanisms.

2.3 Anaesthetics not reported

- Williams et al. (2013): "Minocycline interferes with glutamate neurotransmission in an animal model of psychosis and with neurovascular coupling". Administers ketamine on top, but anaesthetics used for imaging not reported. Additionally, characterising ketamine effects simply as causing "widespread activation" would fulfil the criteria for insufficient detail of results.
- Dunn et al. (1996): short form, clearly describes regional difference in response to hypoxia (cortex vs caudate putamen), but excluded because anaesthetics not reported.

2.4 No results for the comparison of interest

- He et al. (2008): investigates whether one can conclude from BOLD signal to oxygen saturation but doesn't describe how the BOLD signal differed between α-chloralose and isoflurane. Excluded as "no results reported for comparison of interest".
- Madularu et al. (2017)
- Pronger et al. (2014): "Mulitimodal neuroimaging with hypercapnia to monitor cerebrovascular function after traumatic brain injury and evaluate treatment". Only an abstract; compared isoflurane with medetomidine, but no results reported. All four databases and google scholar searched by name of first author for potential follow-up articles, but nothing found.
- Jones et al. (1996): values in a table plus a graph indicate that R2* increases during anoxia - I suppose that this must be a consistent and significant phenomenon, because the whole results and discussion section just compare the change of R2* during anoxia between ischemic and non-ischemic regions and between ischemia and reperfusion phases. However, as this study reports "numbers", but does not provide information about the statistical significance for the comparison of interest, and does not even descriptively summarize what happens to R2* in general during anoxia, it was excluded as "no results reported for the comparison of interest" and "insufficient detail of results".

2.5 Focus on vascular aspects

- Desjardins et al. (2014): focus on vascular aspects and correlation of BOLD with other modalities; only one graph depicting the time course of the BOLD signal after transient hypercapnia. Excluded as no results reported for the comparison of interest
- 1990 Ogawa et al. (1990): described changes apply to vessels, not parenchyma
- Ciobanu et al. (2012): vessels, not parenchyma imaged
- Uhrig et al. (2014): vessels, not parenchyma imaged

2.6 Insufficient detail of result only reason

- Wang et al. (2015): evaluates a method that measures BOLD and CBF concurrently. Exposed rats to hypercapnia and ischemia to test the method under disturbances. The BOLD signal is reported to increase under hypercapnia (4.5 %) and decrease under ischemia (23 %), however, whether these changes are significant is not reported. Further analysis of the BOLD signal only addresses correlation with other measures. Therefore, the study was excluded (reason: insufficient detail of results).
- Lahti et al. (1997): the subtraction is baseline image awake 1 minus baseline image awake 2 and NOT awake vs anaesthetized as assumed previously (see methods section). Regarding the awake vs anaesthetized comparison, there is only one sentence in the methods: "The relatively large BOLD signal intensity change observed in this study may be due to the increased neuronal activation status of conscious animal, compared to the anesthetized counterpart." This information comes without any further characterisation or assessment of significance and consequently the study was excluded as insufficient detail of results.
- Lin et al. (1999): "A marked decrease in R2* was observed initially (from roughly 150 to 500 seconds) in response to the change in the CO_2 content of the inspired gas followed by a relative plateau throughout the end of the experiment. (…) Similar temporal behaviour for

ΔR2* in response to hypercapnia was observed in all rats." No comment on significance, therefore excluded as "insufficient detail". The focus of this study is that you cannot derive cerebral blood oxygen saturation from ΔR2* because CBV has a strong effect. Authors describe method ad quantitative fMRI, but to me more similar to TRUST described in the textbook by Uludağ and Uğurbil (2015).

2.7 Singular reasons

- Nair and Duong (2004) and Ahrens and Dubowitz (2001) **placed the mice vertically in the scanner (vertical bore)**. Both studies were excluded because we considered haemodynamics in this position not comparable with haemodynamics in horizontal positioning.
- Morton et al. (2002): "Systemic theophylline augments the blood oxygen level-dependent response to forepaw stimulation in rats". Used theophylline to inhibit cerebral vasodilation in response to neuronal activation and observed that the BOLD signal change during paw stimulation increased under theophylline while blood pressure and other systemic physiological parameters remained stable. As the aim of giving theophylline was explicitly not to modulate systemic physiological parameters, this intervention does not qualify as investigation of physiological parameter effects and the article was excluded.
- Two related articles:
  - Kettunen et al. (2001): "Cerebral T1ρ relaxation time increases immediately upon global ischemia in the rat independently of blood glucose and anoxic depolarization" was considered an "other MRI" earlier in screening stage 2 but would have been excluded anyway because of the lack of AP/PP.
  - Kettunen et al. (2002): "Effects of intracellular pH, blood, and tissue oxygen tension on T1ρ relaxation in rat brain" focuses as well on T1ρ as main MRI outcome, but uses different FiO_2 and $FiCO_2$. "In the in vivo experiments blood relaxation and cerebral blood volume (CBV) results were used for a two-compartment model to compute tissue T1 relaxation separately from the parenchymal MRI data." In the results, there are two sentences about BOLD: "During hypoxia, a negative BOLD was evident as T2 was lowered by 3.3 +/- 0.6 ms." And "the increase in T2 due to a BOLD effect was fully expressed at P_aCO_2 of around 80 mmHg, consistent with a previous cat study showing that OER decrease stabilized at a hypercapnia level of around 80 mmHg". The rest of the text deals with T1rho. The study is therefore excluded as well and classified as "other MRI".
- Kipervaser et al. (2007): "Statistical framework and noise sensitivity of the amplitude radial correlation contrast method". Different anaesthetics (and concentrations) are used as a mean to test a statistical model. Excluded as other, reason: different states of anaesthesia just to test statistical model
- Plaschke et al. (2006): chronic effects of hypotension (as a method to induce transient cerebral oligemia) are investigated; however for imaging studies effects of acute hypotension are relevant
- Martin et al. (2009); Martin et al. (2013) (abstract and full article with same title): applied hypercapnia in awake animals → excluded because not a naturally occurring problem in awake animals

- Ogawa et al. (1993): "The venous blood oxygenation, monitored at the sagittal sinus with the blood water T, measurement, were varied by changing the depth of anaesthesia with halothane." → excluded as other. Changing the depth of anaesthesia is not an accepted method to modulate physiological parameters, because the effects of anaesthetic depth and change in oxygenation cannot be separated.
- Thomas et al. (2002): focus on describing a (back then new) imaging sequence, hypoxia only in 2 rats, some changes in T2(*) reported at the end of results section. Decrease of T2* during hypoxia; "reproducible" time course "changes are in the range 10 – 15 ms for T*2 and 5 – 10 ms for T2. This corresponds to a reduction in SpO_2 from 95% to a minimum value of approximately 60%". They "validate" their observations of T2* under hypoxia (5 episodes per animal, 2 animals) against one observation of T2* time course under normoxia. Excluded first because only one animal for control and second because no comment on significance, also not in methods or discussion (although I admit that it is probably reasonable NOT to use statistics here, but at some point I have to stick with defined criteria and this is clearly a quantitative outcome).
- Cash et al. (2003): 6 animals undergoing MRI, four "groups", but not clear how many animals per group or if maybe even crossover. Excluded because realistic chance that n=1 per condition. "one animal per condition" selected as reason of exclusion.
- Easton et al. (2009): does not fulfil definition of an observational physiological parameter study, because intention to investigate effects of systemic physiological parameters on BOLD signal not expressed in abstract. But even if it would: title and axis are not matching in graphs so that it is not clear which graph shows what and results are not interpretable!

2.8 Abstract collections

Abstract collections are explicitly labelled as such in the title and, if an "abstract" is displayed in DistillerSR, it typically states something like "This proceeding contains 476 papers". Abstract collections were not manually screened but excluded *in toto*.

- Abstracts from the 11th Congress of the European Association of Neuro-Oncology (2014)
- 11th Turkish Neuroscience Congress (2013)
- 3rd Biennial Conference on Resting State Brain Connectivity(2012)
- Abstracts of the 20th Congress of the European Sleep Research Society (2010)
- Abstracts and Programme - EUROANAESTHESIA 2009: The European Anaesthesiology Congress (2009c)
- 9th International Symposium on NeuroVirology (2009a)
- 2011 5th International IEEE/EMBS Conference on Neural Engineering, NER 2011 (2011)
- 13th International Conference on Biomedical Engineering, ICBME 2008
- (2009b)

References

(2009a). 9th International Symposium on NeuroVirology. Journal of NeuroVirology *15*.

(2009b). 13th International Conference on Biomedical Engineering, ICBME 2008. 13th International Conference on Biomedical Engineering, ICBME 2008 *23*.

(2009c). Abstracts and Programme - EUROANAESTHESIA 2009: The European Anaesthesiology Congress. European Journal of Anaesthesiology *26*.

(2010). Abstracts of the 20th Congress of the European Sleep Research Society. Journal of Sleep Research *19*.

(2011). 2011 5th International IEEE/EMBS Conference on Neural Engineering, NER 2011. 2011 5th International IEEE/EMBS Conference on Neural Engineering, NER 2011.

(2012). 3rd Biennial Conference on Resting State Brain Connectivity. Brain Connectivity *2*.

(2013). 11th Turkish Neuroscience Congress. Journal of Neurological Sciences *30*.

(2014). Abstracts from the 11th Congress of the European Association of Neuro-Oncology. Neuro-Oncology *16*.

Ahrens, E.T., and Dubowitz, D.J. (2001). Peripheral somatosensory fMRI in mouse at 11.7 T. NMR in Biomedicine *14*, 318-324.

Baskerville, T.A., Deuchar, G.A., McCabe, C., Robertson, C.A., Holmes, W.M., Santosh, C., and Macrae, I.M. (2011). Influence of 100% and 40% oxygen on penumbral blood flow, oxygen level, and T2 -weighted MRI in a rat stroke model. Journal of Cerebral Blood Flow & Metabolism *31*, 1799-1806.

Bock, C., Schmitz, B., Kerskens, C.M., Gyngell, M.L., Hossmann, K.A., and Hoehn-Berlage, M. (1998). Functional MRI of somatosensory activation in rat: Effect of hypercapnic up-regulation on perfusion- and BOLD-imaging. Magnet Reson Med *39*, 457-461.

Boonzaier, J., Van Tilborg, G.A.F., Straathof, M., Petrov, P.I., Van Heijningen, C.L., Van Vliet, G., Smirnov, N., Van Der Toorn, A., Neggers, S.F., and Dijkhuizen, R.M. (2017). Differential outcomes of rTMS and anesthesia effects on functional connectivity in the rat brain. Brain Stimulation *10*, 418.

Cash, D., Read, S., Lythgoe, D., Williams, S., Roberts, T., Ireland, M., Smart, S., and Hunter, A. (2003). Autoradiographic and functional MRI of rat brain response to amphetamine under halothane and alpha-chloralose anaesthesia. Paper presented at: J Psychopharmacol (SAGE PUBLICATIONS LTD 6 BONHILL STREET, LONDON EC2A 4PU, ENGLAND).

Chen, C.M., Shih, Y.Y.I., Siow, T.Y., Chiang, Y.C., Chang, C., and Jaw, F.S. (2008). Antinociceptive effect of morphine in α-chloralose and isoflurane anesthetized rats using bold fMRI. Biomedical Engineering - Applications, Basis and Communications *20*, 39-46.

Ciobanu, L., Reynaud, O., Uhrig, L., Jarraya, B., and Le Bihan, D. (2012). Effects of Anesthetic Agents on Brain Blood Oxygenation Level Revealed with Ultra-High Field MRI. Plos One *7*, 5.

Ciobanu, L., Solomon, E., Pyatigorskaya, N., Roussel, T., Le Bihan, D., and Frydman, L. (2015). FMRI contrast at high and ultrahigh magnetic fields: Insight from complementary methods. NeuroImage *113*, 37-43.

Desjardins, M., Berti, R., Pouliot, P., Dubeau, S., and Lesage, F. (2014). Multimodal study of the hemodynamic response to hypercapnia in anesthetized aged rats. Neurosci Lett *563*, 33-37.

Dunn, J.F., Ding, S., Azzawi, A., Rolett, E.L., and Weaver, J.B. (1996). Hypoxia induced heterogeneity in cerebral vascular response measured using functional MRI. FASEB Journal *10*.

Easton, N., Marshall, F.H., Marsden, C.A., and Fone, K.C.F. (2009). Mapping the central effects of methylphenidate in the rat using pharmacological MRI BOLD contrast. Neuropharmacology *57*, 653-664.

Elbel, G.K., Kalisch, R., Schadrack, J., Droese, D.G., Hipp, R.F., and Auer, D.P. (2000). Anesthesia and monitoring for FMRI experiments at 7T in rats. Anesth Analg *90*.

He, X., Zhu, M., and Yablonskiy, D.A. (2008). Validation of oxygen extraction fraction measurement by qBOLD technique. Magnet Reson Med *60*, 882-888.

Henninger, N.M.D., Sicard, K.M.P., Li, Z.P.M.D., Kulkarni, P.P., Dutzmann, S.M.D., Urbanek, C.M.D., Schwab, S.M.D., and Fisher, M.M.D. (2007). Differential recovery of behavioral status and brain function assessed with functional magnetic resonance imaging after mild traumatic brain injury in the rat *. Critical Care Medicine *35*, 2607-2614.

Huang, S., Du, F., Shih, Y.Y.I., Shen, Q., Gonzalez-Lima, F., and Duong, T.Q. (2013). Methylene blue potentiates stimulus-evoked fMRI responses and cerebral oxygen consumption during normoxia and hypoxia. NeuroImage *72*, 237-242.

Jones, R.A., Müller, T.B., Haraldseth, O., Baptista, A.M., and Øksendal, A.N. (1996). Cerebrovascular changes in rats during ischemia and reperfusion: A comparison of BOLD and first pass bolus tracking techniques. Magnet Reson Med *35*, 489-496.

Kennan, R.P., Suzuka, S.M., Nagel, R.L., and Fabry, M.E. (2004). Decreased Cerebral Perfusion Correlates with Increased BOLD Hyperoxia Response in Transgenic Mouse Models of Sickle Cell Disease. Magnet Reson Med *51*, 525-532.

Kettunen, M.I., Gröhn, O.H.J., Penttonen, M., and Kauppinen, R.A. (2001). Cerebral T1p relaxation time increases immediately upon global ischemia in the rat independently of blood glucose and anoxic depolarization. Magnet Reson Med *46*, 565-572.

Kettunen, M.I., Grohn, O.H.J., Silvennoinen, M.J., Penttonen, M., and Kauppinen, R.A. (2002). Effects of intracellular pH, blood, and tissue oxygen tension on T-1 rho relaxation in rat brain. Magnet Reson Med *48*, 470-477.

Kim, T., Jennings, J.R., and Kim, S.G. (2014). Regional cerebral blood flow and arterial blood volume and their reactivity to hypercapnia in hypertensive and normotensive rats. J Cerebr Blood F Met *34*, 408-414.

Kipervaser, Z.G., Pelled, G., and Goelman, G. (2007). Statistical framework and noise sensitivity of the amplitude radial correlation contrast method. Magnet Reson Med *58*, 554-561.

Lahti, K.M., Ferris, C.F., Sotak, C.H., and King, J.A. (1997). Functional MRI in conscious rats using electrical stimulation of a hind paw. Proceedings of the 1997 23rd Annual Northeast Bioengineering Conference, 61-62.

Lin, W., Celik, A., Paczynski, R.P., Hsu, C.Y., and Powers, W.J. (1999). Quantitative magnetic resonance imaging in experimental hypercapnia: Improvement in the relation between changes in brain R2* and the oxygen saturation of venous blood after correction for changes in cerebral blood volume. J Cerebr Blood F Met *19*, 853-862.

Madularu, D., Mathieu, A.P., Kumaragamage, C., Reynolds, L.M., Near, J., Flores, C., and Rajah, M.N. (2017). A non-invasive restraining system for awake mouse imaging. J Neurosci Meth *287*, 53-57.

Mahmoud, A.D., Lewis, S., Juricic, L., Udoh, U.-A., Hartmann, S., Jansen, M.A., Ogunbayo, O.A., Puggioni, P., Holmes, A.P., Kumar, P., *et al.* (2016). AMP-activated Protein Kinase Deficiency Blocks the Hypoxic Ventilatory Response and Thus Precipitates Hypoventilation and Apnea. American Journal of Respiratory & Critical Care Medicine *193*, 1032-1043.

Martin, C., Kennerley, A., Berwick, J., Sibson, N., and Mayhew, J. (2009). Functional magnetic resonance imaging in un-anaesthetized rats using a chronically implanted surface coil. J Cerebr Blood F Met *29*, S606-S607.

Martin, C.J., Kennerley, A.J., Berwick, J., Port, M., and Mayhew, J.E. (2013). Functional MRI in conscious rats using a chronically implanted surface coil. J Magn Reson Imaging *38*, 739-744.

Mitschelen, M., Garteiser, P., Carnes, B.A., Farley, J.A., Doblas, S., DeMoe, J.H., Warrington, J.P., Yan, H., Nicolle, M.M., Towner, R., *et al.* (2009). Basal and hypercapnia-altered cerebrovascular perfusion predict mild cognitive impairment in aging rodents. Neuroscience *164*, 918-928.

Morton, D.W., Maravilla, K.R., Meno, J.R., and Winn, H.R. (2002). Systemic theophylline augments the blood oxygen level-dependent response to forepaw stimulation in rats. Am J Neuroradiol *23,* 588-593.

Nair, G., and Duong, T.Q. (2004). Echo-planar BOLD-fMRI of mice on a narrow-bore 9.4 T magnet. Magnet Reson Med *52,* 430-434.

Ogawa, S., Lee, T.-M., Kay, A.R., and Tank, D.W. (1990). Brain magnetic resonance imaging with contrast dependent on blood oxygenation. Proceedings of the National Academy of Sciences *87,* 9868-9872.

Ogawa, S., Lee, T.M., and Barrere, B. (1993). The sensitivity of magnetic resonance image signals of a rat brain to changes in the cerebral venous blood oxygenation. Magnet Reson Med *29,* 205-210.

Paley, M., Mayhew, J.E., Martindale, A.J., McGinley, J., Berwick, J., Coffey, P., Redgrave, P., Furness, P., Port, M., Ham, A., *et al.* (2001). Design and initial evaluation of a low-cost 3-Tesla research system for combined optical and functional MR imaging with interventional capability. Journal of Magnetic Resonance Imaging *13,* 87-92.

Pan, W.J., Thompson, G., Magnuson, M., Majeed, W., Jaeger, D., and Keilholz, S. (2011). Broadband Local Field Potentials Correlate with Spontaneous Fluctuations in Functional Magnetic Resonance Imaging Signals in the Rat Somatosensory Cortex Under Isoflurane Anesthesia. Brain Connectivity *1,* 119-131.

Plaschke, K., Bardenheuer, H.J., Martin, E., Sartor, K., and Heiland, S. (2006). Evolution of apparent diffusion coefficient and transverse relaxation time (T2) in the subchronic stage of global cerebral oligemia in different rat models. Experimental Brain Research *169,* 361-368.

Pronger, A.M., Korotcov, A., Bosomtwi, A., Jones, S., Selwyn, R.G., and Diaz-Arrastia, R. (2014). Mulitimodal neuroimaging with hypercapnia to monitor cerebrovascular function after traumatic brain injury and evaluate treatment. Journal of Neurotrauma *31,* A26.

Schmidt, K.F., Febo, M., Shen, Q., Luo, F., Sicard, K.M., Ferris, C.F., Stein, E.A., and Duong, T.Q. (2006). Hemodynamic and metabolic changes induced by cocaine in anesthetized rat observed with multimodal functional MRI. Psychopharmacology *185,* 479-486.

Sedlacik, J., Reitz, M., Bolar, D.S., Adalsteinsson, E., Schmidt, N.O., and Fiehler, J. (2015). Correlation of oxygenation and perfusion sensitive MRI with invasive micro probe measurements in healthy mice brain. Z Med Phys *25,* 77-85.

Shih, Y.Y.I., Wey, H.Y., De La Garza, B.H., and Duong, T.Q. (2011). Striatal and cortical BOLD, blood flow, blood volume, oxygen consumption, and glucose consumption changes in noxious forepaw electrical stimulation. J Cerebr Blood F Met *31,* 832-841.

Shu, C.Y., Sanganahalli, B.G., Coman, D., Herman, P., Rothman, D.L., and Hyder, F. (2016a). Quantitative β mapping for calibrated fMRI. NeuroImage *126*, 219-228.

Shu, C.Y.a., Herman, P.b., Coman, D.b., Sanganahalli, B.G.b., Wang, H.b., Juchem, C.b.c., Rothman, D.L.a.b., de Graaf, R.A.a.b., and Hyder, F.a.b. (2016b). Brain region and activity-dependent properties of M for calibrated fMRI. Neuroimage *125 Supplement*, 848-856.

Thomas, D.L., Lythgoe, M.F., Gadian, D.G., and Ordidge, R.J. (2002). Rapid simultaneous mapping of T2 and T2* by Multiple acquisition of spin and gradient echoes using interleaved echo planar imaging (MASAGE-IEPI). NeuroImage *15*, 992-1002.

Uhrig, L., Ciobanu, L., Djemai, B., Le Bihan, D., and Jarraya, B. (2014). Sedation agents differentially modulate cortical and subcortical blood oxygenation: evidence from ultra-high field MRI at 17.2 T. PLoS ONE [Electronic Resource] *9*, e100323.

Uludağ, K., and Uğurbil, K. (2015). Physiology and Physics of the fMRI Signal. In fMRI: From Nuclear Spins to Brain Functions (Springer), pp. 163-213.

Vanhoutte, G., Verhoye, M., and Van Der Linden, A. (2006). Changing body temperature affects the T2* signal in the rat brain and reveals hypothalamic activity. Magnet Reson Med *55*, 1006-1012.

Wang, X., Zhu, X.H., Zhang, Y., and Chen, W. (2012). Large enhancement of perfusion contribution on fMRI signal. J Cerebr Blood F Met *32*, 907-918.

Wang, X., Zhu, X.H., Zhang, Y., and Chen, W. (2015). Simultaneous imaging of CBF change and BOLD with saturation-recovery-T<inf>1</inf> method. PLoS ONE *10*.

Williams, S.R., Hodkinson, D., Cash, D., Gigg, J., Williams, S.C., and Deakin, J.W. (2013). Minocycline interferes with glutamate neurotransmission in an animal model of psychosis and with neurovascular coupling. Journal of Neurochemistry *125*, 233-234.

Wu, G., Luo, F., Li, Z., Zhao, X., and Li, S.J. (2002). Transient relationships among BOLD, CBV, and CBF changes in rat brain as detected by functional MRI. Magnet Reson Med *48*, 987-993.

Zhou, J., Payen, J.-F., and van Zijl, P.C.M. (2005). The interaction between magnetization transfer and blood-oxygen-level-dependent effects. Magnet Reson Med *53*, 356-366.
