# Supplementary material for: Systematic Review: Anaesthetic Protocols and Management as Confounders in Rodent Blood Oxygen Level Dependent Functional Magnetic Resonance Imaging (BOLD fMRI)–Part A: Effects of Changes in Physiological Parameters
Source: Front Neurosci. 2020 Oct 23;14:577119. doi: 10.3389/fnins.2020.577119 (PMC7646331; doi:10.3389/fnins.2020.577119)
Supplement: Supplementary file 5 [file Data_Sheet_5.pdf]

Supplementary material S5

Included References

**Identification number in DistillerSR**	**Author**	**Title**	**Year**
13142	Abe, Y., Tsurugizawa, T.,Le Bihan, D.	Water diffusion closely reveals neural activity status in rat brain loci affected by anesthesia	2017
13849	Airaksinen, A. M.,Hekmatyar, S. K.,Jerome, N.,Niskanen, J. P.,Huttunen, J. K.,Pitkänen, A.,Kauppinen, R. A.,Gröhn, O. H.	Simultaneous BOLD fMRI and local field potential measurements during kainic acid-induced seizures	2012
14199	Asanuma, T., Yasui, H.,Sato, M.,Inanami, O.,Kuwabara, M.	A BOLD-fMRI study of cerebral activation induced by injection of algesic chemical substances into the anesthetized rat forepaw	2008
14459	Austin, V. C., Blamire, A. M.,Allers, K. A.,Sharp, T.,Styles, P.,Matthews, P. M.,Sibson, N. R.	Confounding effects of anesthesia on functional activation in rodent brain: A study of halothane and α-chloralose anesthesia	2005
20115	Baskerville, T. A., Deuchar, G. A.,McCabe, C.,Robertson, C. A.,Holmes, W. M.,Santosh, C.,Macrae, I. M.	Influence of 100% and 40% oxygen on penumbral blood flow, oxygen level, and T2 -weighted MRI in a rat stroke model	2011
13374	Bettinardi, R. G.,Tort-Colet, N.,Ruiz-Mejias, M.,Sanchez-Vives, M. V.,Deco, G.	Gradual emergence of spontaneous correlated brain activity during fading of general anesthesia in rats: Evidences from fMRI and local field potentials	2015
14715	Bock, C.,Schmitz, B.,Kerskens, C. M.,Gyngell, M. L.,Hossmann, K. A.,Hoehn-Berlage, M.	Functional MRI of somatosensory activation in rat: Effect of hypercapnic up-regulation on perfusion- and BOLD-imaging	1998
23151	Boonzaier, J.,Van Tilborg, G. A. F.,Straathof, M.,Petrov, P. I.,Van Heijningen, C. L.,Van Vliet, G.,Smirnov, N.,Van Der Toorn, A.,Neggers, S. F.,Dijkhuizen, R. M.	Differential outcomes of rTMS and anesthesia effects on functional connectivity in the rat brain	2017
14533	Brevard, M. E.,Duong, T. Q.,King, J. A.,Ferris, C. F.	Changes in MRI signal intensity during hypercapnic challenge under conscious and anesthetized conditions	2003

13184	Brynildsen, J. K.,Hsu, L. M.,Ross, T. J.,Stein, E. A.,Yang, Y.,Lu, H.	Physiological characterization of a robust survival rodent fMRI method	2017
13169	Bukhari, Q.,Schroeter, A.,Cole, D. M.,Rudin, M.	Resting state fMRI in mice reveals anesthesia specific signatures of brain functional networks and their interactions	2017
13242	Chang, P. C., Procissi, D.,Bao, Q.,Centeno, M. V.,Baria, A.,Apkarian, A. V.	Novel method for functional brain imaging in awake minimally restrained rats	2016
13500	Chao, T. H. H.,Chen, J. H.,Yen, C. T.	Repeated BOLD-fMRI imaging of deep brain stimulation responses in rats	2014
14234	Chen, C. M.,Shih, Y. Y. I.,Siow, T. Y.,Chiang, Y. C.,Chang, C.,Jaw, F. S.	Antinociceptive effect of morphine in α-chloralose and isoflurane anesthetized rats using bold fMRI	2008
14445	Dashti, M., Geso, M.,Williams, J.	The effects of anaesthesia on cortical stimulation in rats: A functional MRI study	2005
13995	de Celis Alonso, B., Makarova, T.,Hess, A.	On the use of α-chloralose for repeated BOLD fMRI measurements in rats	2011
13988	Desai, M.,Kahn, I.,Knoblich, U.,Bernstein, J.,Atallah, H.,Yang, A.,Kopell, N.,Buckner, R. L.,Graybiel, A. M.,Moore, C. I.,Boyden, E. S.	Mapping brain networks in awake mice using combined optical neural control and fMRI	2011
14721	Dunn, J. F., Swartz, H. M.	Blood oxygenation: Heterogeneity of hypoxic tissues monitored using bold MR imaging	1997
14678	Dunn, J. F.,Zaim Wadghiri, Y.,Meyerand, M. E.	Regional heterogeneity in the brain's response to hypoxia measured using BOLD MR imaging	1999
14321	Duong, T. Q.	Cerebral blood flow and BOLD fMRI responses to hypoxia in awake and anesthetized rats	2007
14584	Dutka, M. V., Scanley, B. E., Does, M. D.,Gore, J. C.	Changes in CBF-BOLD coupling detected by MRI during and after repeated transient hypercapnia in rat	2002
13525	Gass, N.,Schwarz, A. J.,Sartorius, A.,Schenker, E.,Risterucci, C.,Spedding, M.,Zheng, L.,Meyer-Lindenberg, A.,Weber-Fahr, W.	Sub-anesthetic ketamine modulates intrinsic BOLD connectivity within the hippocampal-prefrontal circuit in the rat	2014

13186	Gill, R. S., Mirsattari, S. M.,Leung, L. S.	Resting state functional network disruptions in a kainic acid model of temporal lobe epilepsy	2017
13482	Gong, L.,Li, B.,Wu, R.,Li, A.,Xu, F.	Brain-state dependent uncoupling of BOLD and local field potentials in laminar olfactory bulb	2014
14746	Graham, G. D.,Zhong, J.,Petroff, O. A. C.,Constable, R. T.,Prichard, J. W.,Gore, J. C.	BOLD MRI monitoring of changes in cerebral perfusion induced by acetazolamide and hypercarbia in the rat	1994
13136	Grandjean, J., Preti, M. G.,Bolton, T. A. W.,Buerge, M.,Seifritz, E.,Pryce, C. R.,Van De Ville, D.,Rudin, M.	Dynamic reorganization of intrinsic functional networks in the mouse brain	2017
13471	Grandjean, J.,Schroeter, A.,Batata, I.,Rudin, M.	Optimization of anesthesia protocol for resting-state fMRI in mice based on differential effects of anesthetics on functional connectivity patterns	2014
13345	Grimm, O., Gass, N.,Weber-Fahr, W.,Sartorius, A.,Schenker, E.,Spedding, M.,Risterucci, C.,Schweiger, J. I.,Böhringer, A.,Zang, Z.,Tost, H.,Schwarz, A. J.,Meyer-Lindenberg, A.	Acute ketamine challenge increases resting state prefrontal-hippocampal connectivity in both humans and rats	2015
13158	Hamilton, C.,Ma, Y.,Zhang, N.	Global reduction of information exchange during anesthetic-induced unconsciousness	2017
14685	Hempel, E.,Reith, W.,Elste, V.,Heiland, S.,Sartor, K.	Influence of stimulus frequency, amplitude and blood pressure on signal change in fMRI	1999
13930	Herman, P., Sanganahalli, B. G., Hyder, F., Eke, A.	Fractal analysis of spontaneous fluctuations of the BOLD signal in rat brain	2011
14258	Herman, P., Sanganahalli, B., Hyder, F., Eke, A.	Non-invasive hypotensive FMRI study in rat somatosensory cortex	2007
16784	Houston, G. C., Papadakis, N. G.,Carpenter, T. A.,Hall, L. D.,Mukherjee, B.,James, M. F.,Huang, C. L. H.	Mapping of the cerebral response to hypoxia measured using graded asymmetric spin echo EPI	2000
14702	Hsu, E. W., Hedlund, L. W., MacFall, J. R.	Functional MRI of the rat somatosensory cortex: Effects of hyperventilation	1998

13729	Huang, S.,Du, F.,Shih, Y. Y. I.,Shen, Q.,Gonzalez-Lima, F.,Duong, T. Q.	Methylene blue potentiates stimulus-evoked fMRI responses and cerebral oxygen consumption during normoxia and hypoxia	2013
13399	Hudetz, A. G.,Liu, X.,Pillay, S.	Dynamic repertoire of intrinsic brain states is reduced in propofol-induced unconsciousness	2015
13234	Hudetz, A. G.,Liu, X.,Pillay, S.,Boly, M.,Tononi, G.	Propofol anesthesia reduces Lempel-Ziv complexity of spontaneous brain activity in rats	2016
14056	Hutchison, R. M., Mirsattari, S. M.,Jones, C. K.,Gati, J. S.,Leung, L. S.	Functional networks in the anesthetized rat brain revealed by independent component analysis of resting-state fMRI	2010
14238	Huttunen, J. K., Gröhn, O., Penttonen, M.	Coupling between simultaneously recorded BOLD response and neuronal activity in the rat somatosensory cortex	2008
13542	Jonckers, E.,Palacios, R. D.,Shah, D.,Guglielmetti, C.,Verhoye, M.,Van Der Linden, A.	Different anesthesia regimes modulate the functional connectivity outcome in mice	2014
14423	Kalisch, R.,Delfino, M.,Murer, M. G.,Auer, D. P.	The phenylephrine blood pressure clamp in pharmacologic magnetic resonance imaging: Reduction of systemic confounds and improved detectability of drug-induced BOLD signal changes	2005
14615	Kalisch, R., Elbel, G. K., Gössl, C.,Czisch, M.,Auer, D. P.	Blood pressure changes induced by arterial blood withdrawal influence bold signal in anesthesized rats at 7 Tesla: Implications for pharmacologic MRI	2001
13715	Kalthoff, D.,Po, C.,Wiedermann, D.,Hoehn, M.	Reliability and spatial specificity of rat brain sensorimotor functional connectivity networks are superior under sedation compared with general anesthesia	2013
13996	Kalthoff, D., Seehafer, J. U.,Po, C.,Wiedermann, D.,Hoehn, M.	Functional connectivity in the rat at 11.7T: Impact of physiological noise in resting state fMRI	2011
14589	Kannurpatti, S. S., Biswal, B. B.,Hudetz, A. G.	Differential fMRI-BOLD signal response to apnea in humans and anesthetized rats	2002
14502	Kannurpatti, S. S., Biswal, B. B.	Effect of anesthesia on CBF, MAP and fMRI-BOLD signal in response to apnea	2004

14544	Kannurpatti, S. S., Biswal, B. B.,Hudetz, A. G.	Baseline physiological state and the fMRI-BOLD signal response to apnea in anesthetized rats	2003
14548	Kannurpatti, S. S., Biswal, B. B.,Hudetz, A. G.	Regional dynamics of the fMRI-BOLD signal response to hypoxia-hypercapnia in the rat brain	2003
14219	Kannurpatti, S. S., Biswal, B. B.,Kim, Y. R.,Rosen, B. R.	Spatio-temporal characteristics of low-frequency BOLD signal fluctuations in isoflurane-anesthetized rat brain	2008
14734	Kida, I.,Yamamoto, T.,Tamura, M.	Interpretation of BOLD MRI signals in rat brain using simultaneously measured near-infrared spectrophotometric information	1996
13472	Kundu, P., Santin, M. D., Bandettini, P. A., Bullmore, E. T., Petiet, A.	Differentiating BOLD and non-BOLD signals in fMRI time series from anesthetized rats using multi-echo EPI at 11.7T	2014
14401	Kuo, C. C.,Chen, J. H.,Tsai, C. Y.,Liang, K. C.,Yen, C. T.	BOLD signals correlate with ensemble unit activities in rat's somatosensory cortex	2005
14688	Lahti, K. M.,Ferris, C. F.,Li, F.,Sotak, C. H.,King, J. A.	Comparison of evoked cortical activity in conscious and propofol-anesthetized rats using functional MRI	1999
13403	Lai, H. Y., Albaugh, D. L., Kao, Y. C. J., Younce, J. R.,Shih, Y. Y. I.	Robust deep brain stimulation functional MRI procedures in rats and mice using an MR-compatible tungsten microwire electrode	2015
13903	Liang, Z.,King, J.,Zhang, N.	Anticorrelated resting-state functional connectivity in awake rat brain	2012
13843	Liang, Z.,King, J.,Zhang, N.	Intrinsic organization of the anesthetized brain	2012
13637	Liang, Z.,Li, T.,King, J.,Zhang, N.	Mapping thalamocortical networks in rat brain using resting-state functional connectivity	2013
13422	Liang, Z.,Liu, X.,Zhang, N.	Dynamic resting state functional connectivity in awake and anesthetized rodents	2015
13361	Liang, Z.,Watson, G. D. R.,Alloway, K. D.,Lee, G.,Neuberger, T.,Zhang, N.	Mapping the functional network of medial prefrontal cortex by combining optogenetics and fMRI in awake rats	2015

14696	Lin, W., Paczynski, R. P.,Celik, A.,Hsu, C. Y.,Powers, W. J.	Effects of acute normovolemic hemodilution on T2*-weighted images of rat brain	1998
14703	Lin, W., Paczynski, R. P.,Celik, A.,Hsu, C. Y.,Powers, W. J.	Experimental hypoxemic hypoxia: Effects of variation in hematocrit on magnetic resonance T2*-weighted brain images	1998
14716	Lin, W., Paczynski, R. P.,Celik, A.,Kuppusamy, K.,Hsu, C. Y.,Powers, W. J.	Experimental hypoxemic hypoxia: Changes in R2* of brain parenchyma accurately reflect the combined effects of changes in arterial and cerebral venous oxygen saturation	1998
14363	Littlewood, C. L.,Cash, D.,Dixon, A. L.,Dix, S. L.,White, C. T.,O'Neill, M. J.,Tricklebank, M.,Williams, S. C. R.	Using the BOLD MR signal to differentiate the stereoisomers of ketamine in the rat	2006
14382	Littlewood, C. L.,Jones, N.,O'Neill, M. J.,Mitchell, S. N.,Tricklebank, M.,Williams, S. C. R.	Mapping the central effects of ketamine in the rat using pharmacological MRI	2006
13841	Liu, X.,Li, R.,Yang, Z.,Hudetz, A. G.,Li, S. J.	Differential effect of isoflurane, medetomidine, and urethane on BOLD responses to acute levo-tetrahydropalmatine in the rat	2012
13634	Liu, X.,Pillay, S.,Li, R.,Vizuete, J. A.,Pechman, K. R.,Schmainda, K. M.,Hudetz, A. G.	Multiphasic modification of intrinsic functional connectivity of the rat brain during increasing levels of propofol	2013
13999	Liu, X.,Zhu, X. H.,Zhang, Y.,Chen, W.	Neural origin of spontaneous hemodynamic fluctuations in rats under burst-suppression anesthesia condition	2011
13703	Liu, X.,Zhu, X. H.,Zhang, Y.,Chen, W.	The change of functional connectivity specificity in rats under various anesthesia levels and its neural origin	2013
15923	Lowry, J. P.,Griffin, K.,McHugh, S. B.,Lowe, A. S.,Tricklebank, M.,Sibson, N. R.	Real-time electrochemical monitoring of brain tissue oxygen: A surrogate for functional magnetic resonance imaging in rodents	2010
14144	Lu, J.,Dai, G.,Egi, Y.,Huang, S.,Kwon, S. J.,Lo, E. H.,Kim, Y. R.	Characterization of cerebrovascular responses to hyperoxia and hypercapnia using MRI in rat	2009

14562	Luo, F.,Wu, G.,Li, Z.,Li, S. J.	Characterization of effects of mean arterial blood pressure induced by cocaine and cocaine methiodide on bold signals in rat brain	2003
13170	Ma, Y.,Hamilton, C.,Zhang, N.	Dynamic Connectivity Patterns in Conscious and Unconscious Brain	2017
14246	Maandag, N. J. G., Coman, D.,Sanganahalli, B. G.,Herman, P.,Smith, A. J.,Blumenfeld, H.,Shulman, R. G.,Hyder, F.	Energetics of neuronal signaling and fMRI activity	2007
13523	Magnuson, M. E.,Thompson, G. J.,Pan, W. J.,Keilholz, S. D.	Time-dependent effects of isoflurane and dexmedetomidine on functional connectivity, spectral characteristics, and spatial distribution of spontaneous BOLD fluctuations	2014
15316	Mechling, A. E., Hubner, N. S.,Lee, H. L.,Hennig, J.,von Elverfeldt, D.,Harsan, L. A.	Fine-grained mapping of mouse brain functional connectivity with resting-state fMRI	2014
13976	Min, D. K.,Tuor, U. I.,Chelikani, P. K.	Gastric distention induced functional magnetic resonance signal changes in the rodent brain	2011
13547	Nasrallah, F. A.,Lew, S. K.,Low, A. S. M.,Chuang, K. H.	Neural correlate of resting-state functional connectivity under α2 adrenergic receptor agonist, medetomidine	2014
13464	Nasrallah, F. A.,Low, S. M. A.,Lew, S. K.,Chen, K.,Chuang, K. H.	Pharmacological insight into neurotransmission origins of resting-state functional connectivity: α2-adrenergic agonist vs antagonist	2014
13890	Nasrallah, F. A.,Tan, J.,Chuang, K. H.	Pharmacological modulation of functional connectivity: α 2-adrenergic receptor agonist alters synchrony but not neural activation	2012
13533	Nasrallah, F. A., Tay, H. C., Chuang, K. H.	Detection of functional connectivity in the resting mouse brain	2014
13362	Nasrallah, F. A., Yeow, L. Y., Biswal, B., Chuang, K. H.	Dependence of BOLD signal fluctuation on arterial blood CO<inf>2</inf> and O<inf>2</inf>: Implication for resting-state functional connectivity	2015
13309	Paasonen, J.,Salo, R. A.,Huttunen, J. K.,Gröhn, O.	Resting-state functional MRI as a tool for evaluating brain	2016

		hemodynamic responsiveness to external stimuli in rats	
13282	Paasonen, J.,Salo, R. A.,Shatillo, A.,Forsberg, M. M.,Närväinen, J.,Huttunen, J. K.,Gröhn, O.	Comparison of seven different anesthesia protocols for nicotine pharmacologic magnetic resonance imaging in rat	2016
13948	Pan, W. J.,Thompson, G.,Magnuson, M.,Majeed, W.,Jaeger, D.,Keilholz, S.	Broadband Local Field Potentials Correlate with Spontaneous Fluctuations in Functional Magnetic Resonance Imaging Signals in the Rat Somatosensory Cortex Under Isoflurane Anesthesia	2011
14131	Pawela, C. P., Biswal, B. B.,Hudetz, A. G.,Schulte, M. L.,Li, R.,Jones, S. R.,Cho, Y. R.,Matloub, H. S.,Hyde, J. S.	A protocol for use of medetomidine anesthesia in rats for extended studies using task-induced BOLD contrast and resting-state functional connectivity	2009
14617	Peeters, R. R., Tindemans, I.,De Schutter, E.,Van der Linden, A.	Comparing BOLD fMRI signal changes in the awake and anesthetized rat during electrical forepaw stimulation	2001
22791	Prielmeier, F., Merboldt, K. D., Hanicke, W.,Frahm, J.	Dynamic high-resolution MR imaging of brain deoxygenation during transient anoxia in the anesthetized rat	1993
22637	Prielmeier, F., Nagatomo, Y.,Frahm, J.	Cerebral blood oxygenation in rat brain during hypoxic hypoxia. Quantitative MRI of effective transverse relaxation rates	1994
14304	Qiao, M., Rushforth, D.,Wang, R.,Shaw, R. A.,Tomanek, B.,Dunn, J. F.,Tuor, U. I.	Blood-oxygen-level-dependent magnetic resonance signal and cerebral oxygenation responses to brain activation are enhanced by concurrent transient hypertension in rats	2007
14409	Ramos-Cabrer, P.,Weber, R.,Wiedermann, D.,Hoehn, M.	Continuous noninvasive monitoring of transcutaneous blood gases for a stable and persistent BOLD contrast in fMRI studies in the rat	2005
24362	Sanganahalli, B. G.,Herman, P.,Blumenfeld, H.,Hyder, F.	FMRI and electrophysiological studies with α-chloralose and domitor anesthesia	2009
13364	Schlegel, F.,Schroeter, A.,Rudin, M.	The hemodynamic response to somatosensory stimulation in mice	2015

		depends on the anesthetic used: Implications on analysis of mouse fMRI data	
14378	Schmidt, K. F.,Febo, M.,Shen, Q.,Luo, F.,Sicard, K. M.,Ferris, C. F.,Stein, E. A.,Duong, T. Q.	Hemodynamic and metabolic changes induced by cocaine in anesthetized rat observed with multimodal functional MRI	2006
13122	Schroeter, A.,Grandjean, J.,Schlegel, F.,Saab, B. J.,Rudin, M.	Contributions of structural connectivity and cerebrovascular parameters to functional magnetic resonance imaging signals in mice at rest and during sensory paw stimulation	2017
13493	Schroeter, A.,Schlegel, F.,Seuwen, A.,Grandjean, J.,Rudin, M.	Specificity of stimulus-evoked fMRI responses in the mouse: The influence of systemic physiological changes associated with innocuous stimulation under four different anesthetics	2014
15189	Sedlacik, J.,Reitz, M.,Bolar, D. S.,Adalsteinsson, E.,Schmidt, N. O.,Fiehler, J.	Correlation of oxygenation and perfusion sensitive MRI with invasive micro probe measurements in healthy mice brain	2015
13247	Shah, D., Blockx, I., Keliris, G. A.,Kara, F.,Jonckers, E.,Verhoye, M.,Van der Linden, A.	Cholinergic and serotonergic modulations differentially affect large-scale functional networks in the mouse brain	2016
14437	Sicard, K. M.,Duong, T. Q.	Effects of hypoxia, hyperoxia, and hypercapnia on baseline and stimulus-evoked BOLD, CBF, and CMRO2 in spontaneously breathing animals	2005
14558	Sicard, K.,Shen, Q.,Brevard, M. E.,Sullivan, R.,Ferris, C. F.,King, J. A.,Duong, T. Q.	Regional cerebral blood flow and BOLD responses in conscious and anesthetized rats under basal and hypercapnic conditions: Implications for functional MRI studies	2003
13123	Smith, J. B.,Liang, Z.,Watson, G. D. R.,Alloway, K. D.,Zhang, N.	Interhemispheric resting-state functional connectivity of the claustrum in the awake and anesthetized states	2017
14121	Sommers, M. G.,van Egmond, J.,Booij, L. H. D. J.,Heerschap, A.	Isoflurane anesthesia is a valuable alternative for α-chloralose anesthesia in the forepaw stimulation model in rats	2009

13891	Sumiyoshi, A.,Suzuki, H.,Ogawa, T.,Riera, J. J.,Shimokawa, H.,Kawashima, R.	Coupling between gamma oscillation and fMRI signal in the rat somatosensory cortex: Its dependence on systemic physiological parameters	2012
14539	Tenney, J. R.,Duong, T. Q.,King, J. A.,Ludwig, R.,Ferris, C. F.	Corticothalamic modulation during absence seizures in rats: A functional MRI assessment	2003
13198	Tomimatsu, Y.,Cash, D.,Suzuki, M.,Suzuki, K.,Bernanos, M.,Simmons, C.,Williams, S. C. R.,Kimura, H.	TAK-063, a phosphodiesterase 10A inhibitor, modulates neuronal activity in various brain regions in phMRI and EEG studies with and without ketamine challenge	2016
13199	Tsurugizawa, T.,Takahashi, Y.,Kato, F.	Distinct effects of isoflurane on basal BOLD signals in tissue/vascular microstructures in rats	2016
14084	Tsurugizawa, T., Uematsu, A., Uneyama, H.,Torii, K.	Effects of isoflurane and alpha-chloralose anesthesia on BOLD fMRI responses to ingested l-glutamate in rats	2010
13911	Tu, Y., Yu, T.,Fu, X. Y.,Xie, P.,Lu, S.,Huang, X. Q.,Gong, Q. Y.	Altered thalamocortical functional connectivity by propofol anesthesia in rats	2011
14569	Tuor, U. I.,McKenzie, E.,Tomanek, B.	Functional magnetic resonance imaging of tonic pain and vasopressor effects in rats	2002
14268	Tuor, U. I.,Wang, R.,Zhao, Z.,Foniok, T.,Rushforth, D.,Wamsteeker, J. I.,Qiao, M.	Transient hypertension concurrent with forepaw stimulation enhances functional MRI responsiveness in infarct and peri-infarct regions	2007
14381	Vanhoutte, G., Verhoye, M.,Van Der Linden, A.	Changing body temperature affects the T2* signal in the rat brain and reveals hypothalamic activity	2006
29730496	W. Gsell, A. Giarola, T. Reese, A. J. Schwarz, H. Barjat, S. Smart, A. Gozzi, S. Bertani, V. Crestan, A. Bifone	Carry-over effects of gaseous anaesthesia on fMRI response and tissue oxygen levels in the rat brain	-
14009	Wang, K.,Van Meer, M. P.,Van Der Marel, K.,Van Der Toorn, A.,Xu, L.,Liu, Y.,Viergever, M. A.,Jiang, T.,Dijkhuizen, R. M.	Temporal scaling properties and spatial synchronization of spontaneous blood oxygenation level-dependent (BOLD) signal fluctuations in rat sensorimotor network at different levels of isoflurane anesthesia	2011
14377	Wang, R.,Foniok, T.,Wamsteeker, J. I.,Qiao,	Transient blood pressure changes affect the functional magnetic	2006

	M.,Tomanek, B.,Vivanco, R. A.,Tuor, U. I.	resonance imaging detection of cerebral activation	
14388	Weber, R., Ramos-Cabrer, P.,Wiedermann, D.,Van Camp, N.,Hoehn, M.	A fully noninvasive and robust experimental protocol for longitudinal fMRI studies in the rat	2006
14036	Williams, K. A., Magnuson, M., Majeed, W., LaConte, S. M.,Peltier, S. J.,Hu, X.,Keilholz, S. D.	Comparison of α-chloralose, medetomidine and isoflurane anesthesia for functional connectivity mapping in the rat	2010
13959	Wilson, D. A., Hoptman, M. J., Gerum, S. V., Guilfoyle, D. N.	State-dependent functional connectivity of rat olfactory system assessed by fMRI	2011
13145	Wu, T.,Grandjean, J.,Bosshard, S. C.,Rudin, M.,Reutens, D.,Jiang, T.	Altered regional connectivity reflecting effects of different anaesthesia protocols in the mouse brain	2017
14654	Xu, H.,Li, S. J.,Bodurka, J.,Zhao, X.,Xi, Z. X.,Stein, E. A.	Heroin-induced neuronal activation in rat brain assessed by functional MRI	2000
13203	Yoshida, K., Mimura, Y., Ishihara, R., Nishida, H., Komaki, Y., Minakuchi, T., Tsurugizawa, T., Mimura, M.,Okano, H.,Tanaka, K. F.,Takata, N.	Physiological effects of a habituation procedure for functional MRI in awake mice using a cryogenic radiofrequency probe	2016
14670	Zaharchuk, G.,Mandeville, J. B.,Bogdanov Jr, A. A.,Weissleder, R.,Rosen, B. R.,Marota, J. J. A.	Cerebrovascular dynamics of autoregulation and hypoperfusion: An MRI study of CBF and changes in total and microvascular cerebral blood volume during hemorrhagic hypotension	1999
13265	Zhurakovskaya, E., Paasonen, J., Shatillo, A.,Lipponen, A.,Salo, R.,Aliev, R.,Tanila, H.,Gröhn, O.	Global functional connectivity differences between sleep-like states in urethane anesthetized rats measured by fMRI	2016
